# Selective Polymerization Catalysis from Monomer Mixtures: Using a Commercial Cr‐Salen Catalyst To Access ABA Block Polyesters

**DOI:** 10.1002/anie.201801400

**Published:** 2018-04-27

**Authors:** Tim Stößer, Charlotte K. Williams

**Affiliations:** ^1^ Department of Chemistry Oxford University Chemical Research Laboratory 12 Mansfield Road Oxford OX1 3TA UK

**Keywords:** block copolyesters, chromium salen catalysts, ring-opening copolymerization, ring-opening polymerization, switchable catalysis

## Abstract

ABA triblock polyesters are synthesized using a commercially available chromium salen catalyst, in one pot, from monomer mixtures comprising epoxide, anhydride and lactone. The catalysis is highly selective and applies a single catalyst in two distinct pathways. It occurs first by epoxide/anhydride ring‐opening copolymerization and subsequently by lactone ring‐opening polymerization. It is used to produce various new ABA polyester polyols; these polyols can undergo post‐functionalization and chain‐extension reactions. The ability to use a commercial catalyst and switchable catalysis with monomer mixtures is expected to facilitate future explorations of new classes of block polymers.

In polymerization catalysis, the ability to selectively control (block) sequence using monomer mixtures remains a significant challenge.^1^ Nature overcomes this problem with exquisite selectivity and catalyzes thousands of reactions to form different biopolymers, including sugars, peptides, and DNA, where precisely defined sequence determines function. Synthetic mimics of biosynthesis are most successful when sequential monomer coupling reactions are applied, analogous to artificial peptide synthesis.^2^ Nonetheless, such processes are not especially suitable for synthetic polymer production—they are generally too labor intensive and time‐consuming to evaluate new block copolymers.

This work describes a method to prepare block polyesters, which are relevant as degradable and, in some cases, bio‐renewable materials useful as elastomers, fibers, healthcare materials, and drug‐delivery vectors and in electronics.^3^ Generally, block polyesters are synthesized by sequential polymerization reactions and/or with macro‐initiators—such methods can be limited by the conversion efficiency, intermediary purification steps, and by the nature of the repeat unit chemistry. An attractive alternative would be to develop catalytic processes that selectively enchain particular blocks, but such switchable catalysis remains under‐developed. In 1985, Inoue and co‐workers reported an Al‐porphyrin catalyst able to prepare block polyesters from mixtures of epoxide, anhydride, and lactone.^4^ Nonetheless, understanding the reaction was hindered as the sole lactone investigated was β‐butyrolactone, which can ring‐open at two sites complicating enchainment mechanisms. In 2014, we reported switch catalysis using a single homogeneous dizinc catalyst which selectively polymerized mixtures of lactone (CL), epoxide (CHO), and carbon dioxide to form single‐block polymer structures.^5^ Experimental and theoretical studies suggested that the selectivity resulted from kinetic and thermodynamic control by the metal–chain end group.^6^ This process is different from terpolymerization because two different polymerization cycles are accessed and the dominant catalytic cycle is switched by the chemistry of the catalyst–polymer chain end group. It is also preferable to tandem or multifunctional catalysis because a single catalyst is active in both catalytic cycles.^7^ Very recently, Rieger and co‐workers demonstrated switch catalysis, using a dizinc β‐diiminate catalyst, to prepare block/random copolymers from β‐butyrolactone, cyclohexene, oxide and carbon dioxide.^8^ It is important to understand the generality of this new switch catalysis, particularly its applicability beyond zinc complexes. Its uptake should be facilitated and accelerated by determining whether it also applies to commercially available polymerization catalysts.

The process requires a single catalyst active for both lactone ROP and epoxide/anhydride ROCOP (Scheme 1). We targeted a commercial Cr‐salen catalyst [SalcyCrCl], which is applied with equimolar addition of co‐catalyst (PPNCl).7b,7d,7f, 9 The catalyst system has precedent for other alternating copolymerizations (ROCOP),7b,7d,7f,7h, 9 and related systems are active in cyclic carbonate ROP, β‐butyrolactone ROP, and for the copolymerization of dihydrocoumarin and propylene oxide.9d, 10 Firstly, the Cr catalyst system was tested separately for ROCOP and ROP, under conditions relevant to subsequent switch catalysis (Table S1). Each polymerization occurred with high conversion and selectivity, but the resulting polyesters showed bimodal molar mass distributions; indeed similar bimodality was observed previously for CO_2_/CHO ROCOP using Cr‐salen catalysts.7h To control the molar mass distribution, polymerizations were conducted under immortal conditions, that is, with the addition of various amounts of 1,2‐cyclohexanediol (CHD, 5–20 equiv) (Figure S1). The excess alcohol controls molar mass via rapid and reversible exchange equilibria; the use of a diol ensures formation of ABA triblock copolymers. Under optimized conditions (>10 equiv CHD), both DL ROP and NBA/CHO ROCOP formed polyesters showing monomodal molar mass distributions with predominantly dihydroxyl chain end groups (Figures S2 and S3). It should be noted that a small fraction (<10 mol %) of chloro‐initiated chains should be present; these chains do not affect the GPC traces but were detected by MALDI‐ToF (Figure S3).

**Scheme 1:**
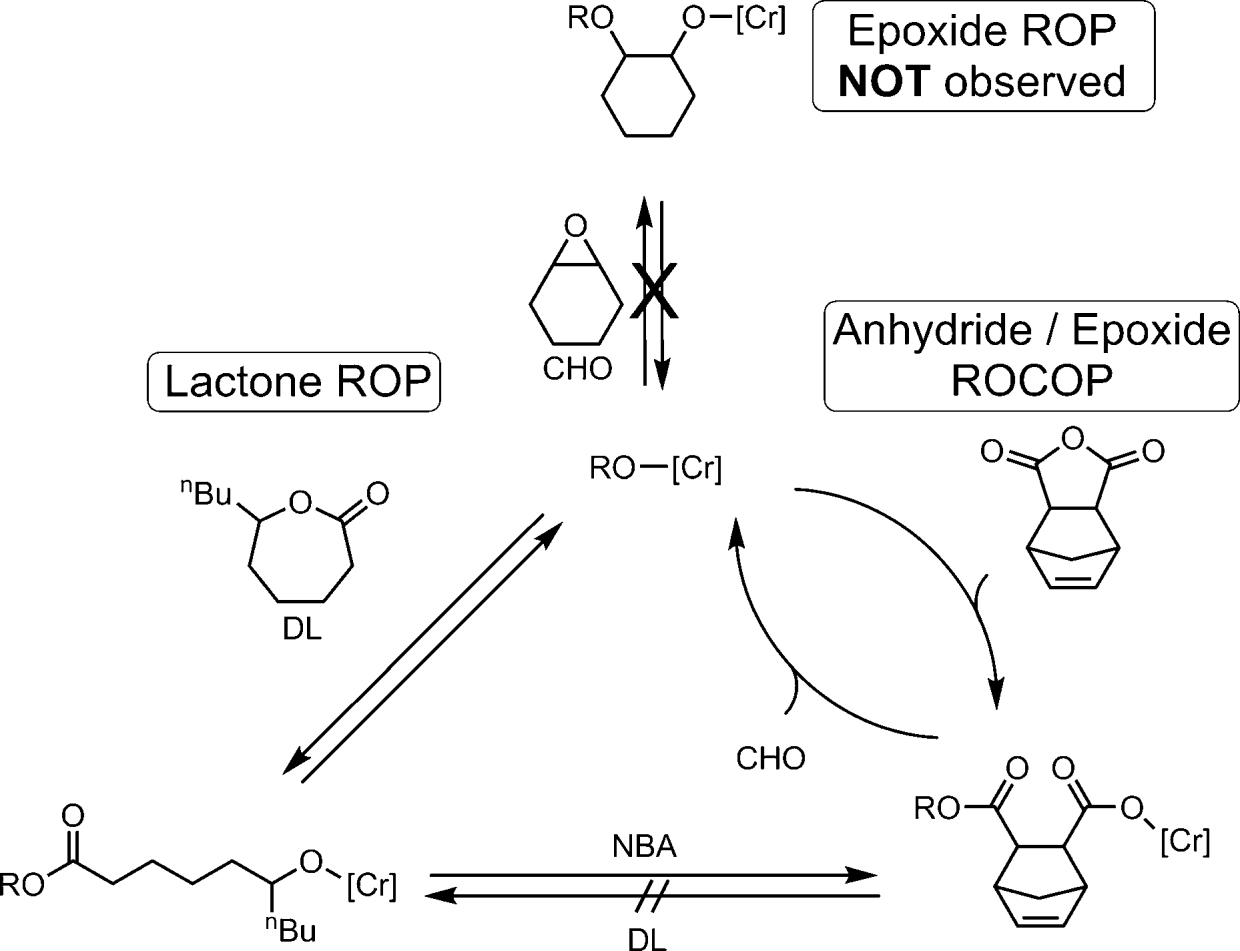
The switch catalysis pathways proposed using mixtures of DL, NBA, and CHO.

Having demonstrated that Cr‐salen catalysts were active for the separate polymerizations, catalysis using mixtures of DL/CHO/NBA, again with 10 equiv CHD, was investigated. The reaction was successful and ABA‐type polyesters were formed with narrow, monomodal molar mass distributions (Figure S1; A=PDL, B=PCHNBE). The reaction was monitored with regular removal of aliquots, which were analyzed using ^1^H NMR spectroscopy to determine conversion; this was achieved by integration of monomer signals vs. an internal standard (Figure 1, Figures S6 and S7). Over the first 1.5 h, only CHO/NBA ROCOP occurred producing the alternating polyester (PCHNBE). The high selectivity was evidenced by a rapid reduction in anhydride concentration and the concomitant growth of signals assigned to PCHNBE. The ROCOP catalysis was quite efficient, showing a TOF of ca. 67 h^−1^. Importantly, over this time period there was almost no change in the signals assigned to the lactone (<3 % by NMR, Table S2) and no evidence for any epoxide homopolymerization. After 1.5 h, the anhydride was fully consumed and subsequently DL ROP occurred slowly, reaching completion after 48 h (Figure 1, TOF≈3 h^−1^). The resulting polymer contains 66 % *cis* ester functionalities, and 34 % isomerized *trans* ester linkages, as determined by analysis of the polyester degradation products (Figure S9).

**Figure 1:**
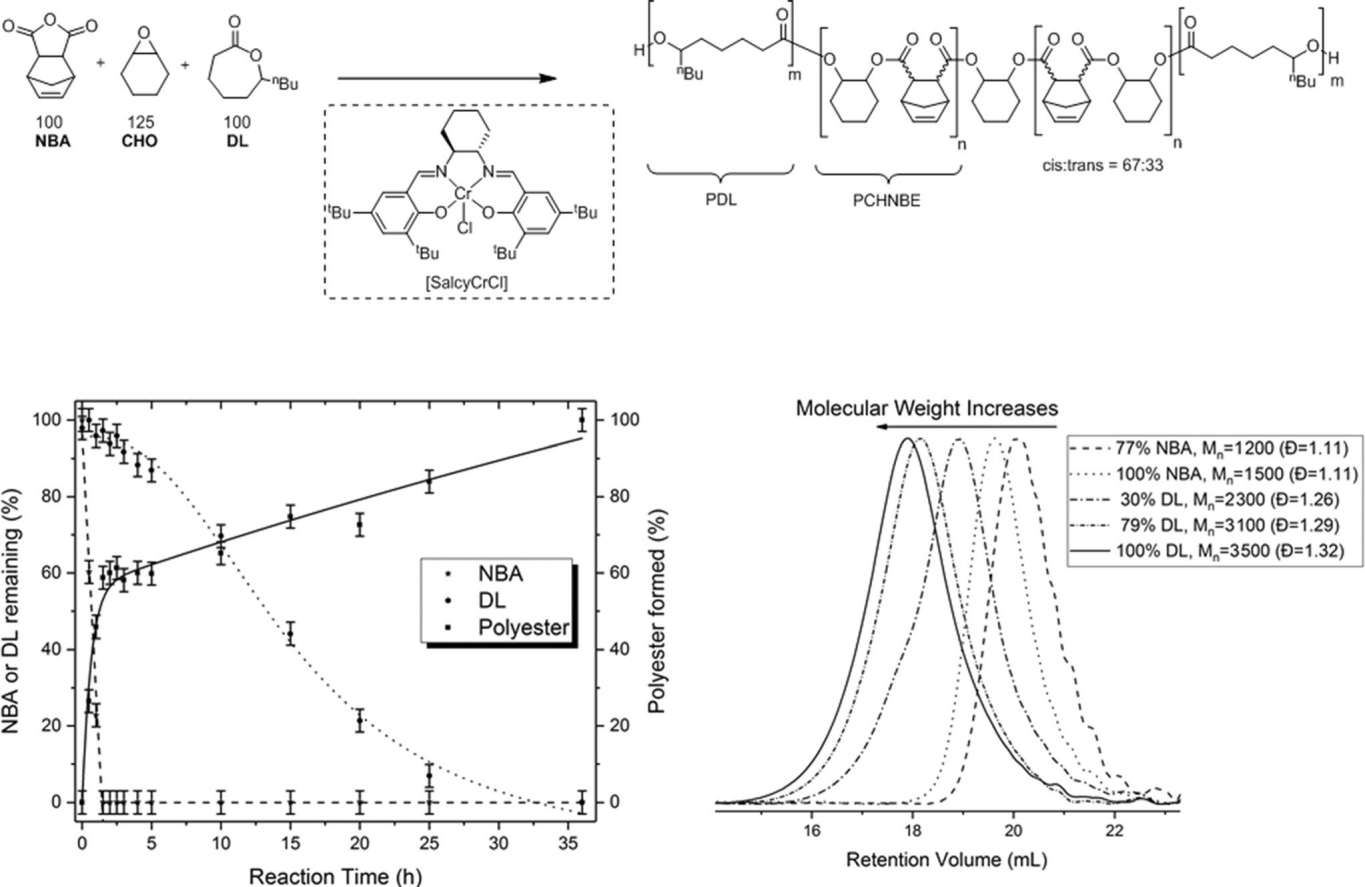
Polymerizations using mixtures of NBA, CHO, and DL to form block polyesters (top). Reaction conditions: [SalcyCrCl]/PPNCl/CHD/NBA/CHO/DL=1:1:10:100:125:100, Tol‐d_8_ (2.5 m), 100 °C. Polymerization conversion vs. time plots (bottom, left) and evolution of molar mass vs. conversion as illustrated by GPC data (*M_n_* values are obtained using calibration against PS standards). Conversion vs. time data are obtained by analysis of the ^1^H NMR integrals, using mesitylene as an internal standard (Figures S4 and S5, Table S2).

The spectroscopic data and TOF values clearly indicate the high catalytic selectivity for CHO/NBA/DL, with ROCOP occurring before ROP, but such selectivity could form either triblock polyester (PDL‐*b*‐PCHNBE‐*b*‐PDL) or a mixture of both polymers (PCHNBE + PDL). In order to characterize the polymer composition, aliquots were analyzed by GPC (Figure 1). The molar masses increase with conversion and in all cases samples show narrow, monomodal distributions (1.11<Ð<1.32); such data are strongly indicative of block polyester formation. Signals corresponding to both blocks were observed by ^1^H NMR spectroscopy and ^13^C{^1^H} NMR spectroscopy (Figures S10–S14). The ^13^C{^1^H} NMR spectrum also showed low‐intensity signals at intermediate chemical shifts which are assigned to the minor epimerized isomer (Figure S13). Such signals are not attributed to transesterification since reaction of the polymer with an efficient transesterification catalyst (DBU) significantly increased both the number and intensity of intermediary chemical shift signals (Figure S15). Furthermore, the switch catalysis was also generalized to produce a block polyester from PA/CHO/DL; its ^13^C{^1^H} NMR spectrum showed only signals for the two blocks with no intermediate signals, consistent with a lack of transesterification reactions (Figures S26–S29).6a The DOSY NMR spectrum of the polymer formed from DL/CHO/NBA shows a single diffusion coefficient for all signals, consistent with block polyester formation. The analogous blend of constituent polymers shows two diffusion coefficients (Figure S16). The reaction of its hydroxyl end groups with 2‐chloro‐4,4,5,5‐tetramethyldioxaphospholane enabled analysis by ^31^P{^1^H} NMR spectroscopy to differentiate the end groups.9b The homopolymers show different signals (146.5=PCHPE and 147.1 ppm=PDL) and the block polymer shows only one signal, at 147.1 ppm, consistent with its ABA structure (Figures S17 and S18, Table S3). Finally, the crude block polymer was purified using hexane, which is known to dissolve the homopolymer (PDL). There was no detectable homopolymer in the hexane and the composition of the block polymer remained the same before and after purifications (Figure S10). Thus, all the analytical tests confirmed the formation of single‐block polymer structure, that is, PDL‐*b*‐PCHNBE‐*b*‐PDL.

The catalysis is proposed to occur via two different polymerization cycles that are linked by a common Cr‐alkoxide intermediate (Scheme 1). It is important to note that a third cycle, involving epoxide ring‐opening polymerization (forming ether linkages), is not accessed even over prolonged reaction times, as confirmed by ^1^H NMR and MALDI analysis (Figures S4 and S5). Our hypothesis is that large differences in the rate of anhydride vs. lactone insertion and a high barrier to Cr‐carboxylate reaction with lactone (and Cr‐alkoxide with epoxide) control the selectivity. More generally, the above rationale also allows for scenarios where both polymerization rates are similar and a statistical copolyester might form (vide infra).

Next, these hypotheses were specifically tested using other monomers and conditions. Firstly, experiments were conducted to confirm the reactivity of the Cr‐carboxylate intermediate. The preliminary kinetic analyses indicate ROCOP rates that are zero order in anhydride concentration (Figures S7 and S8)—that is, during ROCOP the catalyst resting state is the Cr‐carboxylate species. To test the stability of this intermediate towards reaction with lactone, a polymerization reaction was conducted using excess anhydride and lactone vs. epoxide (DL/NBA/CHO, 200:200:125). ROCOP proceeded until the epoxide was consumed (≈60 % NBA conversion) producing only alternating polyester, PCHNBE. At this point no further conversion of any monomer occurred even over prolonged reaction times (4 days) and despite the presence of excess DL (200 equiv) (Figure S19). In order to confirm the catalyst had not decomposed, the polymerization was “switched on” by the addition of a further 200 equiv of epoxide. At this point, ROCOP was resumed until complete anhydride consumption which was followed by DL ROP leading to triblock polyester formation (Figures S20 and S21). It is important to note that the proposed pathway does not examine the intimate mechanism or active catalyst structure(s) which have been the subject of extensive investigations.7b,7d, 9b,9c, 11, 12

To test the catalytic scope, a series of triblock polyesters of differing compositions were prepared by controlling the relative amounts of monomers in the mixture. In all cases, high monomer conversions were achieved and well‐defined, low‐molar‐mass block polyesters formed (Figures S22 and S23). DSC analyses showed amorphous structures with a single glass‐transition temperature, which is indicative of block miscibility. The block polyester composition was directly controlled by the monomer composition in the mixture and glass‐transition temperatures could be tuned from −30–111 °C (Table S4 and Figures S24 and S25). Several factors contribute to (micro)‐phase separation including polymer architecture, block volume fractions, the Flory–Huggins interaction parameter (χ), and degree of polymerization.^13^ Here, the block miscibility is most likely a consequence of the low molar masses, which are expected to fall below the entanglement molar masses. Similar effects were observed for ABA poly(styrene‐*b*‐isoprene‐*b*‐styrene): at low *M_n_*, a single glass‐transition temperature was observed (*M_n_*=11 000 g mol^−1^, T_g_=38 °C), at intermediate *M_n_* values, two intermediate glass‐transition temperatures occur (*M_n_*=16 000 g mol^−1^, T_g,1_=−48 °C, T_g,2_=43 °C) whilst at higher *M_n_*, there are two glass‐transition temperatures, similar to the homopolymers (*M_n_*=161 000 g mol^−1^, T_g,1_=−65 °C, T_g,2_=105 °C).^14^

The Cr‐salen switch catalyst system was also applied to a range of different monomer mixtures; the influence of the anhydride structure was investigated (Table 1). Each reaction and polymer product was fully characterized using a range of techniques, including NMR analysis (^1^H, ^13^C, COSY, HSQC), conversion vs. time plots (using both IR and NMR data), GPC, and MALDI, and by comparison of composition before and after isolation. Table 1 provides an overview of composition and relative rate data; the complete characterization data sets for each block polymer are provided in the Supporting Information (Figures S26–S75). The majority of monomer mixtures resulted in selective formation of only ABA triblock polyesters (this applies to mixtures of PA, THPA, TCA1, or TCA2 with CHO/DL). In all these cases, fast and selective ROCOP is followed by slow ROP (<5 % DL conversion at >99 % conversion of anhydride). Analysis of the relative rates reveals that selective catalysis occurs when the rate of ROCOP is >20 times that of ROP, in line with the mechanistic hypothesis (Scheme 1). The polymerizations all proceed with high monomer conversions and form block polyesters with monomodal molar mass distributions. ABA‐type block polyester structures were confirmed through multiple experiments, analogous to the range of characterizations of PDL‐*b*‐PCHNBE‐*b*‐PDL. Moreover, in every case the molar mass increased continuously throughout the reaction. In particular, the polymerization of DL/CHO/PA results in formation of a block polyester containing an aromatic backbone group. Thus, the aliquots were analyzed by GPC with both RI and UV detectors: there was a clear evolution in molar mass using both detection methods (Figure S33). This finding confirms covalent bonding between the alternating semi‐aromatic polyester and the PDL blocks. In contrast, the monomer mixture comprising DL/CHO/camphoric anhydride (CA) showed similar rates of ROCOP and ROP and, in line with the switch hypothesis, statistical copolyesters formed (Figure S66–75).

**Table 1:** Polymerizations using mixtures of CHO, DL, and various different anhydrides.^[a]^

Anhydride (A)	A (E) conv. [%]^[b]^	DL conv. [%]^[b]^	*M_n_* (Ð)^[c]^	TOF_ROCOP_/ TOF_ROP_^[d]^
PA	99 (76)	99	6200 (1.20)	64
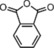				
Figs. S26–S35				
				
THPA	99 (79)	99	4000 (1.26)	48
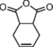				
Figs. S36–S45				
				
NBA Figs. S10–S14	99 (74)	99	3500 (1.32)	22
				
TCA1	99 (77)	99	6700 (1.20)	32
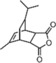				
Figs. S46–S55				
				
TCA2	99 (67)	99	5800 (1.20)	103
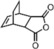				
Figs. S56–S65				
				
CA	99 (80)	99	6700 (1.14)	1.4
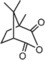				
Figs. S66–S75				

[a] Polymerization conditions: [SalcyCrCl]/[PPNCl]/[CHD]/[CHO]/[DL]/[A]=1:1:15:250:200:200, 100 °C, 24–96 h; where A refers to anhydride and E to epoxide. [b] Determined by ^1^H NMR spectroscopy; note for E: theoretical max. conversion=80 % (see relevant figures in the Supporting Information). [c] Determined by ^1^H NMR spectroscopy and MALDI‐ToF spectrometry (see relevant figures in the Supporting Information). [d] Determined by GPC, in THF at 30 °C, calibrated using polystyrene standards (see relevant figures in the Supporting Information). [e] Determined from conversion vs. time data, at >95 % monomer conversion (Tables S5–S9).

To highlight the potential for the new materials, post‐functionalization reactions, using the thiol–ene reaction, were conducted upon PDL‐*b*‐PCHNBE‐*b*‐PDL. The alkene functional groups were successfully substituted with either hydrophilic or hydrophobic side chains, as observed by ^1^H NMR spectroscopy (Figures S76, S78, and S79). In both cases, the functionalization reactions occurred without disrupting the block polymer structure as indicated by similar molar mass distributions before and after the reaction (Figure S77). Although thiol–ene reactions are well established in polymer post‐functionalization, this proof‐of‐concept highlights the future potential for these materials in coatings applications, where low molar masses would benefit processing and where multifunctional thiols are popular cross‐linking agents.^15^, ^16^ All new block polymers also have hydroxyl‐telechelic structures, that is, they are new polyester polyols. Polyester polyols are used in polyurethane production and as proof of chain extension, PDL‐b‐PCHNBE‐b‐PDL was reacted with 4,4′‐methylene diphenyl diisocyanate (MDI). The precursor material showed one glass‐transition temperature at 26 °C and contained 39 wt % PDL. After chain extension, the molar mass increased from ≈4 to ≈70 kg mol^−1^ and DSC analysis indicated phase separation, as two glass‐transition temperatures at 46 and 88 °C were observed (Figures S80 and S81). Such multiblock polymers, with controllable compositions, may be interesting as rigid plastics or thermoplastic elastomers.^17^

In conclusion, monomer mixtures can be selectively reacted with a commercially available chromium salen catalyst, in one pot, to form well‐defined ABA triblock polyesters. The scope of the catalysis is demonstrated using a range of different mixture compositions and monomers to deliver new polyester polyols. These polyesters can undergo post‐functionalization reactions to modify the side‐chain substituents or chain‐extension reactions to produce multiblock polyesters. Switch catalysis, using Cr‐salen catalysts, is expected to be applicable to the preparation of other block and multiblock polymers. The method should be applied using mixtures of carbon dioxide, lactones, anhydrides, and epoxides to produce new block polycarbonates, esters, and ethers.

## Conflict of interest

The authors declare no conflict of interest.

## Supporting information

As a service to our authors and readers, this journal provides supporting information supplied by the authors. Such materials are peer reviewed and may be re‐organized for online delivery, but are not copy‐edited or typeset. Technical support issues arising from supporting information (other than missing files) should be addressed to the authors.

Supplementary
