# Supplementary material for: Selective Polymerization Catalysis from Monomer Mixtures: Using a Commercial Cr‐Salen Catalyst To Access ABA Block Polyesters
Source: Angew Chem Int Ed Engl. 2018 Apr 27;57(21):6337–41. doi: 10.1002/anie.201801400 (PMC6391957; doi:10.1002/anie.201801400)
Supplement: Supplementary file 1 — Supplementary [file ANIE-57-6337-s001.pdf]

Angewandte Chemie
Eine Zeitschrift der Gesellschaft Deutscher Chemiker

Supporting Information

Selective Polymerization Catalysis from Monomer Mixtures: Using a Commercial Cr-Salen Catalyst To Access ABA Block Polyesters

*Tim Stößer and Charlotte K. Williams**

anie_201801400_sm_miscellaneous_information.pdf

Contents

1. Materials ... 3
2. Characterization ... 3
3. Experimental Section ... 5
4. 'Switch' catalysis of NBA, CHO and DL. ... 7
   4.1. Optimization of ROCOP and ROP ... 7
   4.2. Monitoring of Polymerization of NBA/CHO/DL ... 12
   4.3. Reaction of PDL-*b*-PCHNBE-*b*-PDL with Lithium Aluminium Hydride ... 15
   4.4. Characterization of PDL-*b*-PCHNBE-*b*-PDL ... 17
   4.5. Transesterification with DBU ... 21
   4.6. DOSY NMR Spectra of PDL-*b*-PCHNBE-*b*-PDL ... 22
   4.7. End-group Assay with $^{31}P\{^{1}H\}$ NMR spectroscopy ... 23
   4.9. Polymerization of NBA,CHO and DL with an excess of NBA ... 25
   4.10. Thermal Analysis of polyesters with different PCHNBE:PDL block ratios ... 27
5. Anhydride Scope ... 31
   5.2. PA/CHO/DL ... 31
   5.3. THPA/CHO/DL ... 40
   5.4. TCA1/CHO/DL ... 48
   5.5. TCA2/CHO/DL ... 56
   5.1. CA/CHO/DL ... 64
   5.6. End-group analysis with $^{31}P\{^{1}H\}$ NMR spectroscopy ... 71
6. Post-Modification Reactions ... 72
7. References ... 77

1. Materials

Cis-5-Norbornene-endo-2,3-dicarboxylic anhydride (99%), cyclohexene oxide (98%), ε-decalactone (99%) were purchased from Sigma Aldrich. Phthalic anhydride (99%, Aldrich) was purified by stirring in dry benzene overnight, filtering the insoluble impurities under nitrogen, concentrating to dryness, recrystallization from dry chloroform and sublimation under reduced pressure. *Trans*-1,2-cyclohexanediol (CHD, 98%, Aldrich) was recrystallized from HPLC-grade ethyl acetate, dried under high vacuum overnight and stored in a glovebox. 1-Butanethiol and 2-mercaptoethanol were dried over calcium hydride, distilled under nitrogen and stored in the glovebox. Methylene diphenyl diisocyanate was sublimed under high vacuum and stored inside the glovebox at – 30 °C. 2-Chloro-4,4,5,5-tetramethyl dioxaphospholane (95%) and 1,5,7-triazabicyclo[4.4.0]dec-5-ene (TBD, 98%) were used as received from Aldrich. All other chemicals were obtained from obtained from several commercial suppliers (Sigma Aldrich, Fischer, VWR, Alfa Aesar, Acros Organics) and all anhydrides were purified by sublimation under high vacuum. ε-Decalactone (DL) was dried over calcium hydride and purified by fractional vacuum distillation. Cyclohexene oxide was reacted with sodium hydride and methyl iodide and then purified by fractional vacuum distillation according to a procedure published by Rieger and co-workers.[1] (1R,2R)-(+)-[1,2-Cyclohexanediamino-N,N'-bis(3,5-di-t-butylsalicylidene)]chromium(III) chloride ([SalcyCrCl]) was purchased from Strem chemicals and used without further purification. Bis(triphenylphosphoranylidene)ammonium chloride (PPNCl, 97%) was obtained from Sigma Aldrich, re-crystallised from acetonitrile / diethyl ether, dried at 40°C overnight and stored in a glovebox. Dry toluene was obtained from a SPS-800 system by M Braun, degassed by bubbling with nitrogen for one hour and stored over activated molecular sieves. Toluene-d_8 and $CDCl_3$ were stirred over calcium hydride overnight, transferred under high vacuum, degassed with three freeze-pump-thaw cycles and stored over activated molecular sieves. Mesitylene (98%) was dried over calcium hydride, transferred under high vacuum, degassed by three freeze-pump-thaw cycles and stored over activated molecular sieves.

2. Characterization

In situ ATR-IR Spectroscopy

For monitoring by ATR-IR, a Mettler-Toledo ReactIR 4000 spectrometer with a MCT detector and a silver halide DiComp probe was used.

DSC

Differential scanning calometry (DSC) was performed on a Mettler Toledo DSC 3 Star calorimeter under nitrogen at a heating rate of 10 °C/min. First, the sample was kept at 25 °C for 1 minute, then heated to 160 °C at 10 °C/min, kept for 1 minute and cooled down to -90 °C and kept for 1 minute. This heating / cooling cycle was then

repeated twice and the data from the third heating cycle is reported. The flow rate of nitrogen was kept at 80 mL/min throughout the measurement.

MALDI-ToF

Mass spectrometry was done on a MALDI micro instrument with dithranol as matrix (10 mg/ml), potassium trifluoroacetic acid (10 mg/ml) or sodium trifluoroacetic acid (10 mg/ml) as additive, and THF as solvent. The polymer (ca. 10 mg/ml), the matrix and the additive were mixed in a ratio of 1/4/1 (v/v) and 5 μl were spotted on the MALDI plate. The spotting was repeated three times.

NMR

^{1}H-NMR spectra were measured on a Bruker Avance III HD nanobay NMR equipped with a 9.4T magnet (^{1}H: 400.2MHz, ^{31}P 162.0MHz) and a Bruker Avance III NMR equipped with a 11.75T magnet (^{1}H NMR: 500 MHz). ^{13}C{^{1}H}-NMR spectra were measured on a Bruker Avance NMR equipped with a 11.75T magnet and a ^{13}C{^{1}H} detect cryoprobe (^{1}H: 500.3MHz, ^{13}C: 125.8MHz). All spectra were recorded in $CDCl_3$.[2]

SEC

Size exclusion chromatography (SEC) was performed on an Agilent PL GPC-50 instrument, with HPLC grade THF, at 30°C and a flow rate of 1.0 mL/min. In all cases, near monodisperse polystyrene standards were used for calibration. The samples were prepared by dissolving ca. 20 mg of polymer in THF, and filtering through a 2 μm PTFE filter before injection.

End-group assay for hydroxyl groups with 2-chloro-4,4,5,5-tetramethyl dioxaphospholane[3]

The procedure was previously reported by Spyros and co-workers.[3] Frist, a stock solution containing Bisphenol A (400 mg), $Cr(acac)_3$ (5.5 mg) and 10 mL of pyridine was prepared. Second, the polymer was dissolved in $CDCl_3$ (50 mg/0.5 mL) and 40 μL of the stock solution were added. Finally, the phosphorus agent (30 μL) was added to an NMR tube in the glovebox, and the solution containing the sample and the stock solution was added. The mixture was allowed to react for at least 30 minutes and then analysed by ^{31}P {^{1}H} NMR spectroscopy after calibration with the internal standard (bisphenol A) which shows a peak at 138.57 ppm.

3. Experimental Section

General polymerization procedure

Inside the glovebox, [SalcyCrCl] (1 equiv., 4.4 mg, 7.0 µmol), PPNCl (1 equiv., 20 µL of a stock solution of 50 mg PPNCl in 250 µL dry acetonitrile [equivalent to 4.0 mg, 7.0 µmol]), CHD (10 equiv., 8.0 mg, 70 µmol or the amount specified) and anhydride (200 equiv., 1.39 mmol) were added to a vial. CHO (250 equiv., 180 µL, 1.74 mmol), DL (200 equiv., 240 µL, 1.39 mmol) and toluene-d_8 (550 µL to obtain a 2.5 M solution in anhydride) were then added, the vial was sealed with a melamine-cap containing a Teflon inlay and further sealed tightly with electrical tape (poly(vinyl chloride)). The vials were left to react in a silicone oil bath preheated at 100 °C and opened to air to quench the reaction. Where applicable, 50 equivalents of dry mesitylene were added as an internal standard.

To monitor the reactions, the vials were opened inside the glovebox and a small aliquot (ca. 100 µL of the reaction mixture) was taken for analysis by ^{1}H NMR or GPC. For the kinetic profile presented in Figure S 6, the scale was multiplied with a factor of 15 and 50 equivalents of dry mesitylene were added. After heating inside the glovebox to obtain a homogeneous solution, the reaction mixture was split into 20 separate vials, which were opened at the specified times. For Conversion vs. time graphs, lines of best fit were used. For thermal characterisation, the polymers were isolated by precipitation from hexane and drying at 40 °C under vacuum for several days.

General procedure for anhydrides different from NBA

Inside the glovebox, [SalcyCrCl] (1 equiv., 17.6 mg, 28.0 µmol), PPNCl (1 equiv., 16 mg, 28.0 µmol), CHD (15 equiv., 48 mg, 0.42 mmol) and anhydride (200 equiv., 5.56 mmol) were added to a vial. CHO (250 equiv., 720 µL, 6.95 mmol), DL (200 equiv., 960 µL, 5.56 mmol), toluene-d_8 (2.2 mL to obtain a 2.5 M solution in anhydride) and dry mesitylene (25 equiv., 97 µL) were then added and the vial was sealed with a melamine-cap containing a Teflon inlay and further sealed tightly with electrical tap (poly(vinyl chloride)). The vials were left to react in a silicone oil bath preheated at 100 °C for 4 days and opened to air to quench the reaction. Aliquots were taken periodically throughout the reaction by cooling the vial to room temperature, opening to nitrogen in the glovebox and ca. 0.2 mL were removed and exposed to air.

The procedure was followed with CA (1.01 g, 5.56 mmol), TCA2 (990 mg, 5.56 mmol), PA (411 mg, 5.56 mmol), THPA (422 mg, 5.56 mmol) and TCA1 (612 mg, 5.56 mmol).

General procedure for thiol-ene reactions

Inside the glovebox, the block copolyester (100 mg, M_n=4,000 g/mol (Đ=1.28), mol%(PCHNBE):mol%(PDL)=1.00:1.02 [wt % (PCHNBE):wt % (PDL) = 58:42], 1.00 equiv.), 2-2-dimethoxyphenylacetophenone (10.0 mg, 0.10 equiv.) and butanthiol (30 µL, 0.27 mmol, 10 equiv.) or mercaptoethanol (30 µL, 0.43 mmol, 15 equiv.) were dissolved in $CDCl_3$ (0.5 mL) and transferred to a vial. The vial was seled with PVC isolation tape and irradiated at room temperature with a UV lamp (Philips Facial Tanner HB172, 4x15 W) in a distance of ca. 10 cm for 2 hours. The polymers were isolated by precipitation from n-hexanes.

General procedure for chain-extension with Methylene diphenyl diisocyanate

Inside the glovebox, methylene diphenyl diisocyanate (MDI, 72 mg, 0.288 mmol, 1.10 equiv. with respect to [OH]), the block copolyester (1.0 g, M_n=4,000 g/mol (Đ=1.28), $M_{n,theo}$=8,420 g/mol,), mol%(PCHNBE):mol%(PDL)=1.00:1.02 [wt % (PCHNBE):wt % (PDL) = 58:42], 1.00 equiv.) and tin(II) 2-Ethylhexanoate (0.1 mL of a stock solution with the concentration 10 mg/mL) were added to a 25 mL Schlenk flask, dissolved in ca. 5.0 mL of toluene and heated at 60 °C for 1.5 hours. The polymer was isolated by precipitation from methanol.

4. 'Switch' catalysis of NBA, CHO and DL.

4.1. Optimization of ROCOP and ROP

Table S 1 - Optimisation of CTA for ROP, ROCOP and Switch catalysis.[a]

Polymer	Eq of CHD	Conversion (%)[b]	M_n (Đ)[c]	$M_{n,theo}$[d]
PDL	0	94	37,600 (1.02)<br>13,500 (1.21)	-
PDL	5	99	9,300 (1.07)<br>3,860 (1.07)	-
PDL	10	99	3,520 (1.15)	3,520
PDL	15	99	2,620 (1.14)	2,390
PDL	20	99	2,160 (1.15)	1,820
PCHNBE[e]	0	99	15,900 (1.11)<br>6,310 (1.07)	-
PCHNBE	5	99	8,200 (1.04)<br>3,410 (1.06)	-
PCHNBE	10	99	6,860 (1.05)<br>2,270 (1.18)	-
PCHNBE	15	99	1,640 (1.17)	3,610
PCHNBE	20	99	1,590 (1.21)	2,740
PDL-*b*-PCHNBE-*b*-PDL[f]	0	99 / 5	3,220 (1.48)	-
PDL-*b*-PCHNBE-*b*-PDL	5	99 / 97	8,740 (1.11)<br>3,270 (1.12)	-
PDL-*b*-PCHNBE-*b*-PDL	10	99 / 94	4,430 (1.20)	8,560
PDL-*b*-PCHNBE-*b*-PDL	15	99 / 95	3,140 (1.18)	5,770
PDL-*b*-PCHNBE-*b*-PDL	20	99 / 96	2,990 (1.18)	4,370

[a] Reaction conditions: DL:CHO:[SalcyCrCl]:PPNCl=200:50:1:1, Tol (2.5 M), 100°C, 3d. For PCHNBE, 250 equiv. of CHO were added. [b] Determined by ^{1}H NMR spectroscopy from the normalised integrals for DL and NBA. [c] Determined by GPC, calibrated using PS standards, in THF and at 30 °C. It should be noted that these are uncorrected values. [d] Calculated based on weight of monomers (170.25 for DL, 262.31 for NBA + CHO) and CHD end group (116.15). Example: n(CHD) + (200*conversion*Monomer)/(CTA). [e] 6d. [f] 10d.

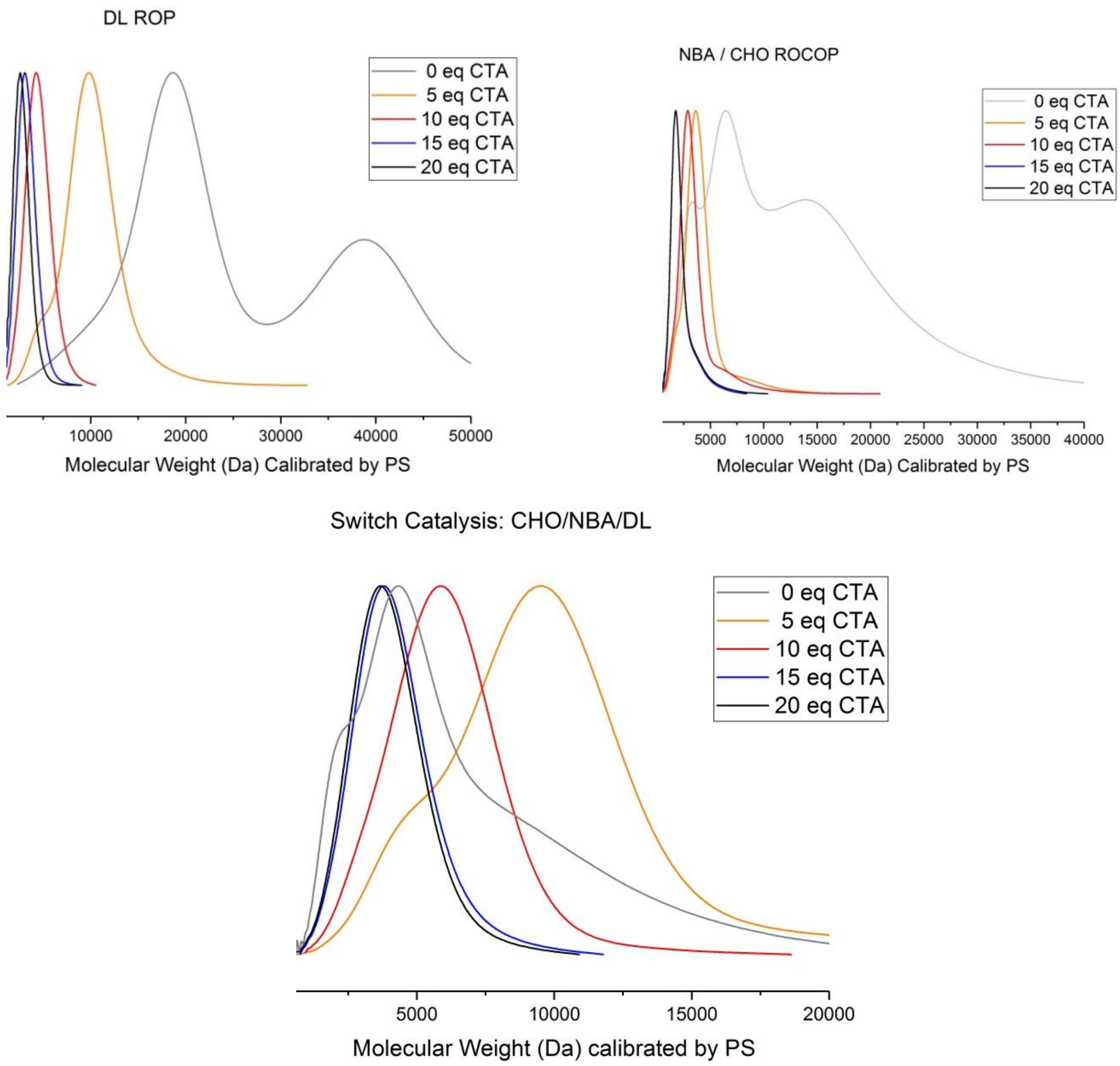

Figure S 1 – GPC traces for ROP of DL (top, left), ROCOP of NBA and CHO (top, right) and the polymerization of DL/NBA/CHO, using [SalcyCrCl] with various amounts of *trans*-1,2-cyclohexanediol (CHD) as CTA. Conversions, M_n and Đ values are given in Table S1. It should be noted that bimodal molecular weight distributions were obtained in the absence of CTA – this can be ascribed to monofunctional, chloro-initiated chains (first distribution) and dihydroxyl-terminated chains caused by residual amounts of water and / or bifunctional impurities in the monomers (i. e. residual amounts of diols for epoxides or dicarboxylic acids for anhydrides; second distribution) (see Figure S3). This same bimodality has been described for zinc and magnesium catalysts for cyclohexene oxide/phthalic anhydride ROCOP.[4]

4.2. MALDI Analysis at 40% NBA conversion (*ca.* 1 % DL conversion)

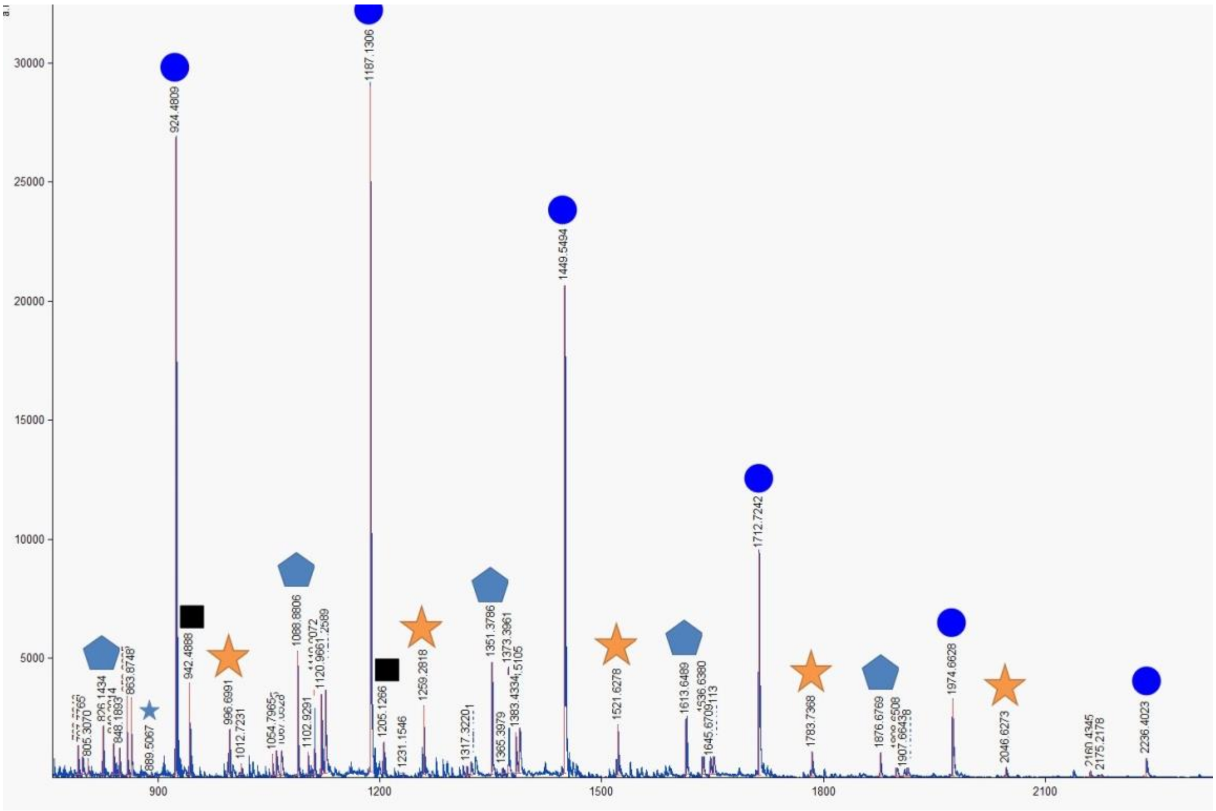

Figure S 2 – MALDI of crude sample of NBA/CHO/DL ‘Switch’ catalysis.

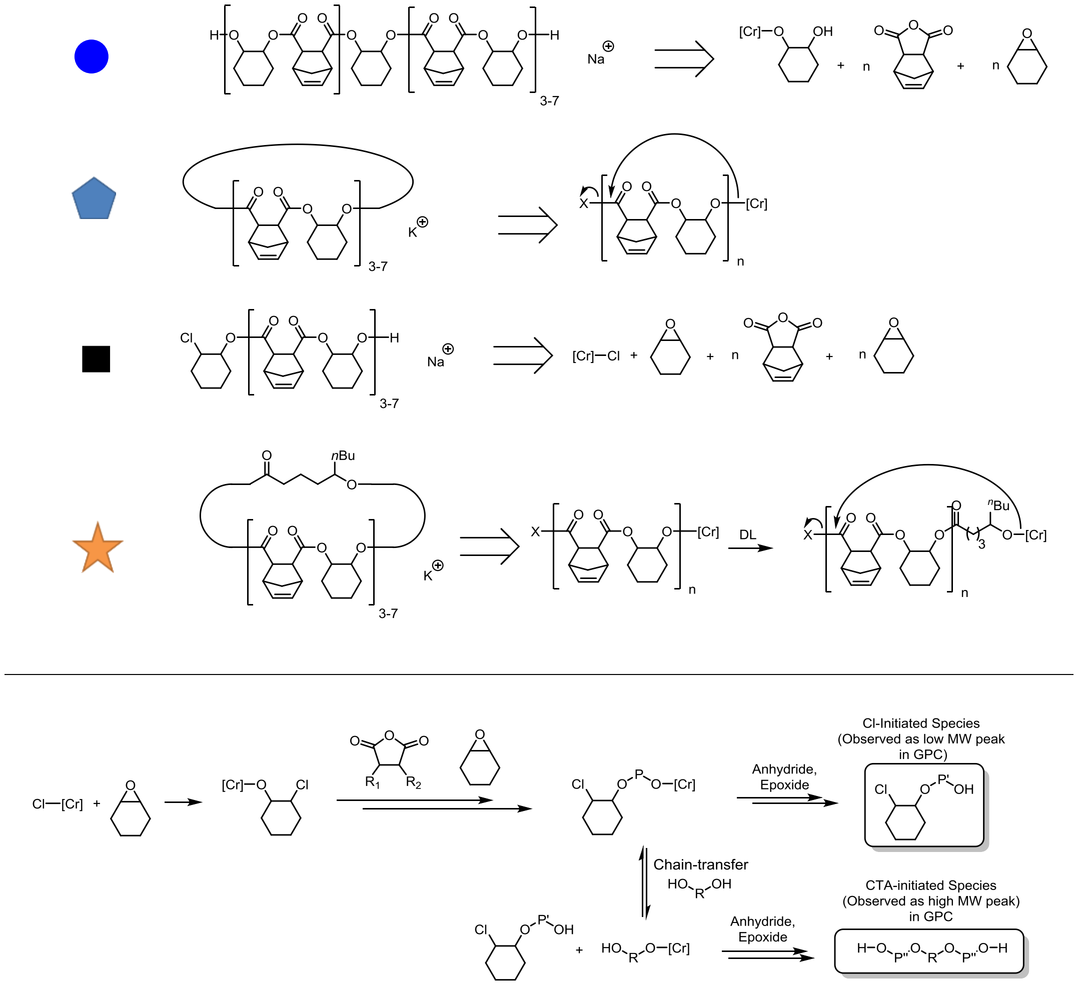

Figure S 3 – Typical region of the MALDI-ToF spectrum with assignment of species present and possible rationale for their formation (top). An illustration of the routes to formation of chloro- and dihydroxyl-terminated chains in the presence of (trace)-amounts of bifunctional impurities is provided (bottom); the co-catalyst was not taken into account.[4]

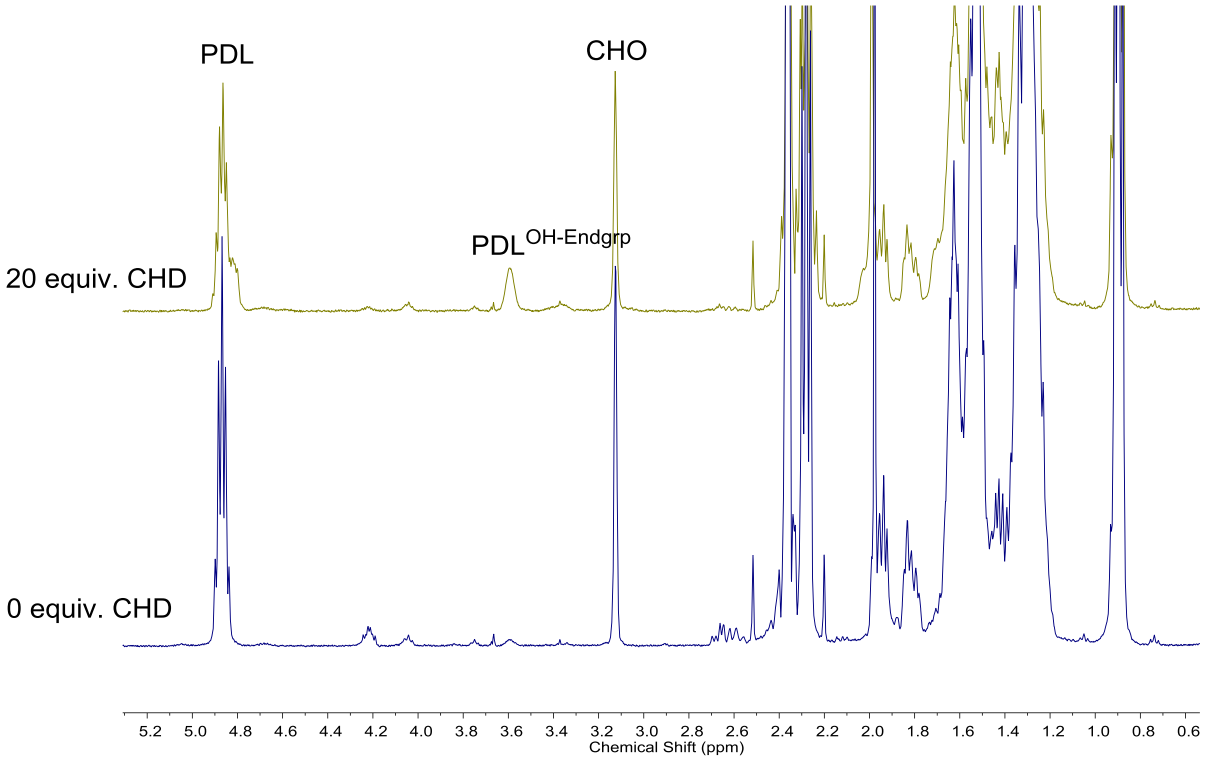

Figure S 4 - Representative ^{1}H NMR spectra of crude samples for the homopolymerisation of DL with 20 and 0 equivalents of CHD. The absence of poly(cyclohexene oxide) at 3.4 – 3.6 ppm should be noted.

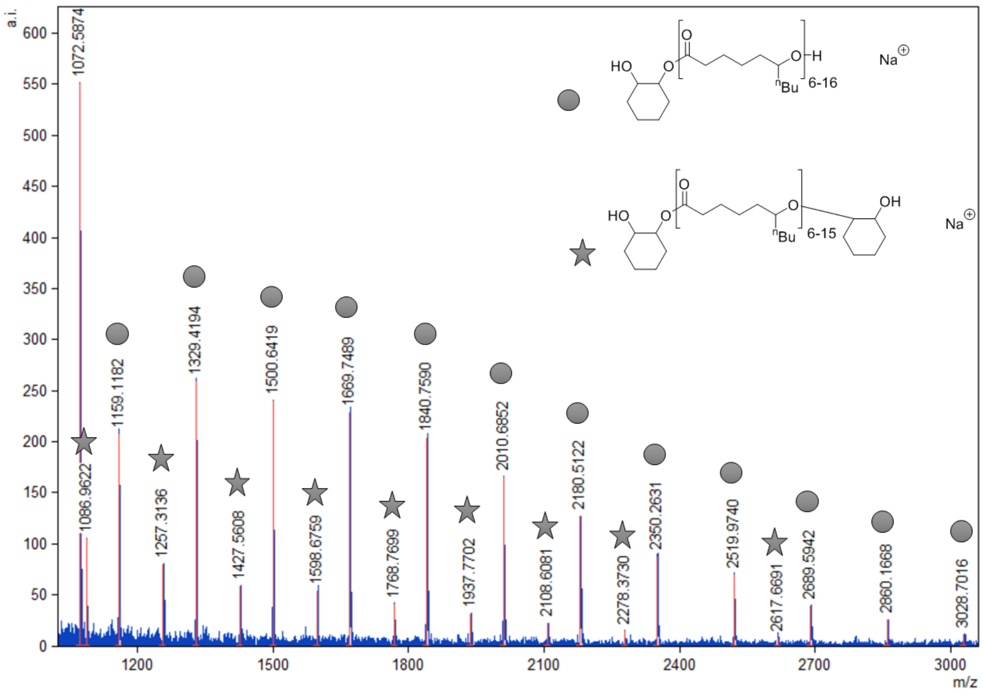

Figure S 5 - Representative MALDI spectrum for a crude sample of PDL (20 equiv. of CHD). No polyether linkages were detected.

4.2. Monitoring of Polymerization of NBA/CHO/DL

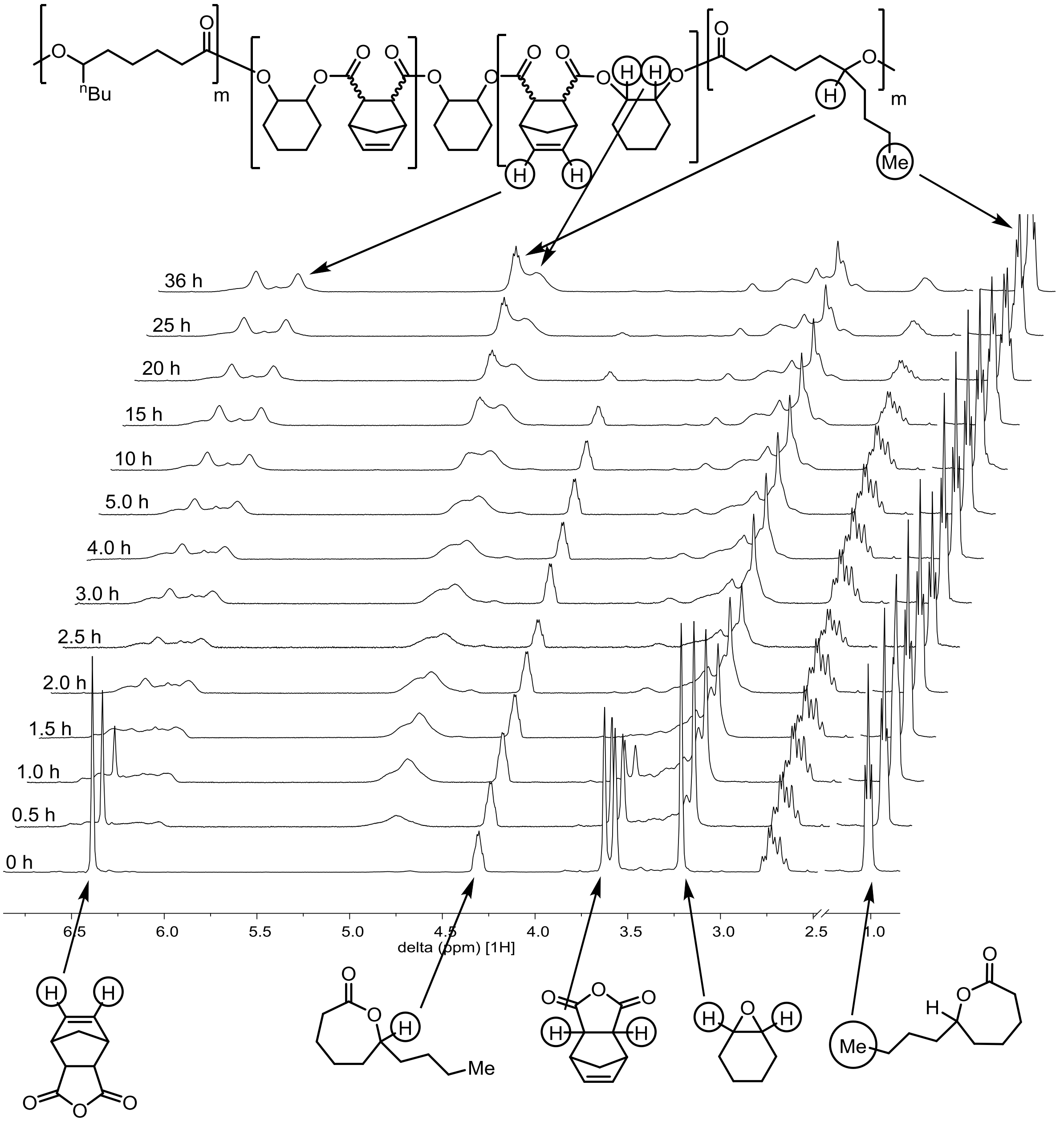

Figure S 6 – ^{1}H NMR spectra for polymerization using NBA/CHO/DL. Polymerization conditions: [SalcyCrCl]:PPNCl:CHD:NBA:CHO:DL = 1:1:10:100:125:100, Tol-d_8 (2.5 M), 100 °C

Table S 2 - - Integrals based on ^{1}H NMR spectra (after normalization with mesitylene, Figure S 6) and calculated conversions used for Figure S 7 and Figure 1. Signals were based on the following shifts: 4.45 (NBA), 4.45-4.92 (Polyester), 4.23 (DL) and 3.10 (CHO) ppm. An error of ± 3% was applied to all plots; the conversion of CHO at the end of the reaction was determined as 74 % (data not shown).

Time (h)	NBA (Integral)	NBA (%)	DL (Integral)	DL (%)	Polyester (Integral)	Polyester (%)
0	531	100	143	97.9	0	0
0.5	320	60.2	146	100	124	26.4
1	121	22.7	140	95.8	215	45.9
1.5	0	0	142	97.2	275	58.7
2	0	0	137	93.7	281	60.0
2.5	0	0	140	95.8	287	61.3
3	0	0	134	91.7	272	58.1
4	0	0	129	88.2	281	60.0
5	0	0	127	86.8	280	59.8
10	0	0	102	69.6	305	65.1
15	0	0	65	44.1	350	74.7
20	0	0	32	21.3	340	72.6
25	0	0	11	6.8	393	83.9
36	0	0	1	0	468	100

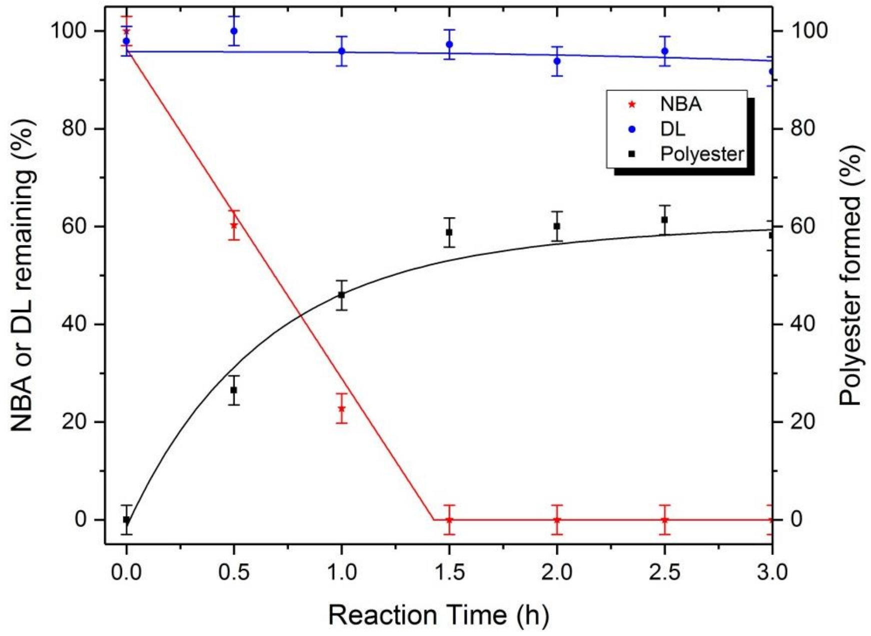

Figure S 7 – Plot showing conversion of NBE and DL over the first 3 hours of the reaction.

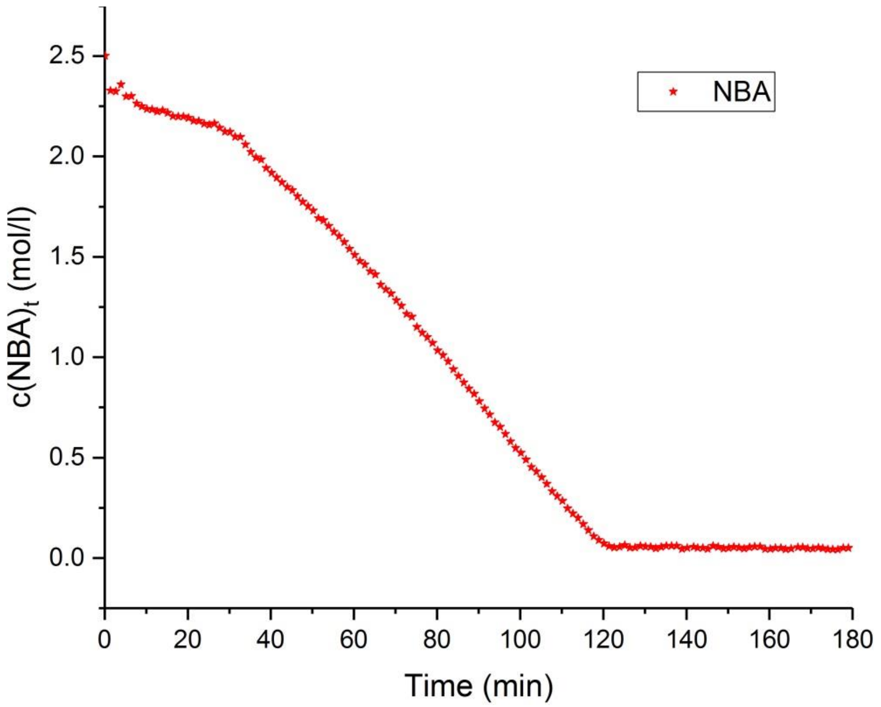

Figure S 8 - *In situ* monitoring of IR band of NBA at 1850 cm^{-1}.

4.3. Reaction of PDL-*b*-PCHNBE-*b*-PDL with Lithium Aluminium Hydride

Cis-5-norbornene-*endo*-2,3-dicarboxylic anhydride (NBA) to *cis*-*endo*-2,3-Bis(hydroxymethyl)bicyclo[2.2.1]hept-5-ene

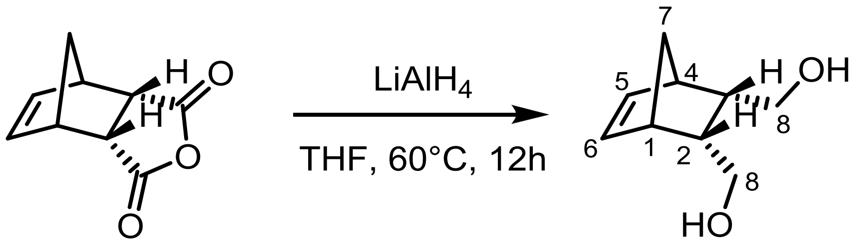

The anhydride was degraded with Lithium aluminium hydride, according to a procedure published by Coates and co-workers.[6] In a glovebox, lithium aluminium hydride (0.50 g, 13.17 mmol) was added to a solution of the polyester (0.32 g) or the anhydride (0.16 g, 0.97 mmol) in dry THF (20 mL) and stirred at room temperature for 5 minutes and then for 24 hours at 60°C. The reaction was then cooled to 0°C and diethyl ether (40 mL), water (0.30 mL) and sodium hydroxide solution (0.30 mL of a 2.0 M solution) was added. After adding additional water (0.6 mL), the solution was dried (magnesium sulfate), filtered and evaporated. ^{1}H NMR ($CDCl_3$, 400 MHz, RT) δ 6.04 (2H, s, $H^{5,6}$), 3.66-3.63 (2H, m, H^8), 3.40 (2H, m, $H^{8'}$), 3.23 (2H, s, OH), 2.80 (2H, s, $H^{1,4}$), 2.54 (2H, m, $H^{2,3}$), 1.40 (2H, m, H^7). ^{1}H NMR shifts in agreement with literature reports.[7]

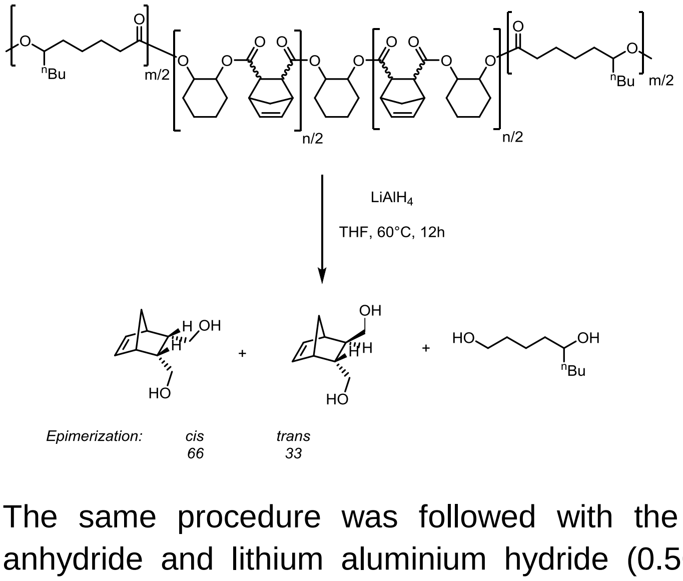

The same procedure was followed with the polyester (0.32 g) instead of the anhydride and lithium aluminium hydride (0.50 g, 13.17 mmol) was added to a solution of the polymer in dry THF (20 mL). After the workup, the alkene region of the ^{1}H NMR of the crude reaction mixture was analysed and the cis:trans ratio was determined as 66:33.

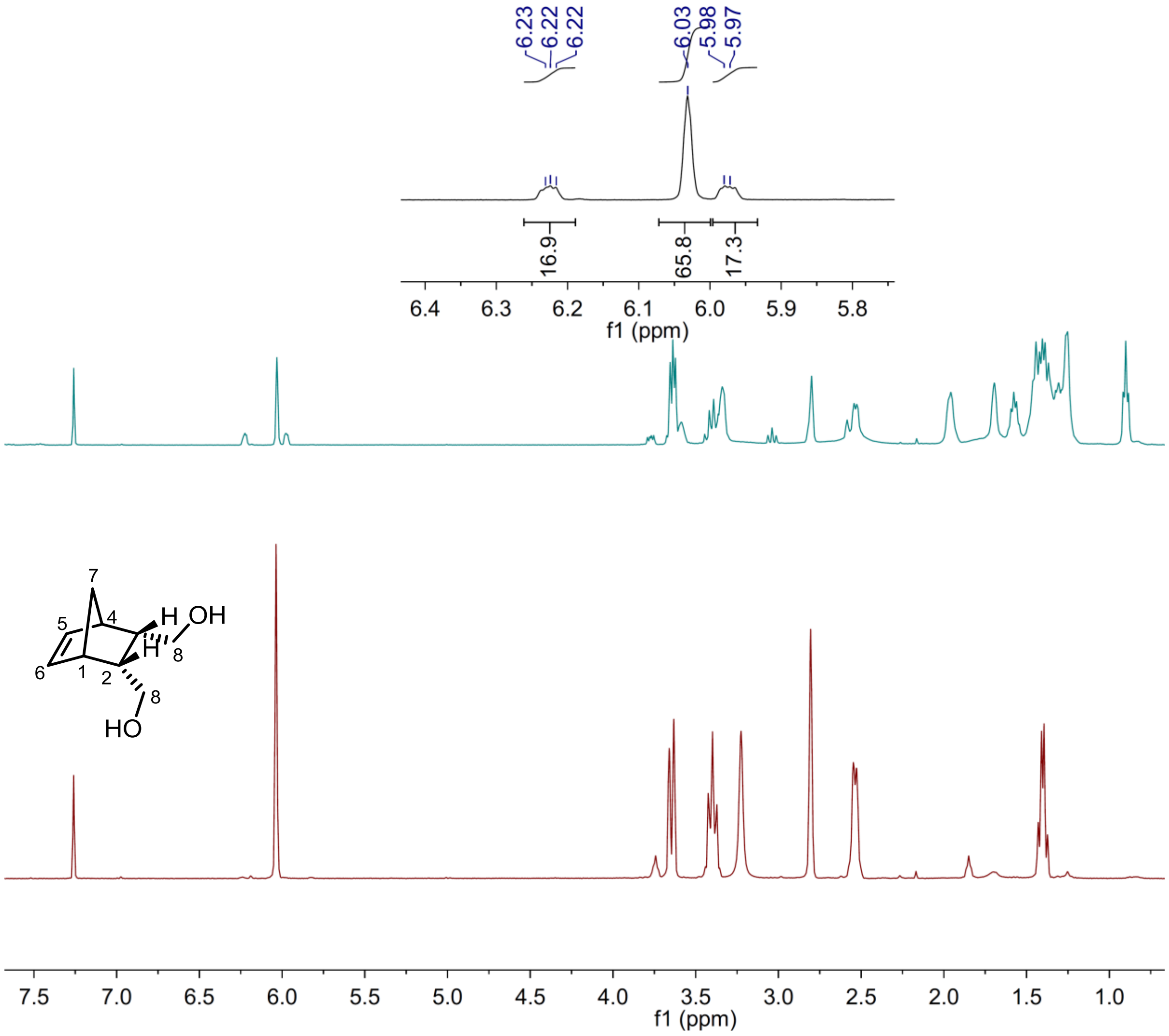

Figure S 9 – Degradation products of polyester (top) and NBA (bottom) with $LiAlH_4$. The relevant alkene section for the polymer-degradation product is enlarged to determine the *cis*:*trans* ratio. The spectra were in agreement with recent work by Coates et al.[8]

4.4. Characterization of PDL-*b*-PCHNBE-*b*-PDL

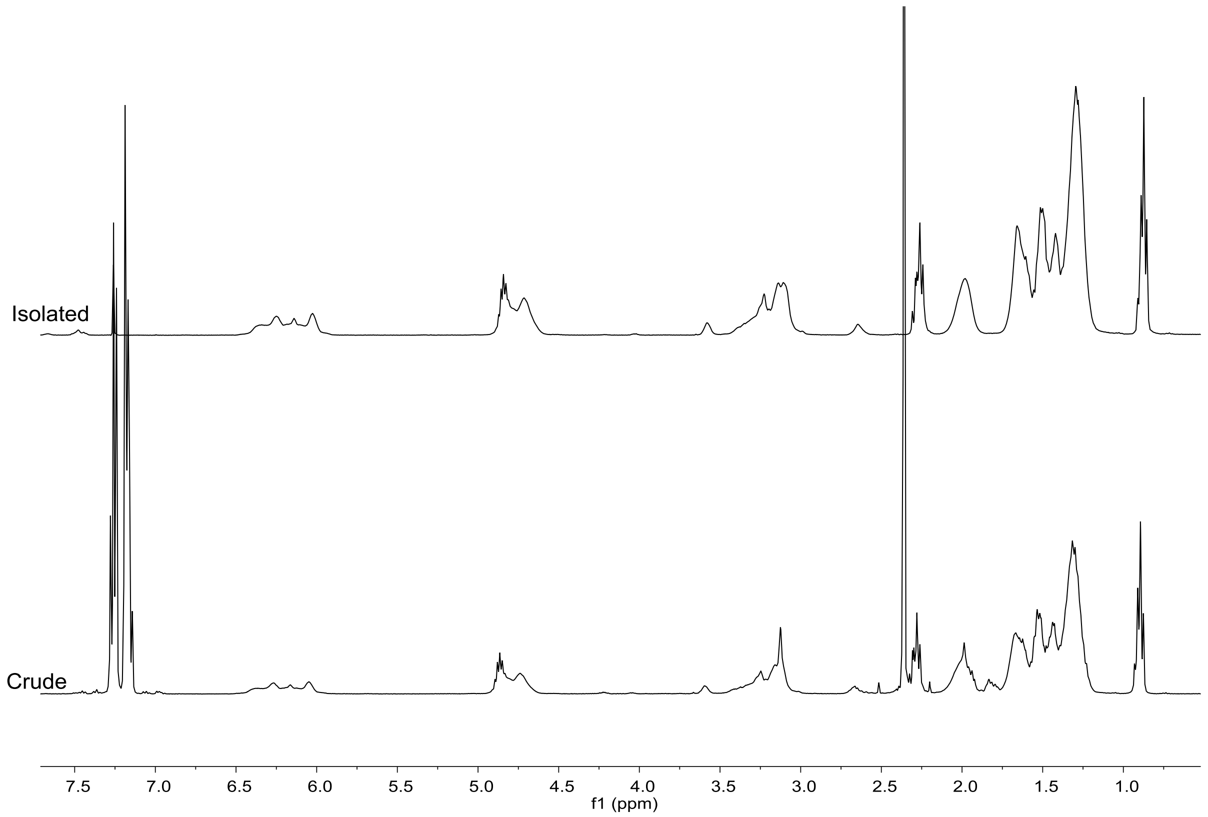

Figure S 10 - ^{1}H NMR spectra of crude (bottom) and isolated (top) polymer based on NBA/CHO/DL.

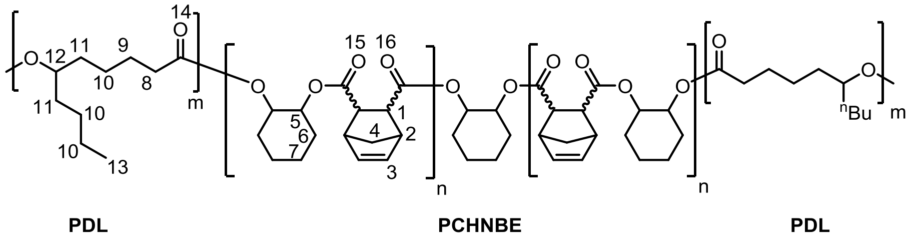

^{1}H NMR (500 MHz, $CDCl_3$) δ 6.36-6.03 (m, 1.00 H, H^3), 4.84-4.71 (m, 1.35 H, $H^{5,12}$), 3.58 (m, 0.12 H, $H^{12\text{-Endgrp}}$), 3.37-3.11 (m, 1.80 H, $H^{1,2}$), 2.25 (m, 0.79 H, H^8), 1.98 (m, 1.09 H, H^6), 1.70 – 1.56 (m, 2.01 H, $H^{7,9}$), 1.55 – 1.45 (m, 1.54 H, H^{11}), 1.46 – 1.38 (m, 1.01 H, $H^{4,11\text{-Endgrp}}$), 1.38 - 1.15 (m, 4.44 H, $H^{4',6',7',10}$), 0.87 (m, 1.22 H, H^{13}).

^{13}C NMR (126 MHz, $CDCl_3$) δ 173.61-171.27 ($C^{14,15,16}$)137.72-133.52 (C^3), 73.98 (C^5), 73.83-72.60 (m, C^{11}), 71.76 (s, $C^{12\text{-Endgrp}}$), 48.65-48.00 (C^4), 47.06-45.68 ($C^{8/1\text{Junction},1,2}$), 37.34-37.20 ($C^{11\text{-Endgrp}}$), 34.70-34.45 ($C^8$), 33.92-33.85 ($C^{11}$), 30.02 ($C^6$), 27.97-27.60 ($C^{10}$), 25.37-23.40 ($C^{7,9}$), 22.87-22.69 ($C^{10}$), 14.21 ($C^{13}$).

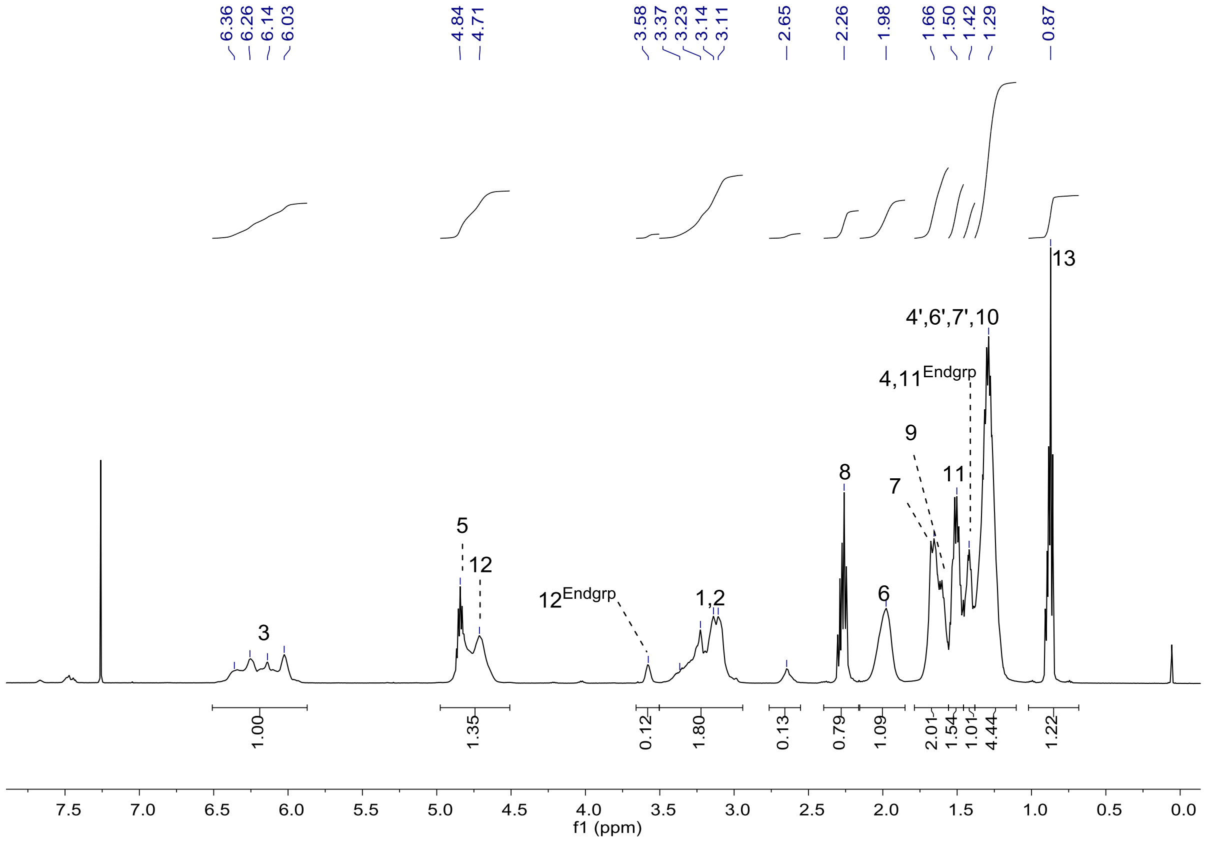

Figure S 11 – ^{1}H NMR spectra of isolated polymer based on NBA/CHO/DL. Traces of PPNCl are observed at 7.5 ppm.

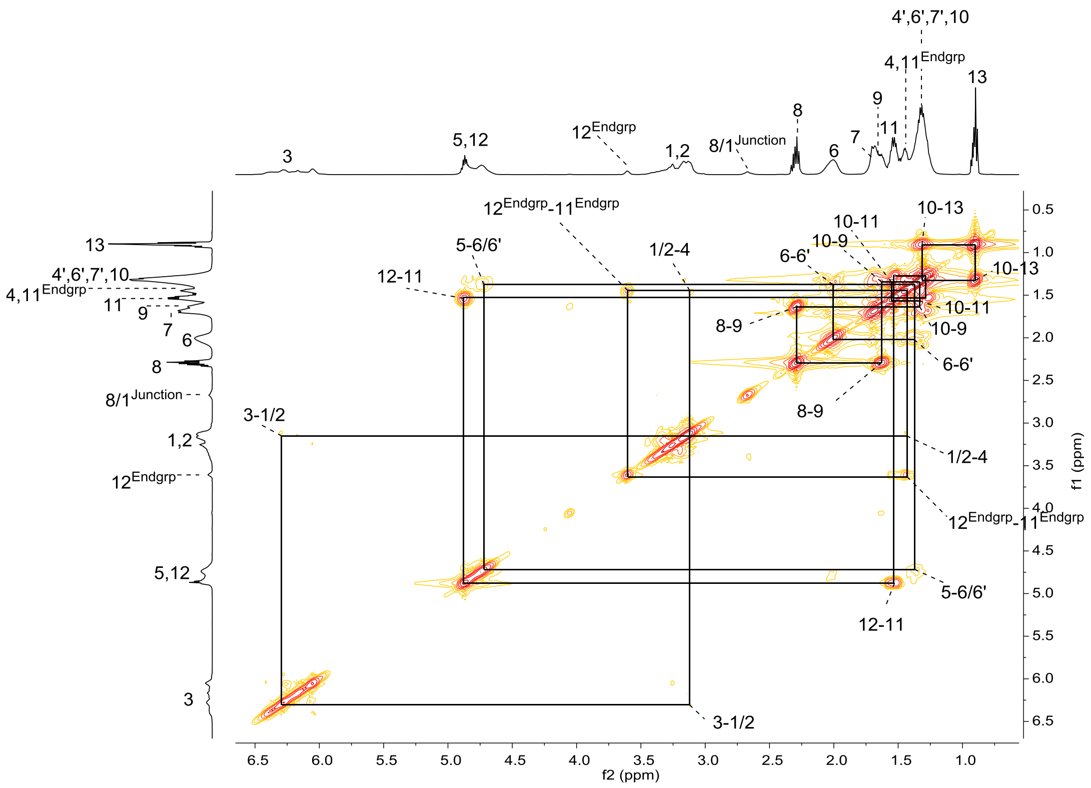

Figure S 12 - COSY NMR spectrum of isolated polymer based on NBA/CHO/DL.

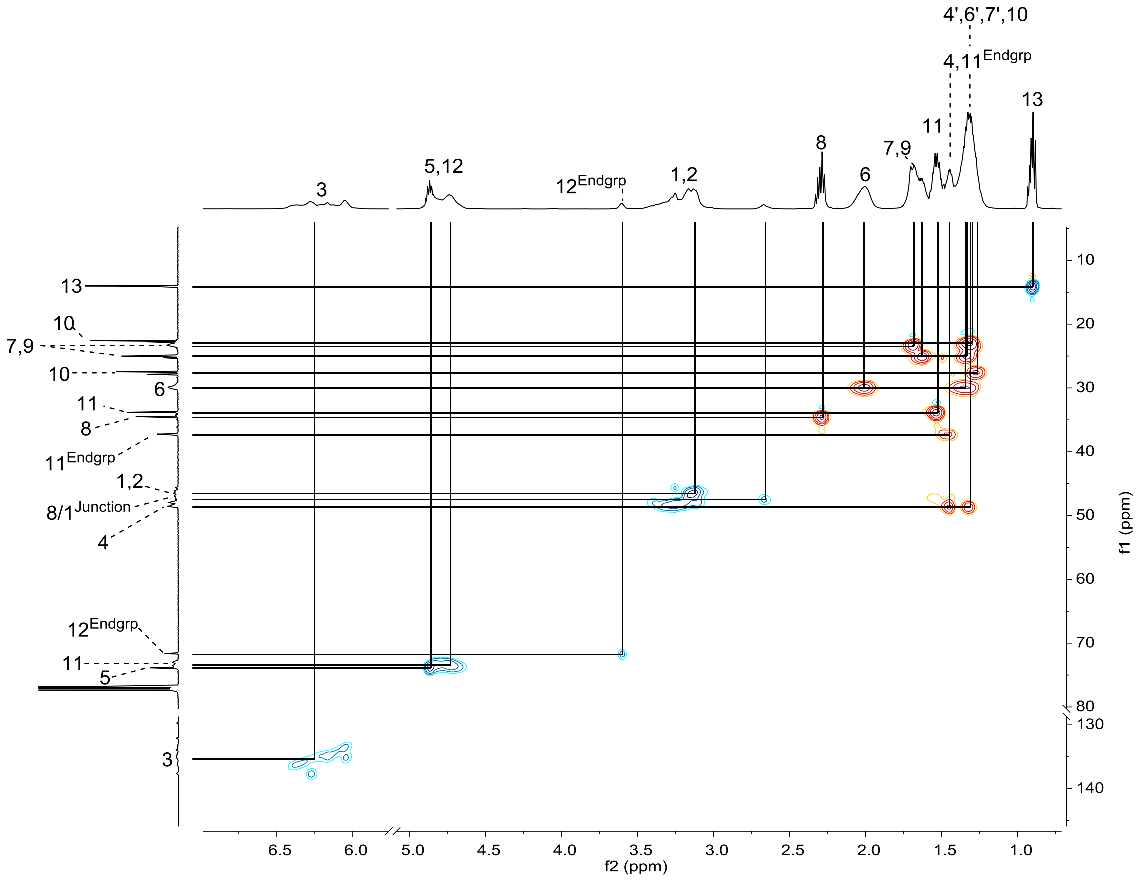

Figure S 13 - HSQC NMR spectrum of isolated polymer based on NBA/CHO/DL.

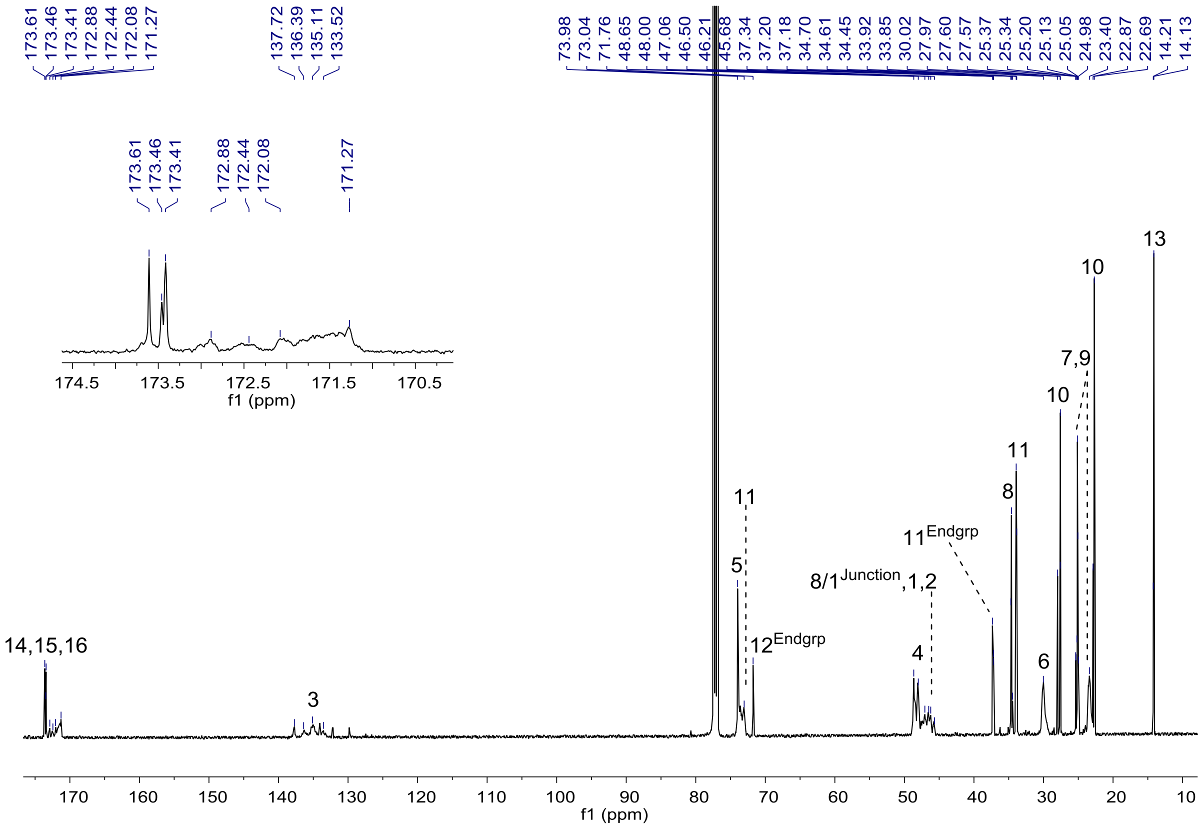

Figure S 14 - $^{13}C\{^{1}H\}$ NMR spectrum of isolated polymer based on NBA/CHO/DL. Traces of PPNCl are observed at 129.8, 132.2 and 134.1 ppm.

It is proposed that the $^{13}C\{^{1}H\}$ NMR spectrum was complicated by the a mixture of cis and trans-ester linkages (66:34; likely to be distributed randomly throughout the polymer backbone), as illustrated below:

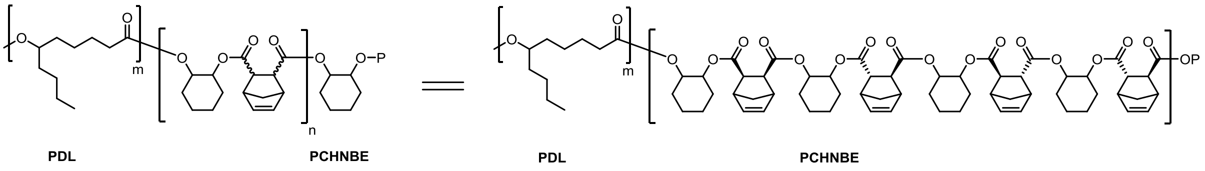

4.5. Transesterification with DBU

The polyester (30 mg), DBU (1.0 mg) and toluene-d_8 (0.5 mL) were added to a J Young NMR tube in the glovebox and left to react under polymerization conditions (100 °C, 3 days). The $^{13}C\{^1H\}$ NMR spectra of the original polymer and the polymer exposed to DBU were compared (Figure S 15).

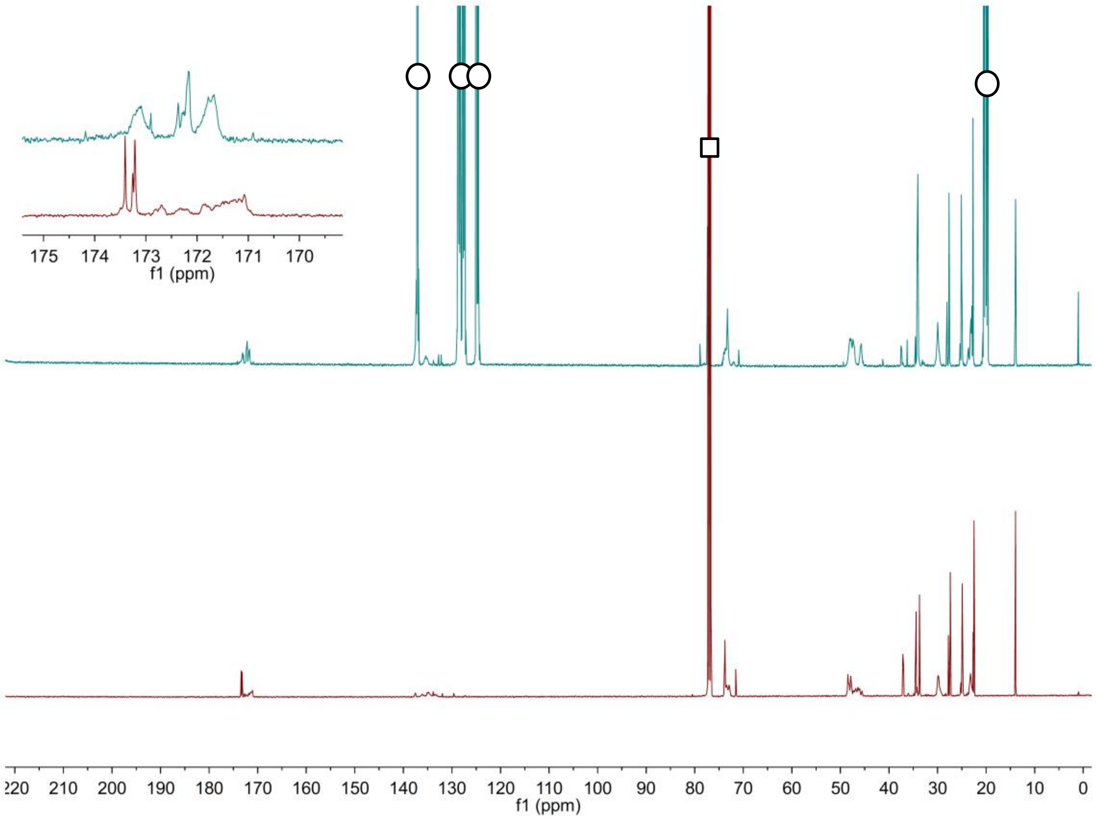

Figure S 15 – $^{13}C\{^1H\}$ NMR before (bottom, in $CDCl_3$) and after (top, in Tol-d_8) transesterification with DBU. Residual toluene is marked with a sphere; $CDCl_3$ is marked with a square. The characteristic region for carbonyl resonances is enlarged.

4.6. DOSY NMR Spectra of PDL-*b*-PCHNBE-*b*-PDL

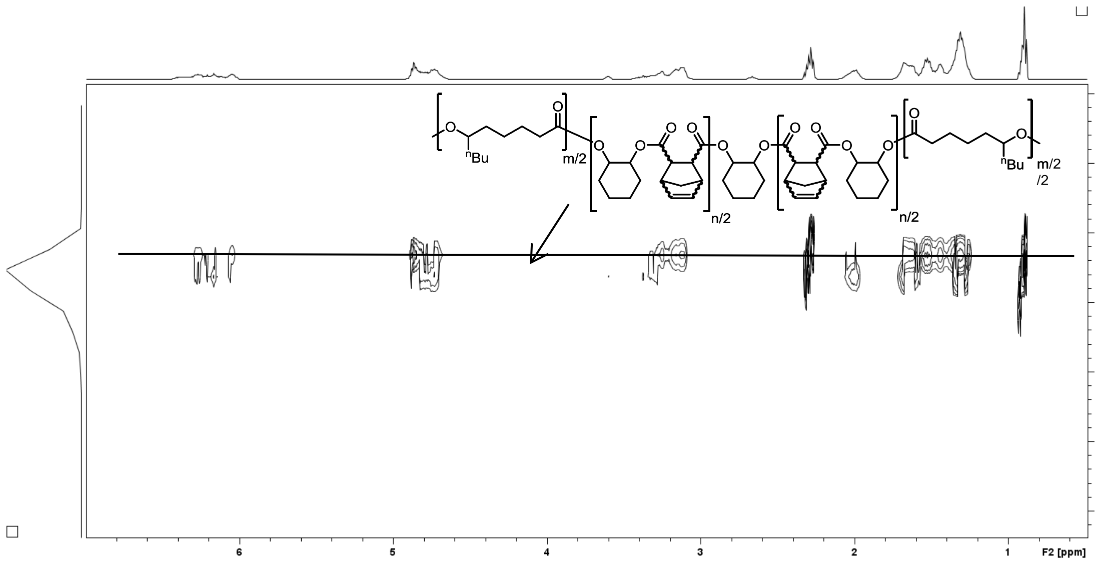

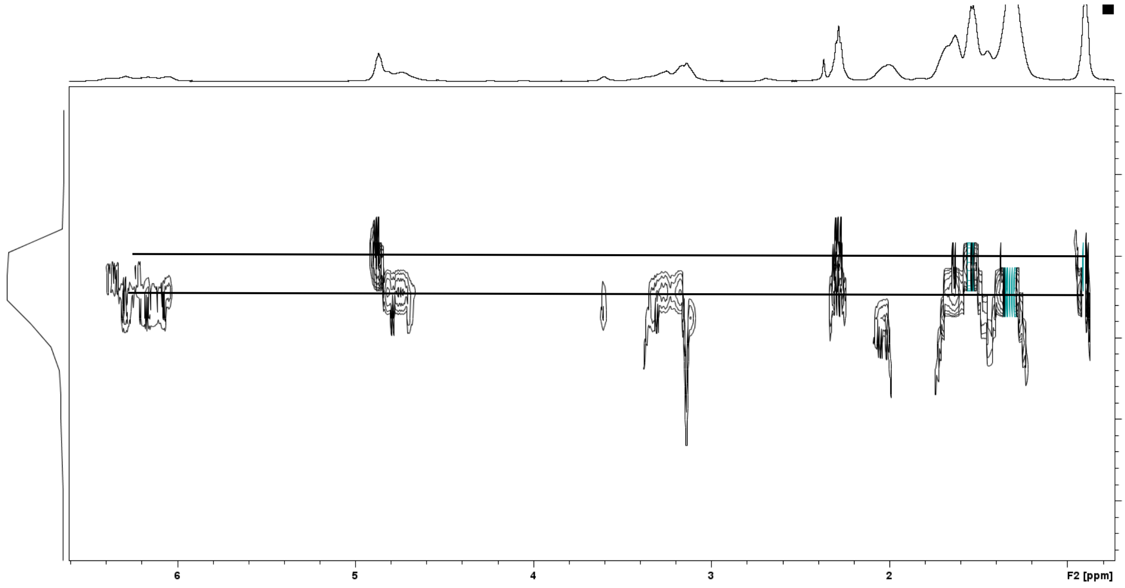

Figure S 16 - DOSY NMR spectra of PDL-*b*-PCHNBE-*b*-PDL (top) and mixture of homopolymers of similar chain length (bottom) in $CDCl_3$.

4.7. End-group Assay with ^{31}P{^{1}H} NMR spectroscopy

The procedure described in the Materials section ('End-group assay for hydroxyl groups with 2-chloro-4,4,5,5-tetramethyl dioxaphospholane', p. S 3) was followed with three polymers, PDL, PCHNBE and the polymer based on 'Switch' catalysis. The ^{31}P{^{1}H} NMR chemical shifts are given in Table S 3, the spectra are shown in Fig. S 18.

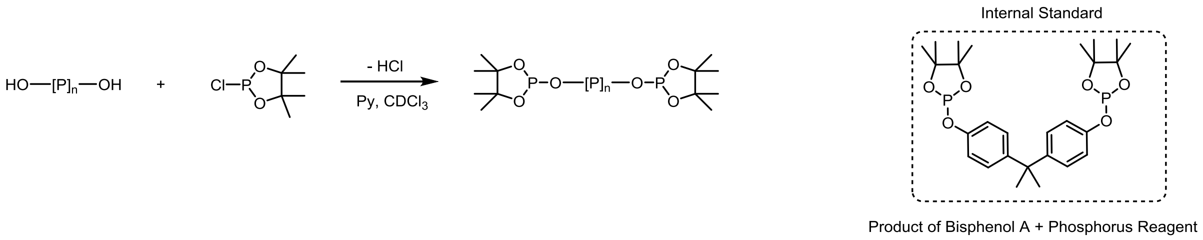

Table S 3 - ^{31}P{^{1}H} NMR chemical shifts after reaction with 2-chloro-4,4,5,5-tetramethyl dioxaphospholane for different polymers.

Polymer	Chemical shift after reaction with Phosphorus Reagent
PDL	147.1
PCHNBE	146.5
PDL-*b*-PCHNBE-*b*-PDL	147.1 (87%) + 146.5 (13%)

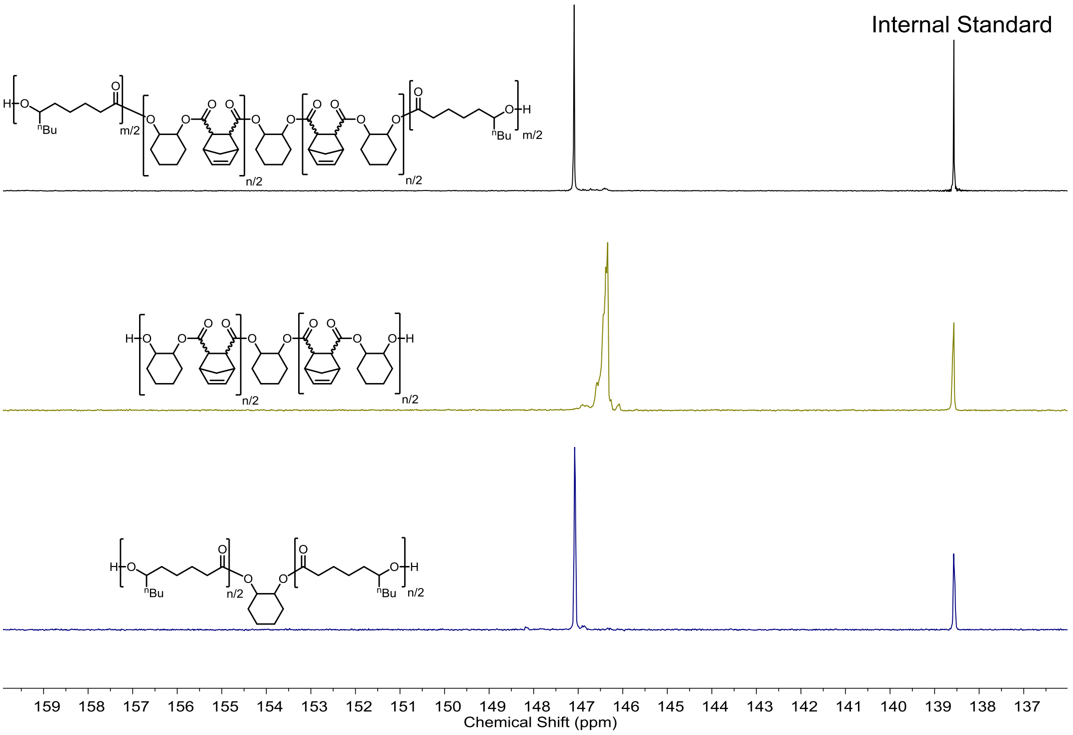

Figure S 17 – ^{13}P {^{1}H} NMR spectra after reacting different polymers with 2-chloro-4,4,5,5-tetramethyl dioxaphospholane. From top to bottom: the polymer obtained in 'Switch' catalysis (PDL-*b*-PCHNBE-*b*-PDL), PCHNBE and PDL.

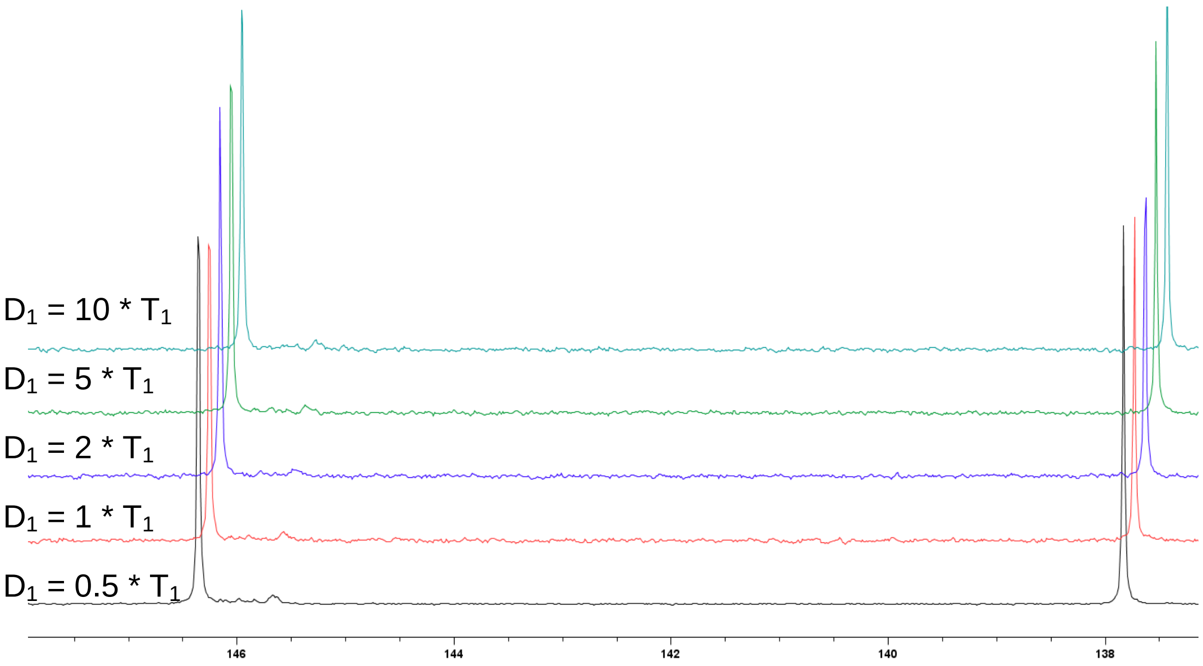

Figure S 18 - ^{13}P {^{1}H} NMR spectra after reacting (PDL-*b*-PCHNBE-*b*-PDL) with 2-chloro-4,4,5,5-tetramethyl dioxaphospholane at various recycle delays (D_1) to account for the different relaxation times (T_1) of the species shown in Table S 3. The presence of only one peak at 147.2 ppm should be noted at all relaxation times (indicating PDL endgroups and the abesnce of PCHNBE endgroups).

4.9. Polymerization of NBA,CHO and DL with an excess of NBA

The general polymerization procedure was followed with 125 equiv. of CHO, 200 equiv. of NBA and 200 equiv. of DL. After four days, no significant conversion of DL was observed (< 5%). CHO (200 equiv.) was then added under inert conditions and the mixture was allowed to react for 3 days. The final conversion of DL and NBA were determined 58% and 99%, respectively. The GPC chromatograms and ^{1}H NMR spectra are shown before and after the addition of CHO in

Figure S 21 and Figure S 20.

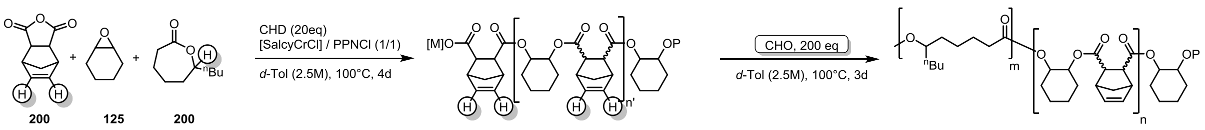

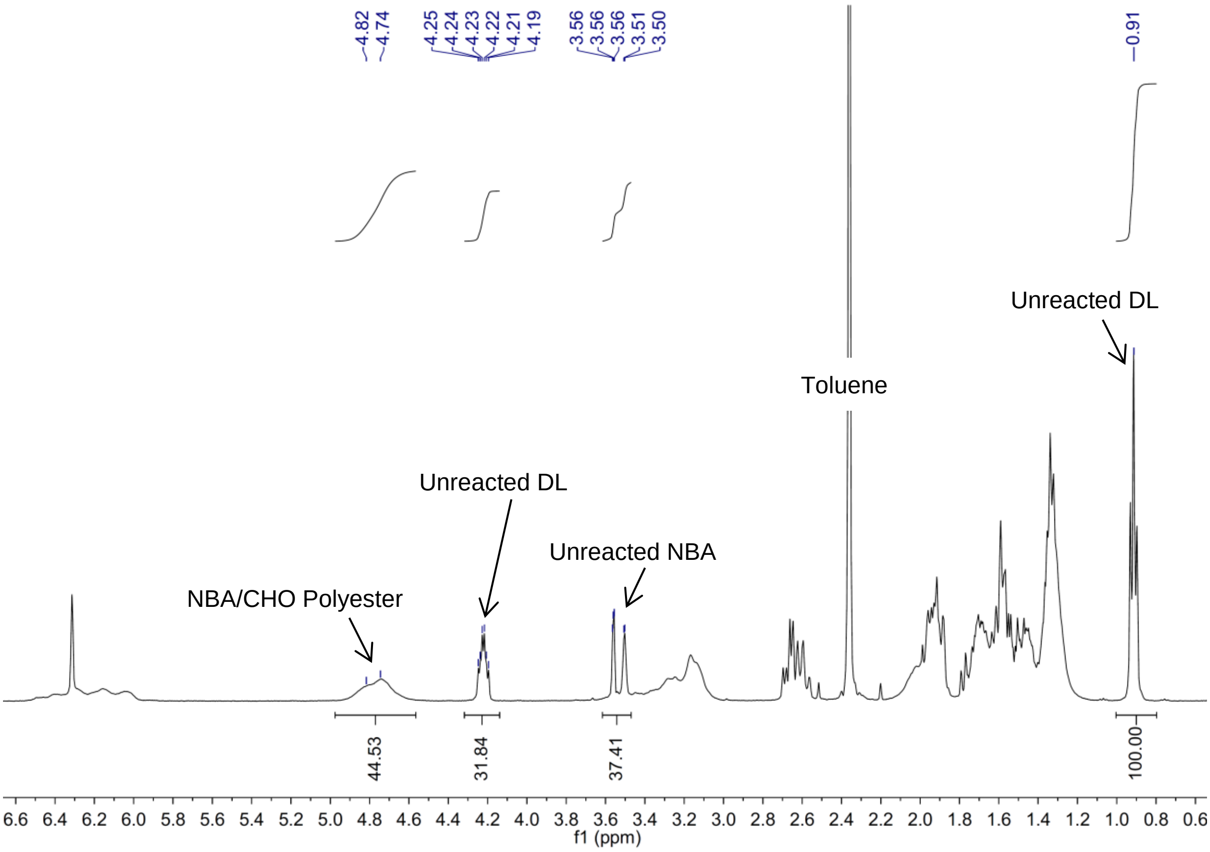

Figure S 19 – ^{1}H NMR spectrum after four days with an excess of anhydride.

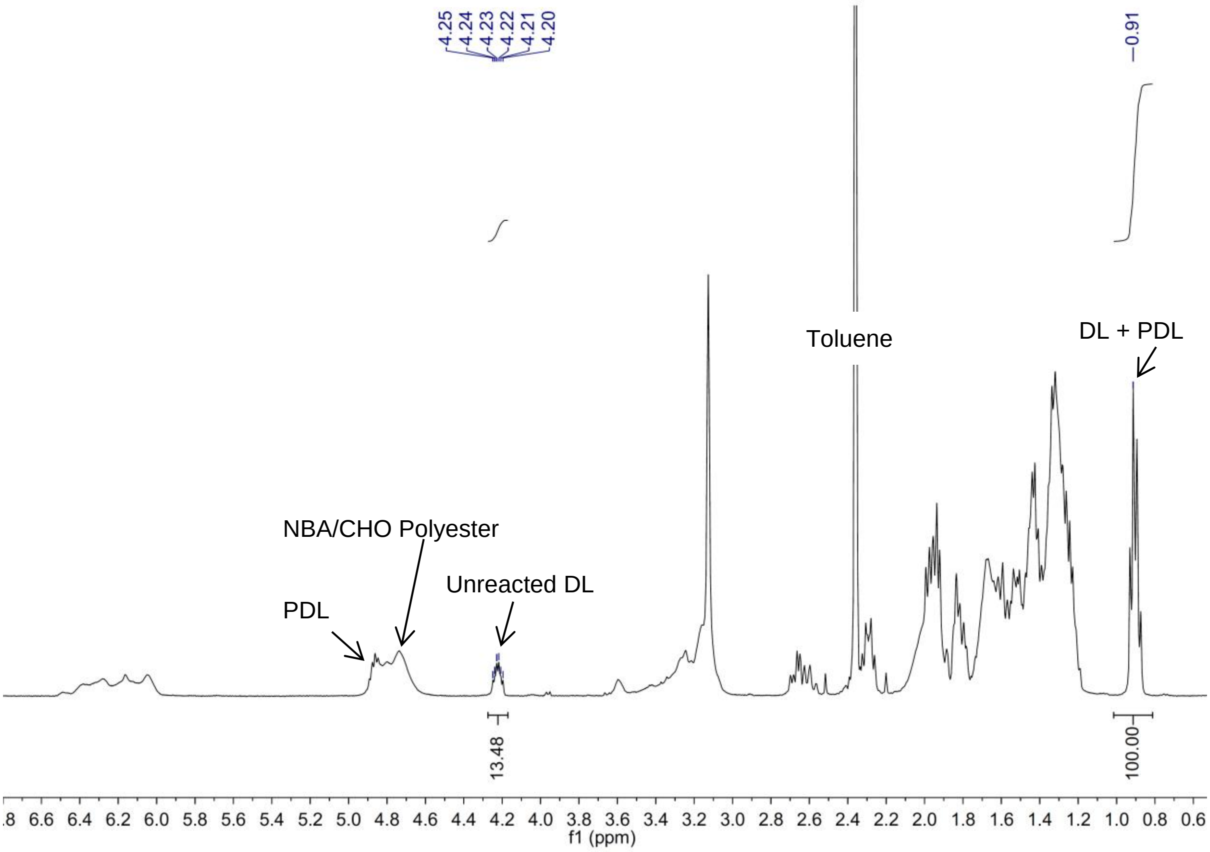

Figure S 20 - ^{1}H NMR spectrum after the addition of CHO.

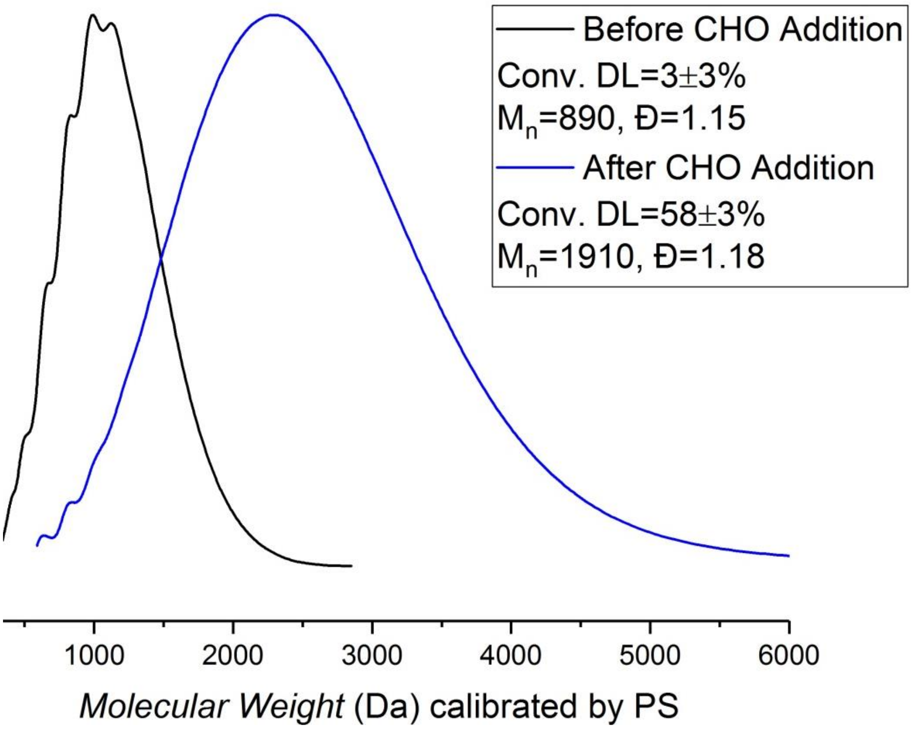

Figure S 21 – GPC chromatograms before and after the addition of CHO.

4.10. Thermal Analysis of polyesters with different PCHNBE:PDL block ratios

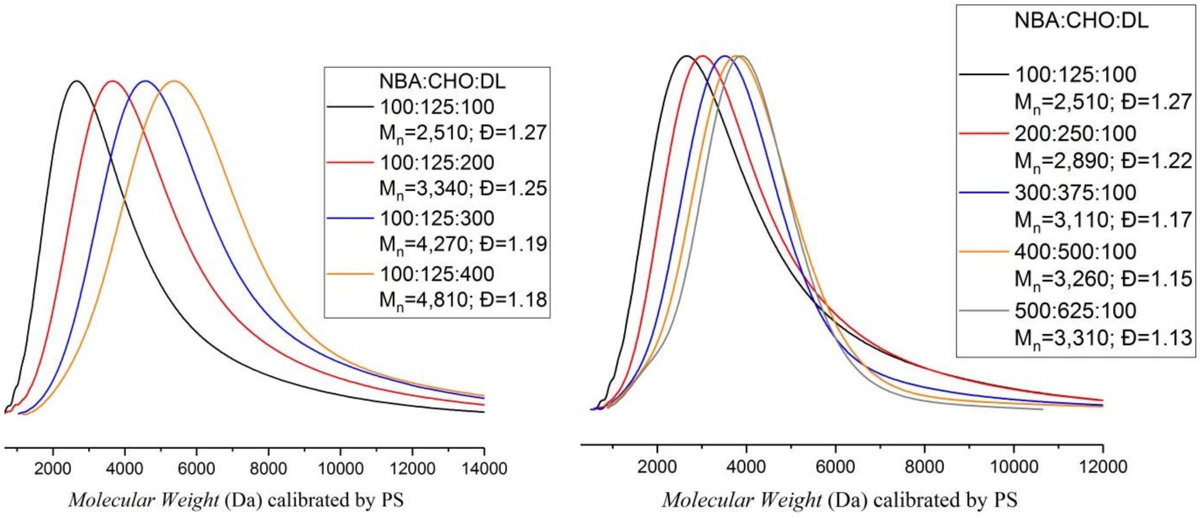

Figure S 22 - GPC chromatograms for block polyesters based on NBA,CHO, DL for different block ratios.

Table S 4 - Block polyesters with variable compositions and properties.

NBA:CHO:DL[a]	PCHNBE:PDL[b]	wt% PCHNBE: wt% PDL[c]	M_n (PDI)[c]	T_g (°C)[d]	$T_{g,calc}$ (°C)[e]
100:125:400	1:4	27:73	4,810 (1.18)	-30	-28
100:125:300	1:3	34:66	4,270 (1.19)	-21	-21
100:125:200	1:2	44:56	3,340 (1.25)	-4	-10
100:125:100	1:1	61:39	2,510 (1.27)	26	13
200:250:100	2:1	76:24	2,890 (1.22)	57	36
300:375:100	3:1	82:18	3,110 (1.17)	71	46
400:500:100[f]	5:1	89:11	3,260 (1.15)	84	60
500:625:100[g]	16:1	96:4	3,310 (1.13)	111	73

[a] Molar ratio of starting materials; generally both processes proceed to >95% monomer conversion (Fig. S22). [b] Molar ratio of the polyester blocks based on conversion of starting materials (Fig. S22). [c] Weight fractions of polyester blocks; calculated from molar ratios. [c] Determined by GPC, THF, 30 °C, using polystyrene standards to calibrate the instrument (Fig. S21). [d] Determined by DSC measurements from -90 – 160 °C at 10°C/min and taken from the third heating cycle (Figs. S23). [e] Based on Fox Equation with T_g(PCHNBE)=82°C and T_g(PDL)=-54°C. [f] ~80% DL conversion. [g] ~30% DL conversion.

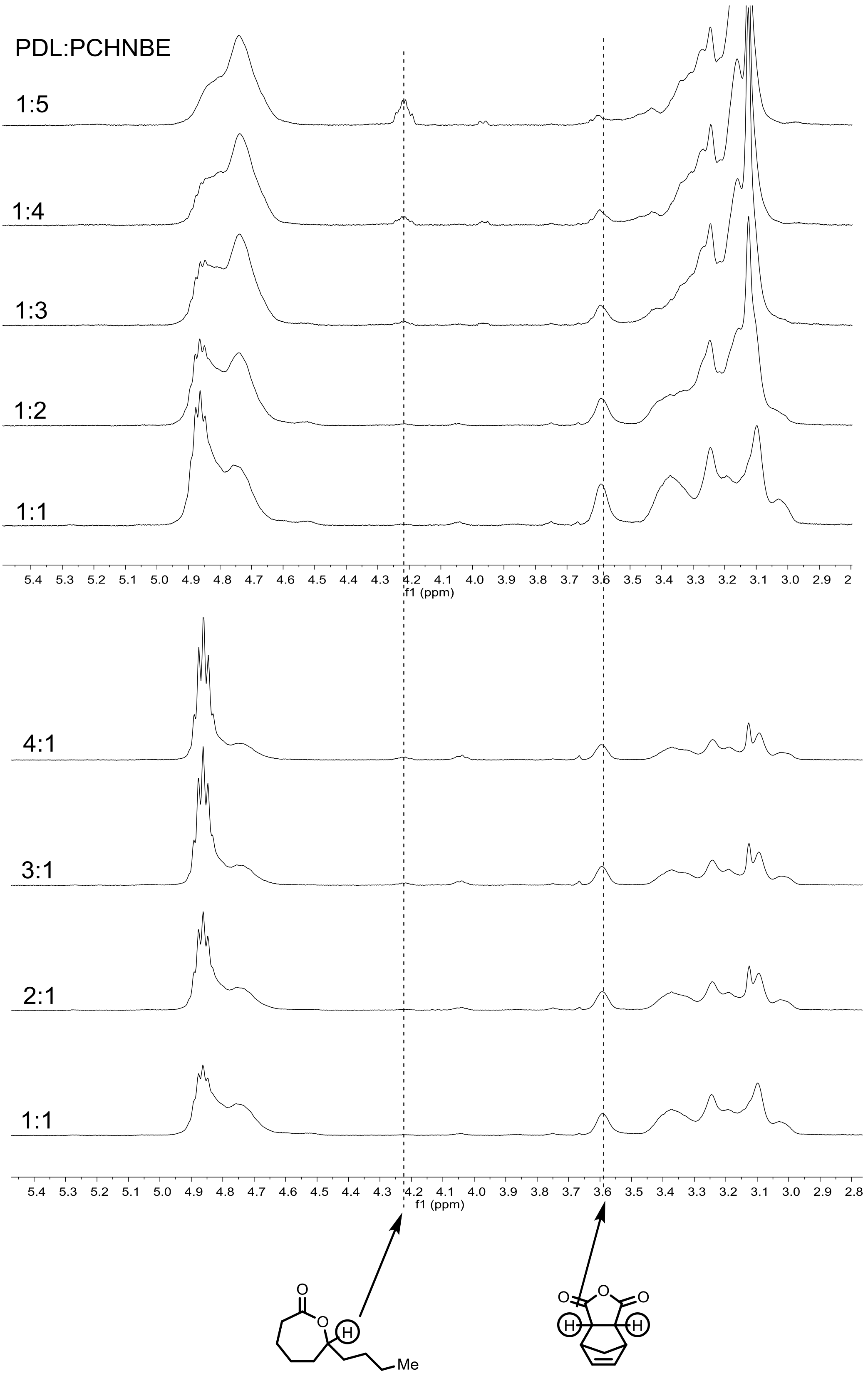

Figure S 23 – ^{1}H NMR spectra of crude block copolymers based on different NBA:CHO:DL ratios. It should be noted that conversions of 99% were obtained in all cases apart from PDL:PCHNBE = 1:5 or 1:4 (DL conversions were 80% and 30%, respectively).

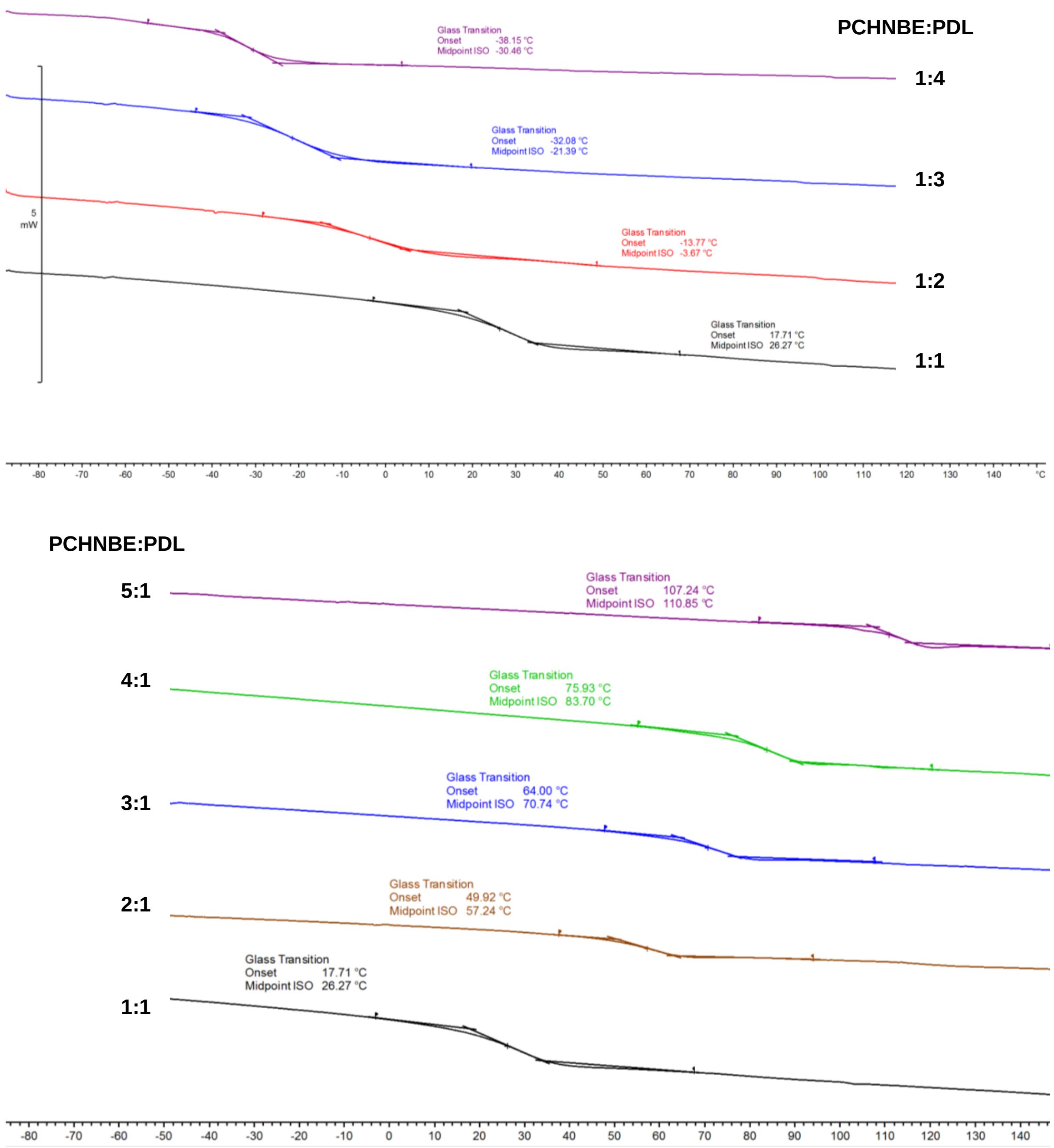

Figure S 24 - DSC thermograms for block polyester based on NBA/CHO/DL at different compositions (see Table S4).

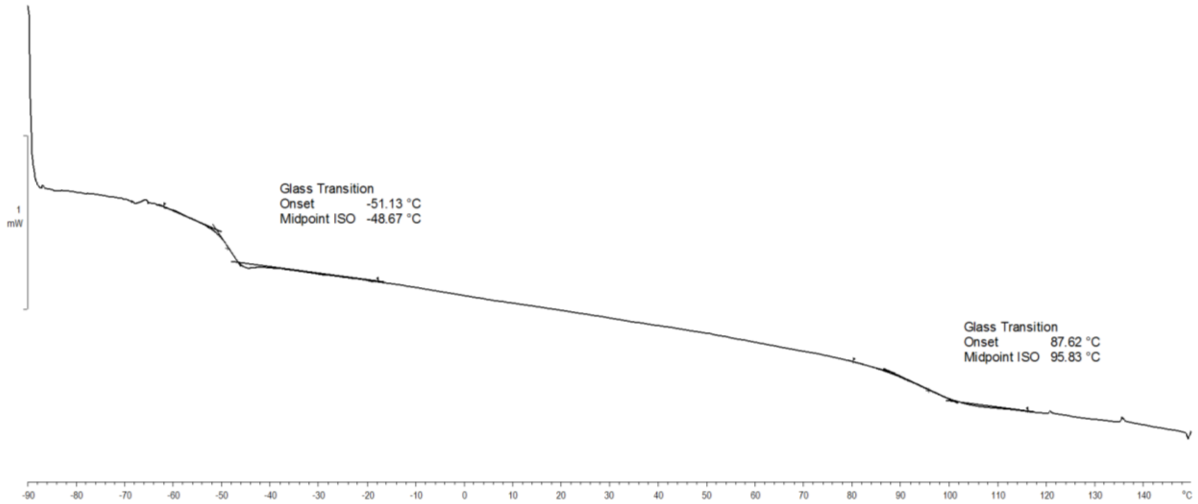

Figure S 25 - DSC thermograms for mixture of homopolymers (molecular weight for PDL: M_n=4,880 g/mol (Đ=1.12); for PCHNBE: M_n=5,330 g/mol (Đ=1.35)).

5. Anhydride Scope

5.1. PA/CHO/DL

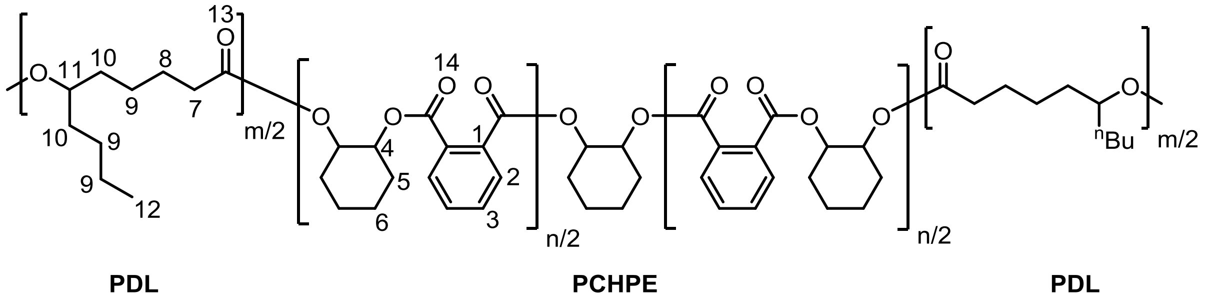

^{1}H NMR (500 MHz, $CDCl_3$) δ 7.59-7.40 (m, 1.00 H, ^{H2,3}), 5.14 (m, 0.44 H, H^4), 4.85 (m, 0.24 H, H^{11}), 3.58 (m, 0.04H, $H^{11\text{-Endgroup}}$), 2.27 (m, 0.92 H, $H^{7,5'}$), 1.76-1.63 (m, 0.52 H, $H^{6,8}$), 1.51-1.28 (m, 1.94 H, $^{H5,10,6',9}$), 0.88 (m, 0.67 H, H^{12}).

^{13}C NMR (126 MHz, $CDCl_3$) δ 173.51-172.98 (C^{13}), 166.68 (C^{14}), 132.04 (C^1), 131.07 (C^2), 128.79 (C^3), 74.77-73.89 ($C^{4,4\text{-Junction*}}$), 73.32 (C^{11}), 71.65 ($C^{11\text{-Endgrp*}}$), 37.23-37.07 ($C^{10\text{-Endgrp}}$), 34.50 (C^7), 33.81-33.74 (C^{10}), 29.91($C^{5,9\text{-Endgrp}}$), 27.86-27.46 (C^9), 25.01 (C^8), 23.41 (C^6), 22.76-22.58 (C^9), 14.10-14.02 (C^{12}).

* Cannot be distinguished from hydroxyl-terminated PCHPE-chains (in which case $C^{4\text{-Junction}}$ would be $C^{4\text{-Esterlinkage}}$ and $C^{11\text{-Endgrp}}$ would be $C^{4\text{-Hydroxyl}}$).

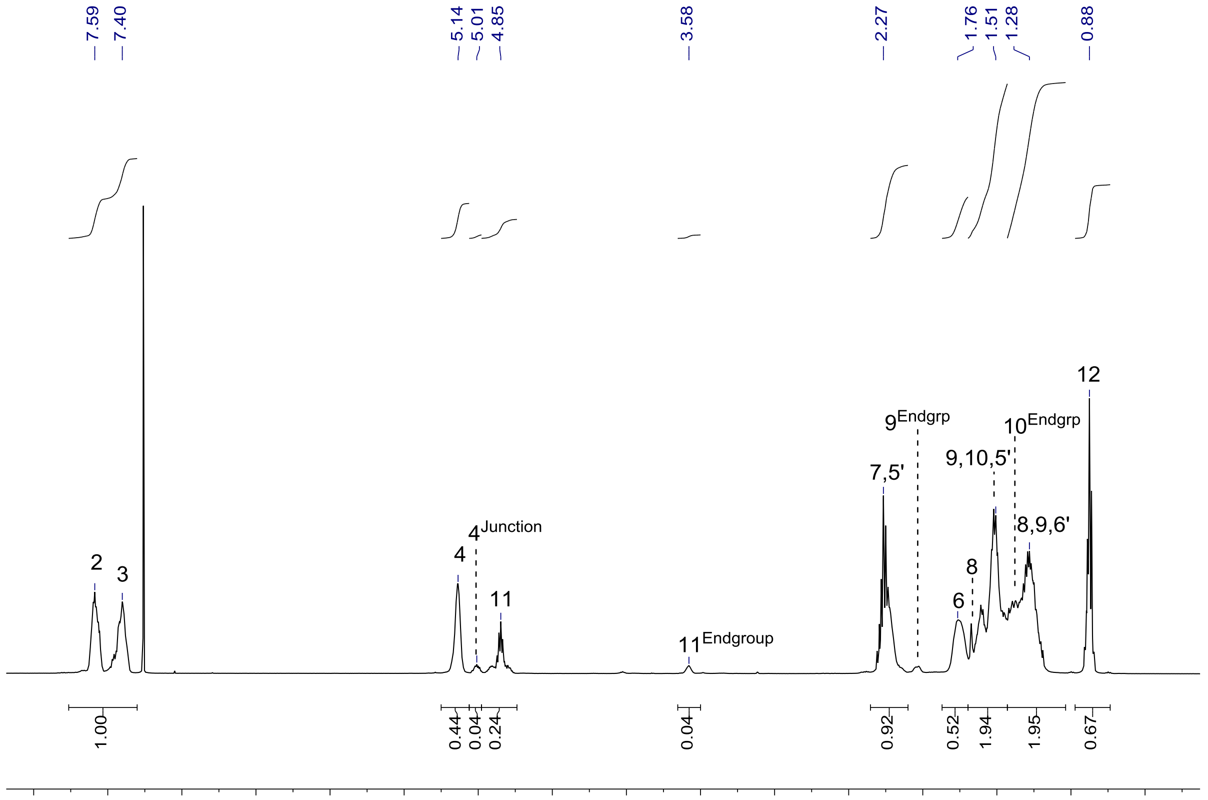

Figure S 26 – ^{1}H NMR spectrum of polymer based on PA, CHO, DL.

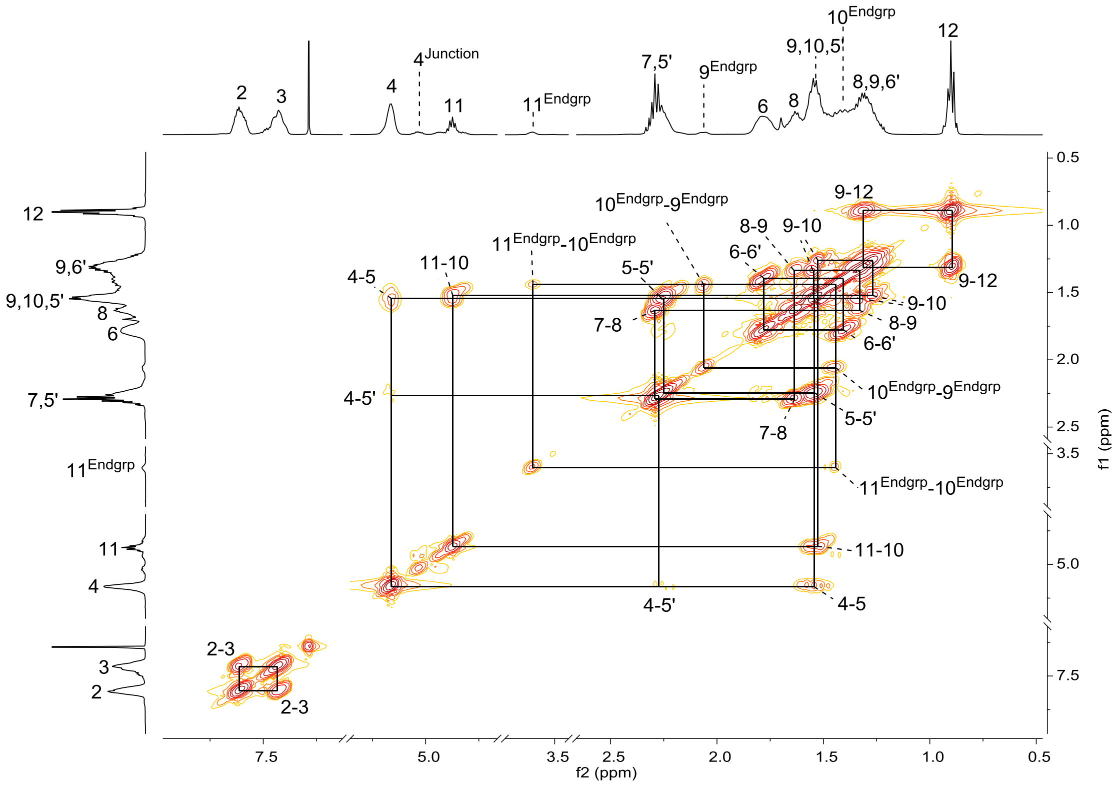

Figure S 27 - COSY NMR spectrum of polymer based on PA, CHO, DL.

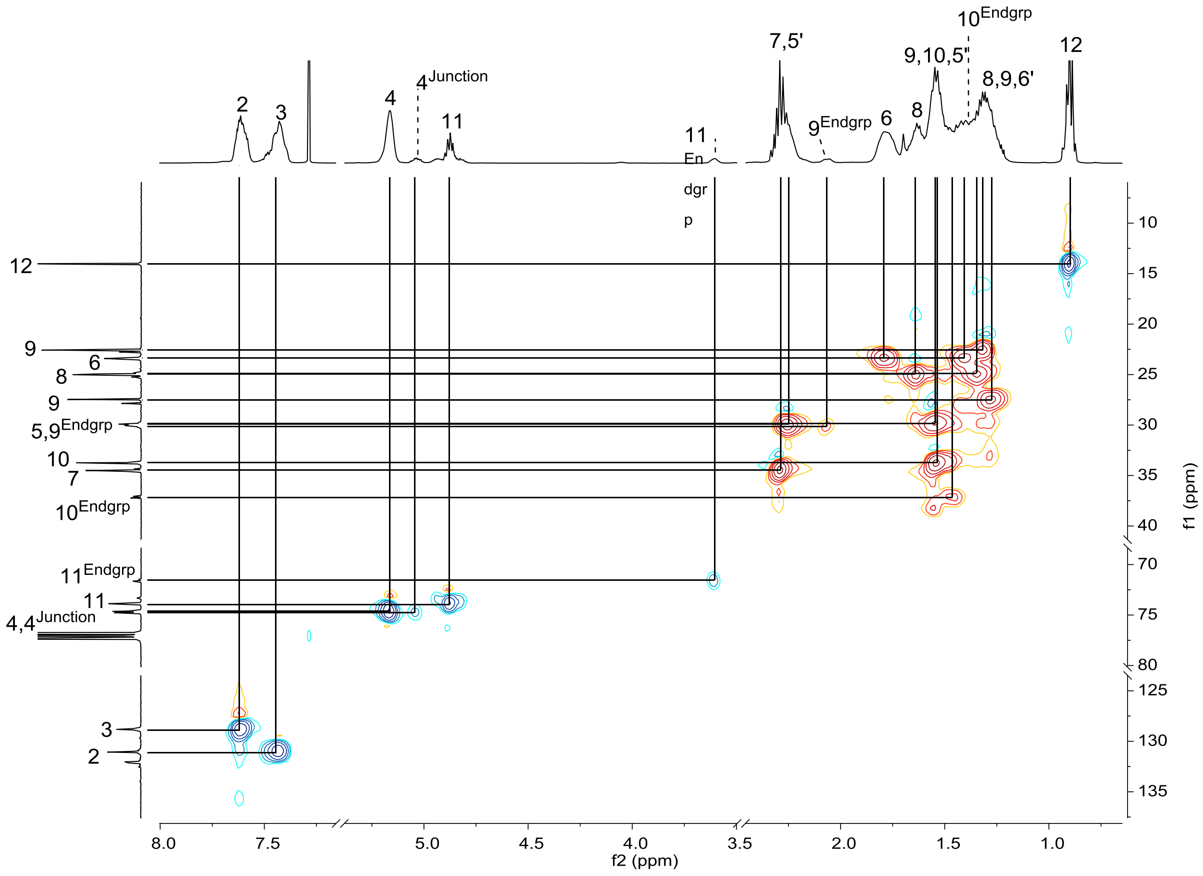

Figure S 28 - HSQC NMR spectrum of polymer based on PA, CHO, DL.

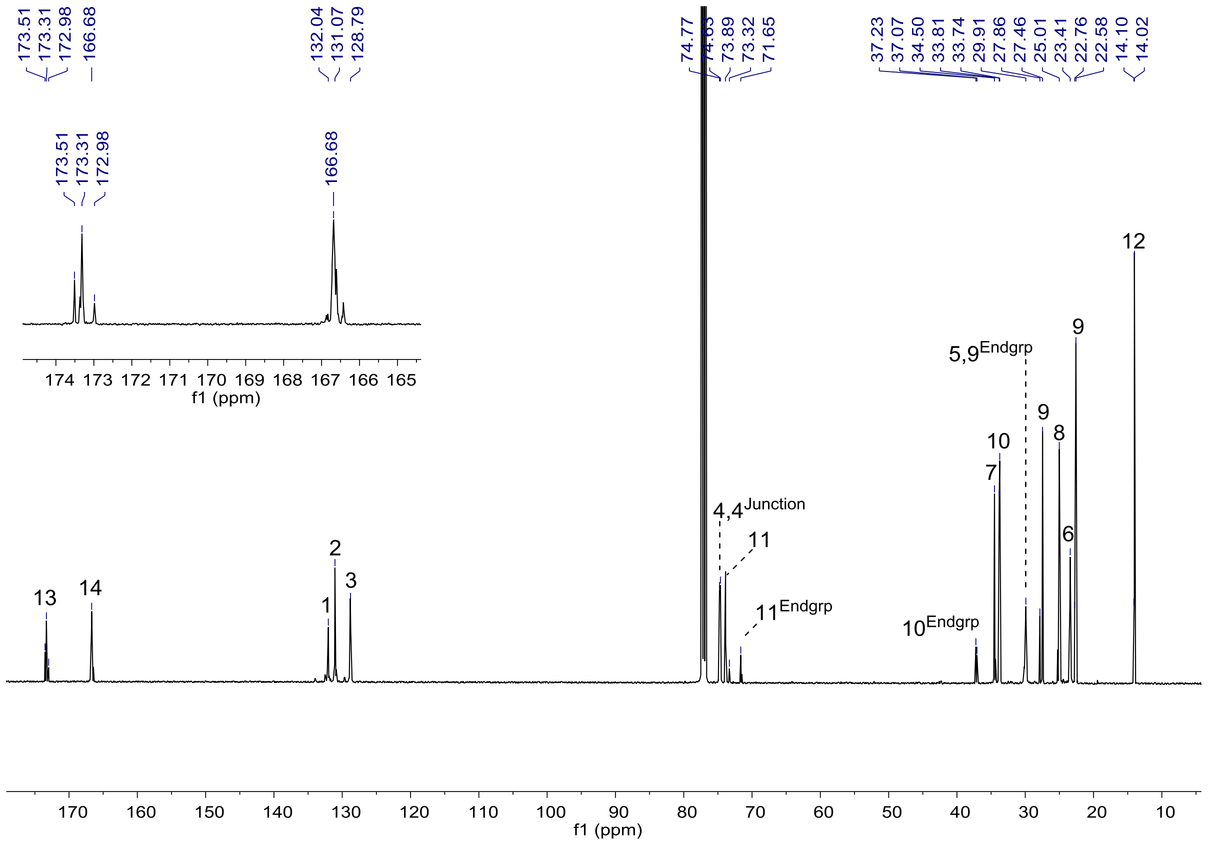

Figure S 29 – $^{13}C\{^{1}H\}$ NMR spectrum of polymer based on PA, CHO, DL. Traces of PPNCl were observed at 129.8, 132.2 and 134.1 ppm.

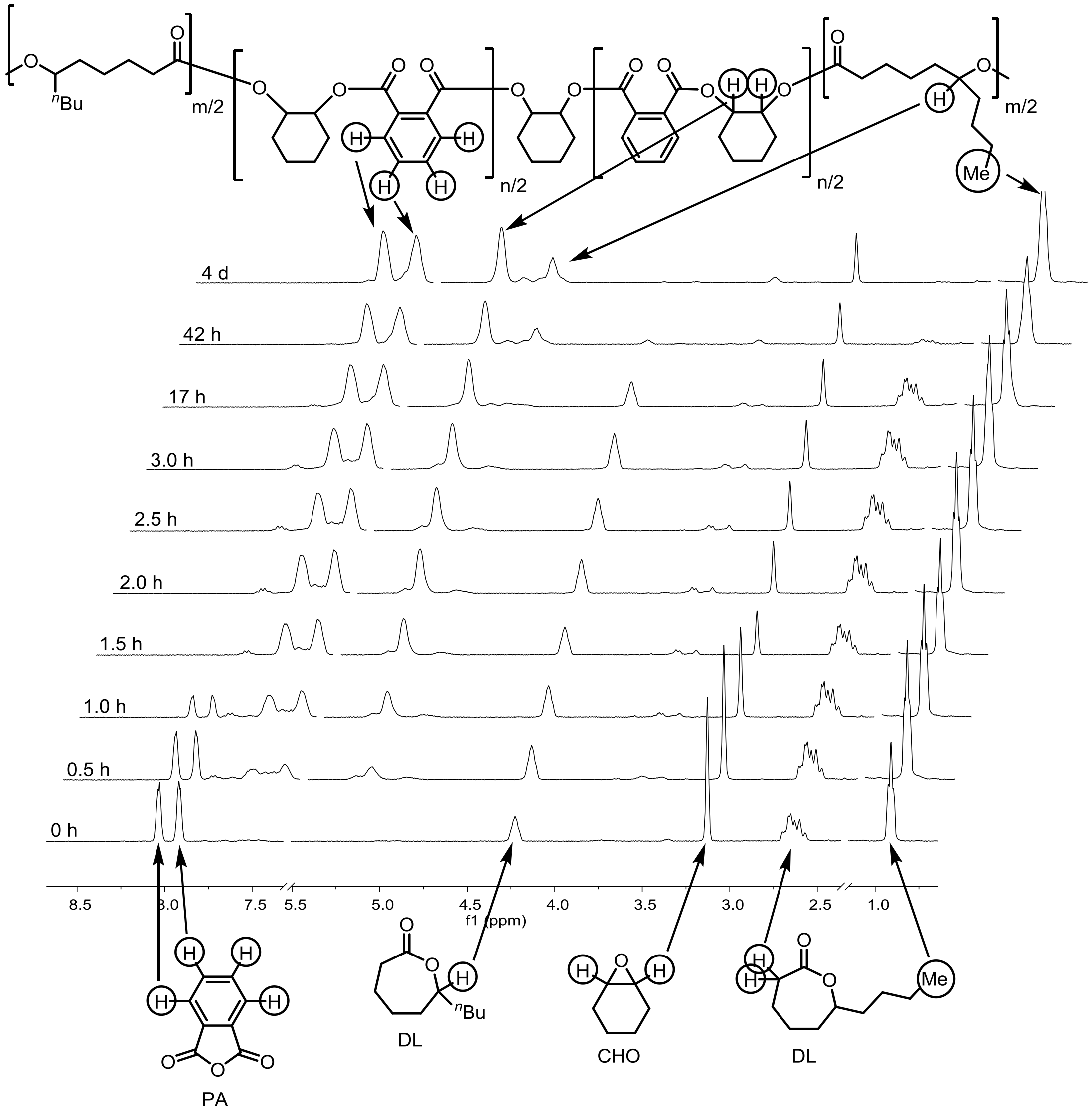

Figure S 30 - ^{1}H NMR spectra of polymerization of PA/CHO/DL.

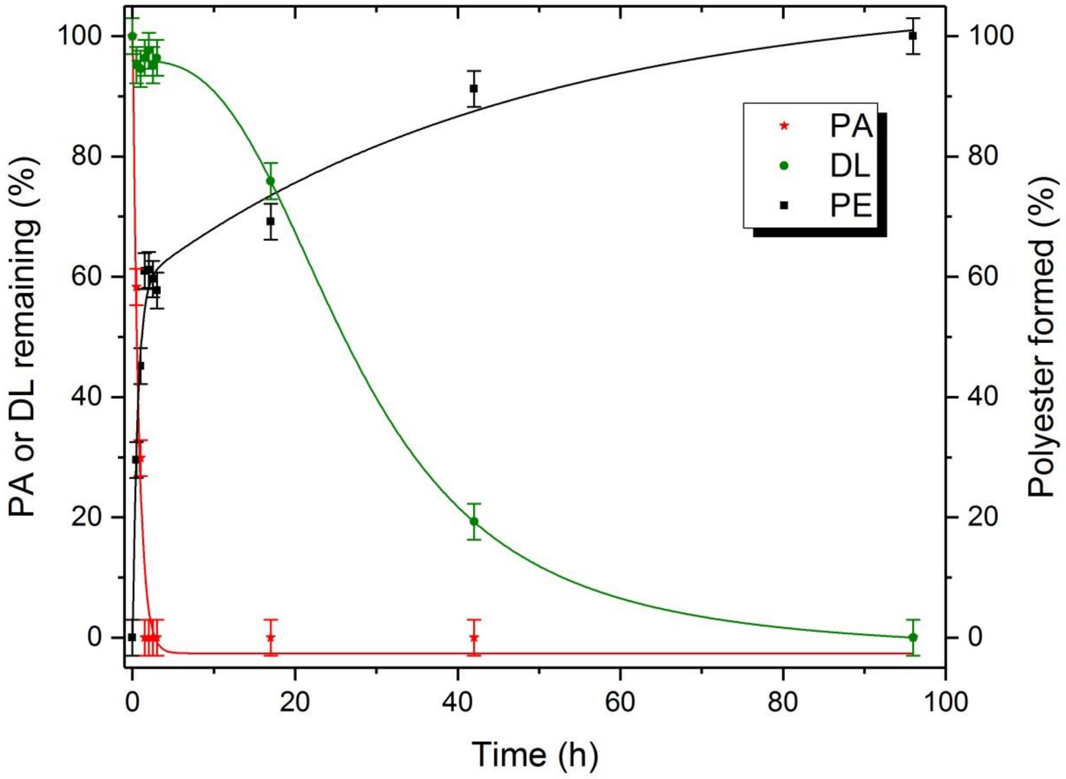

Figure S 31 - Conversion of PA and DL and formation of polyester; data is shown in Table S 5

Table S 5 - Integrals based on ^{1}H NMR spectra (after normalization with mesitylene as internal standard, Figure S 30) and calculated conversions used for Figure S 31. Signals were based on the following shifts: Signals were based on the following shifts: 7.87-8.08 (PA), 4.78 – 5.30 (Polyester), 4.23 (DL) and 3.10 (CHO) ppm. The conversion of CHO at the end of the reaction was determined as 76 % (data not shown).

Time (h)	Integral PA	PA (%)	Integral DL	DL (%)	Integral Polyester	Polyester (%)[a]
0	499	100	166	100	0	0
0.5	291	58.3	158	95.1	155	29.5
1	149	29.8	157	94.5	237	45.1
1.5	0	0	160	96.3	320	60.9
2	0	0	162	97.5	321	61.1
2.5	0	0	158	95.1	317	59.6
3	0	0	160	96.3	312	57.7
17	0	0	126	75.9	342	69.1
42	0	0	32	19.2	400	91.2
96	0	0	0	0	423	100

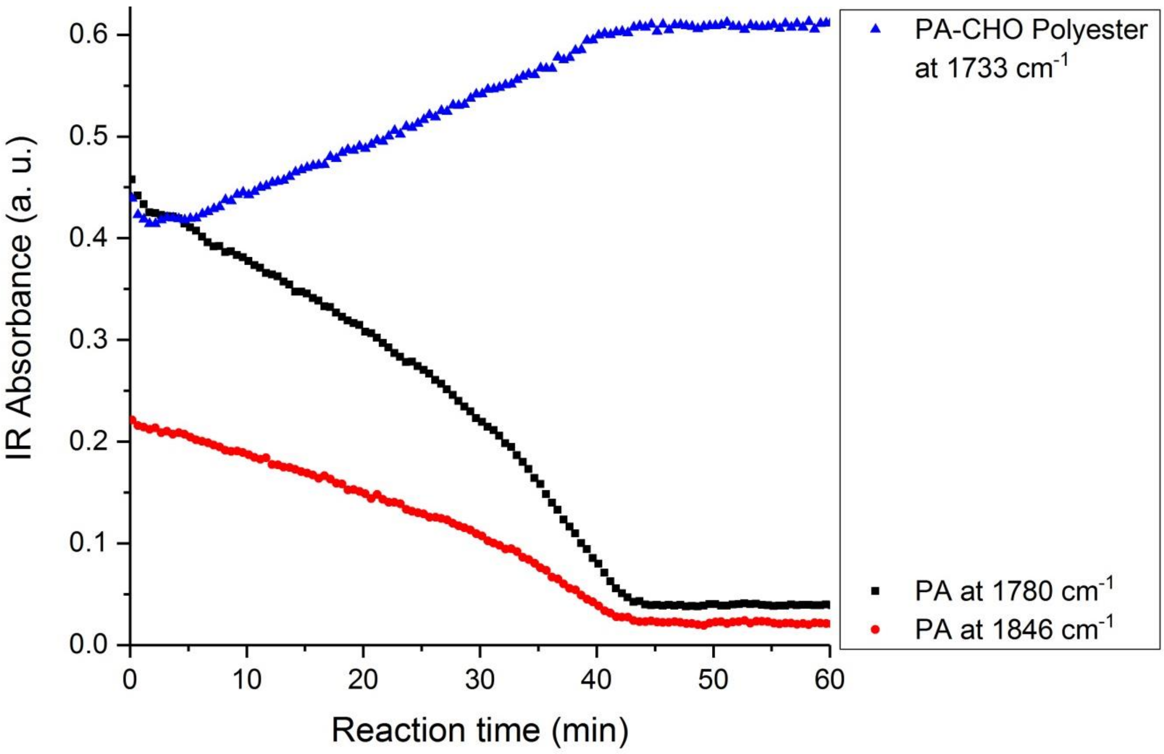

Figure S 32 - *In situ* monitoring of one-pot polymerization of PA/CHO/DL.

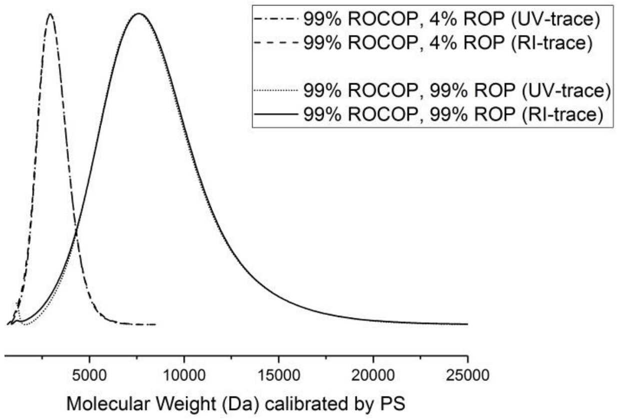

Figure S 33 – Comparison of GPC chromatograms after ROCOP (left peaks) and at the end of the reaction (right peaks); UV traces are shown in dots and RI traces as bold (or dashed) lines.

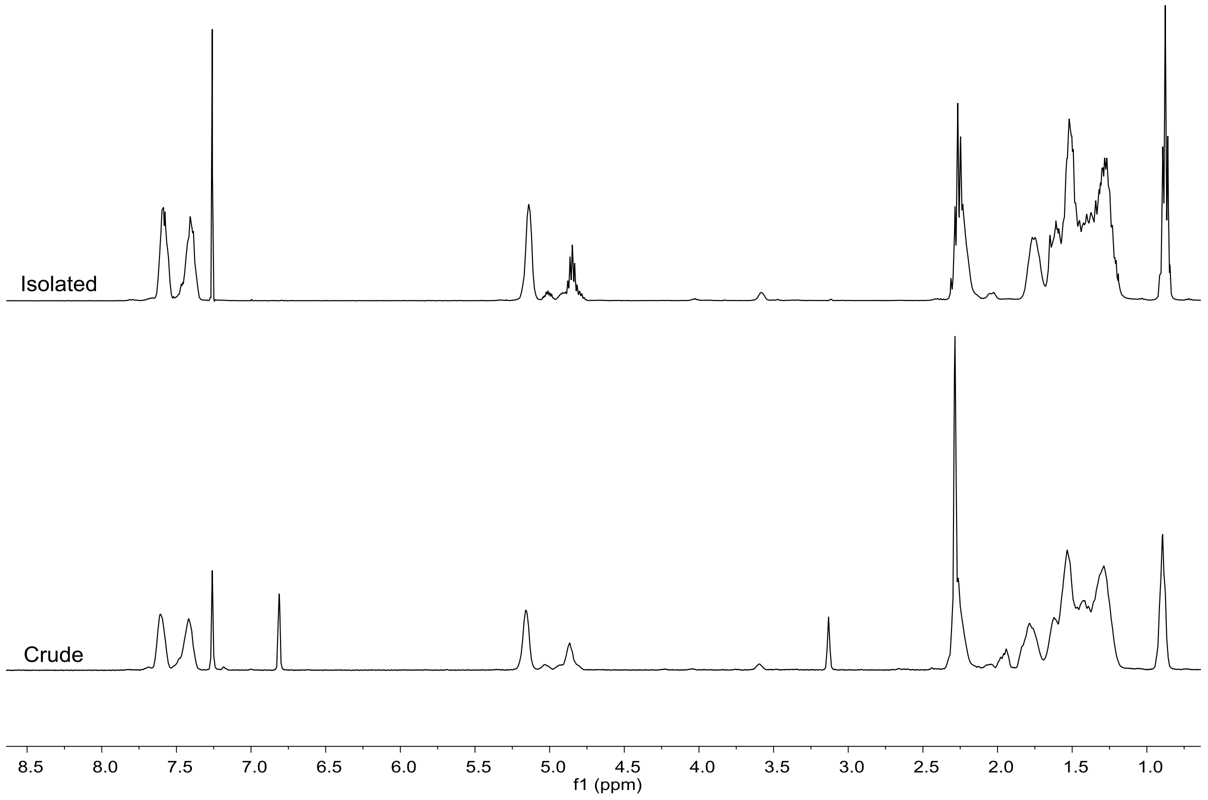

Figure S 34 – ^{1}H NMR spectra of crude (bottom) and isolated (top) polymer based on PA/CHO/DL.

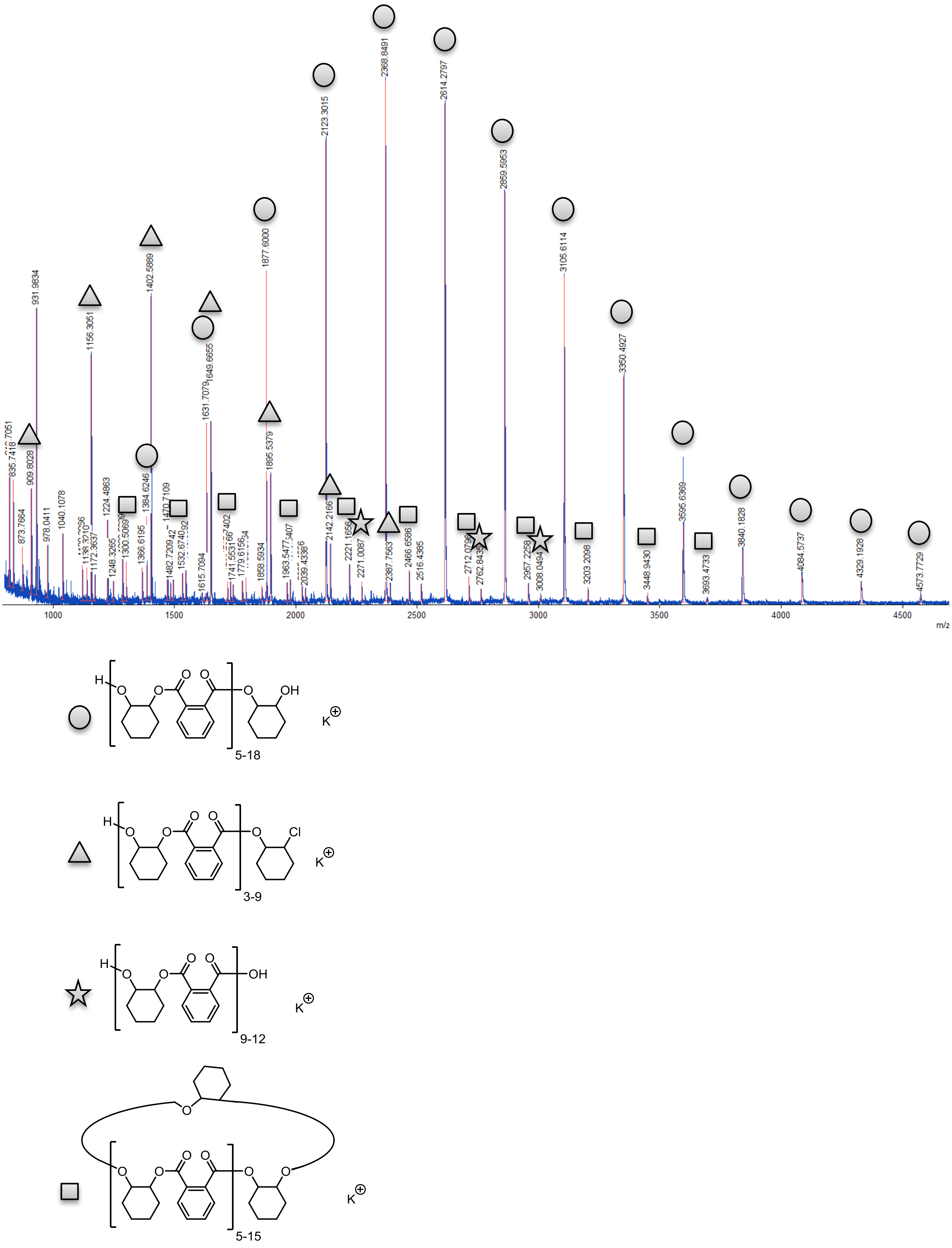

Figure S 35 – MALDI of polymerization of PA/CHO/DL (after 1.5 h, 99% PA and 4% DL conversion). Four species were detected: circle: m/z = [116.08 (cyclohexandiol) + (246.09 x n) (PCHPE) + 38.96 (K^+)] (n = 5 ~ 18); triangle: m/z = [34.97 ($Cl^·$) + 98.07 (CHO) + 1.01 ($H^·$) + (246.09 x n) (PCHPE) + 38.96 (K^+)] (n = 3 ~ 9); star: m/z = [18.01 (H_2O) + (246.09 x n) (PCHPE) + 38.96 (K^+)] (n = 9 ~ 12); square: m/z = [98.07 (CHO) + (246.09 x n) (PCHPE) + 38.96 (K^+)] (n = 5 ~ 15).

5.2. THPA/CHO/DL

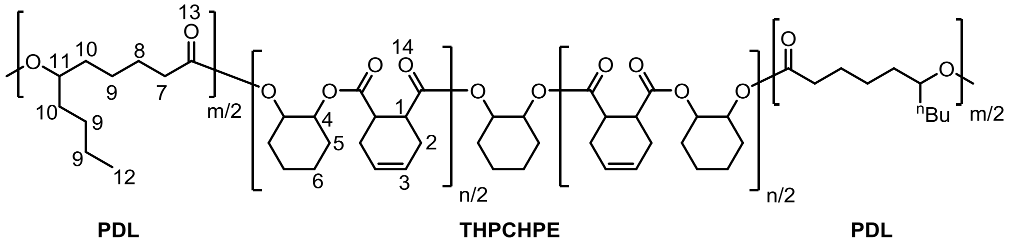

^{1}H NMR (500 MHz, $CDCl_3$) δ 5.61 (m, 1.00 H, H^{3}), 4.86-4.79 (m, 1.32 H, $H^{1,11}$), 3.58 (m, 0.13 H, $H^{11\text{-Endgrp}}$), 2.97-2.79 (m, 1.03 H, $H^{1,1\text{-Junction}}$), 2.46 (m, 0.84 H, H^{2}), 2.26 (m, 1.59 H, $H^{2,7'}$), 1.98 (m, 1.31 H, $H^{2\text{-Junction},5}$), 1.68 (m, 2.16 H, H^{6}), 1.52 (m, 1.43 H, $H^{8,10}$), 1.32 (m, 4.41 H, $H^{10\text{-Endgrp},5,9}$), 0.87 (m, 1.10H, H^{12}).

^{13}C NMR (126 MHz, $CDCl_3$) δ 173.63-173.44 (C^{13}), 172.55 (C^{14}), 125.26 (C^{3}), 74.00 (C^{11}), 73.27 (C^{4}), 71.77 ($C^{11\text{-Endgrp}}$), 41.03 ($C^{1\text{-Junction}}$), 39.80 (C^{1}), 37.35 (C^{10}), 37.19 (C^{7}), 34.62-33.93 (C^{8}), 29.90 (C^{5}), 27.98-27.58 ($C^{9,2\text{-Junction}}$), 25.85-25.13 (C^{8}), 23.33 (C^{6}), 22.70 (C^{9}), 14.14 (C^{12}).

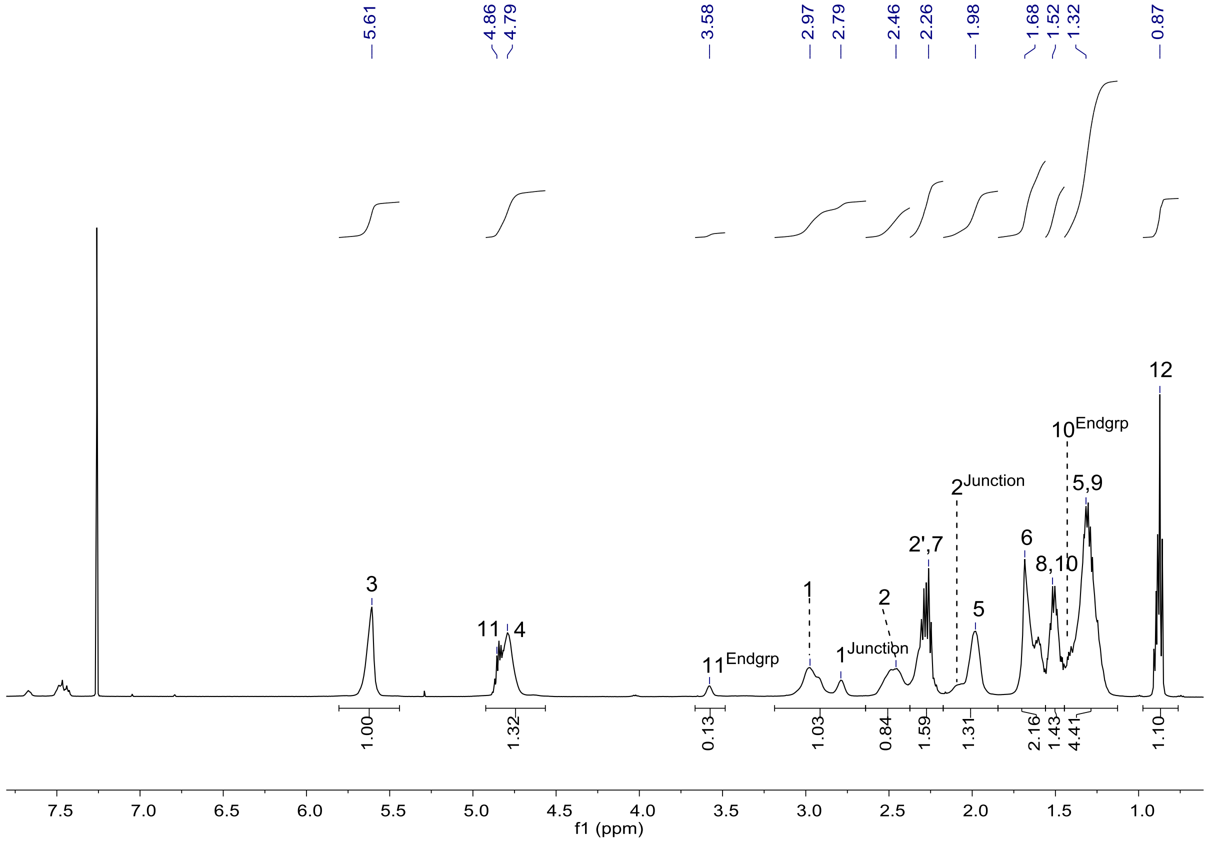

Figure S 36 - ^{1}H NMR spectrum of polymer based on THPA, CHO, DL.

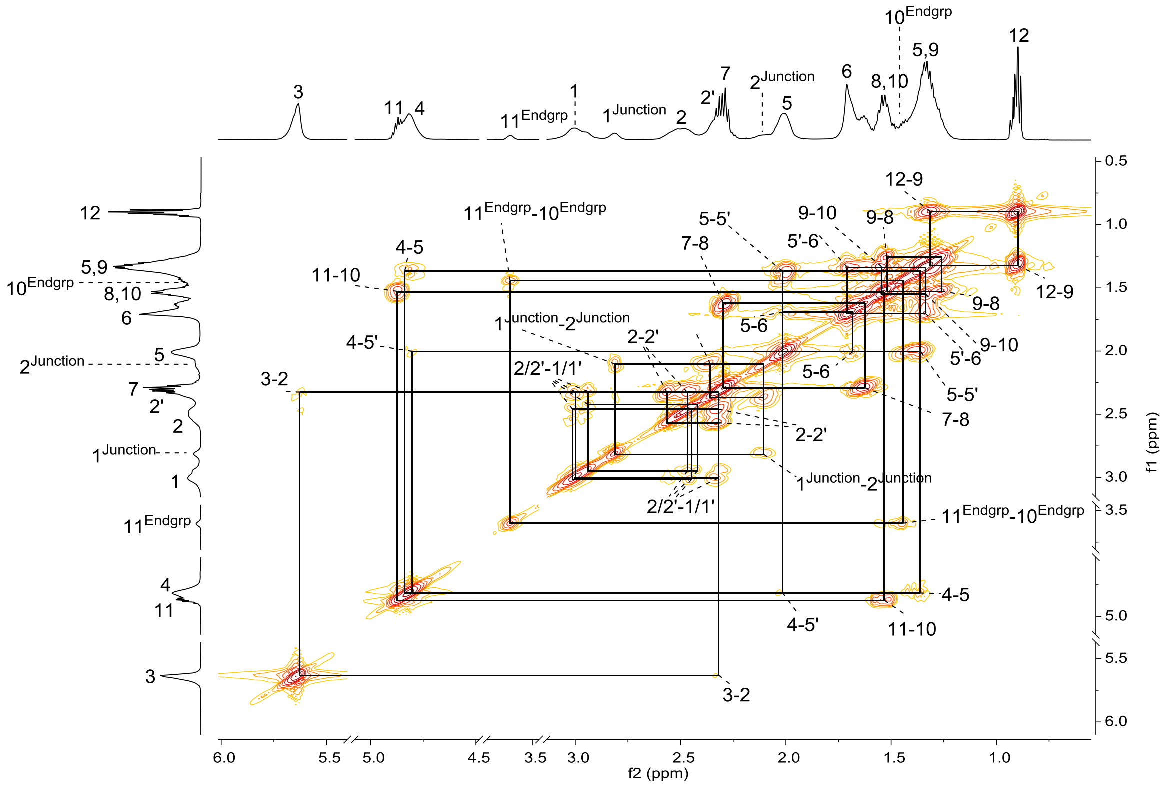

Figure S 37 - COSY NMR spectrum of polymer based on THPA, CHO, DL.

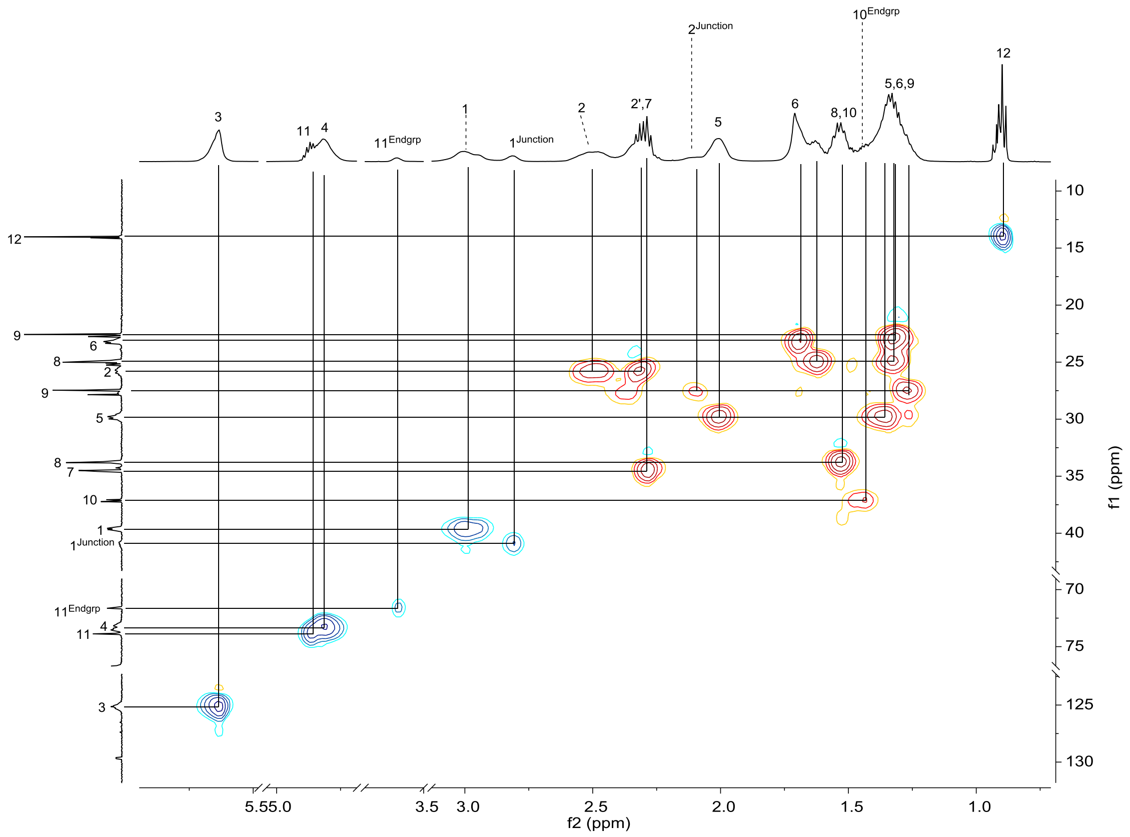

Figure S 38 - $^{1}H,^{13}C$-HSQC of polymer based on THPA, CHO, DL.

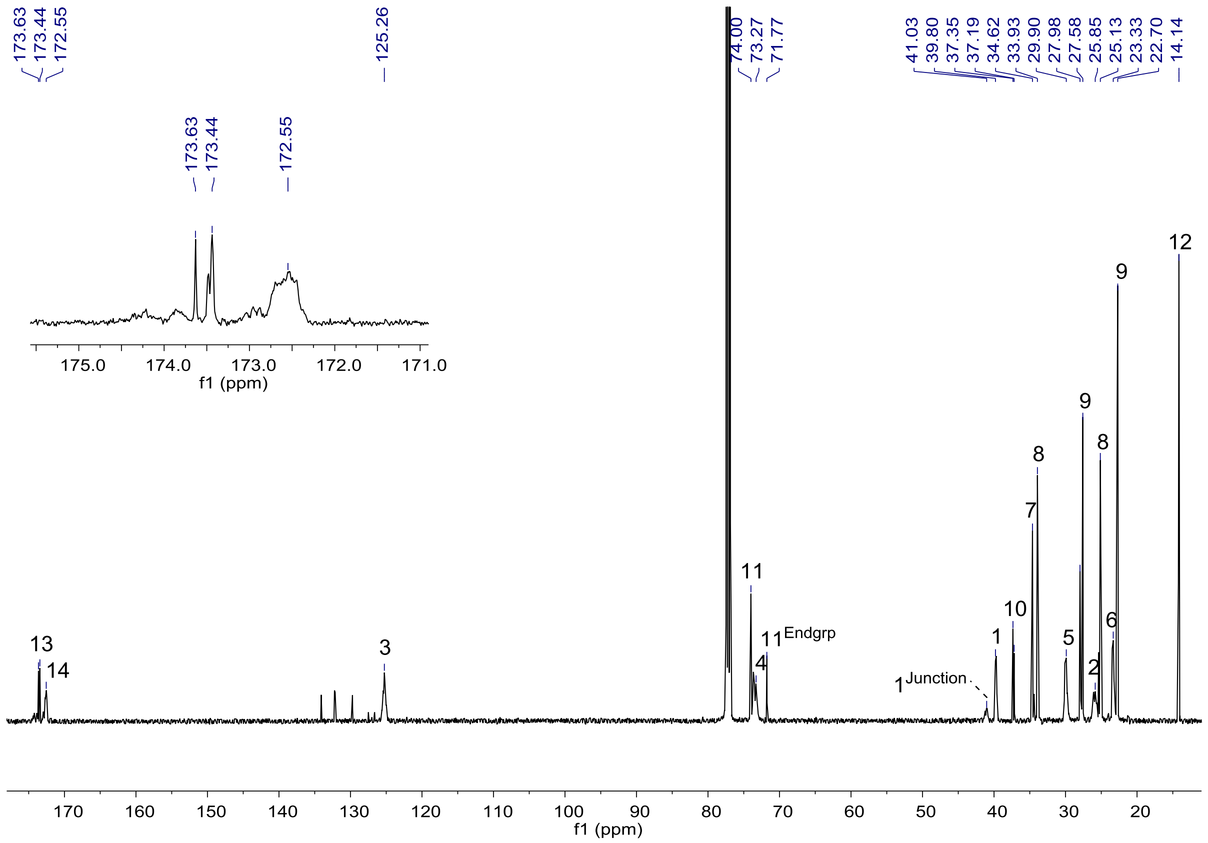

Figure S 39 - $^{13}C\{^{1}H\}$ NMR spectrum of polymer based on THPA, CHO, DL. Traces of PPNCl were observed at 129.8, 132.2 and 134.1 ppm.

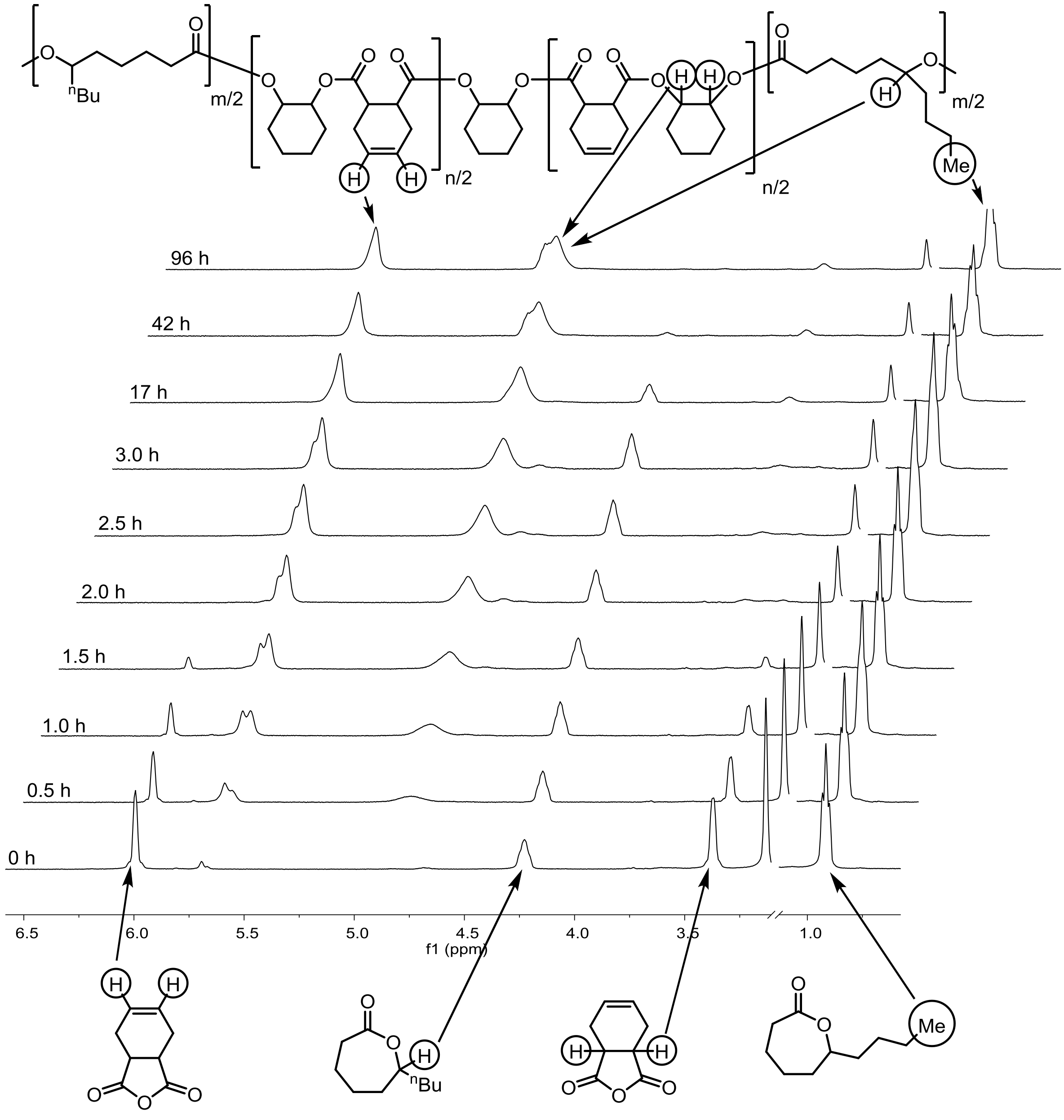

Figure S 40- ^{1}H NMR spectra of polymerization of THPA/CHO/DL.

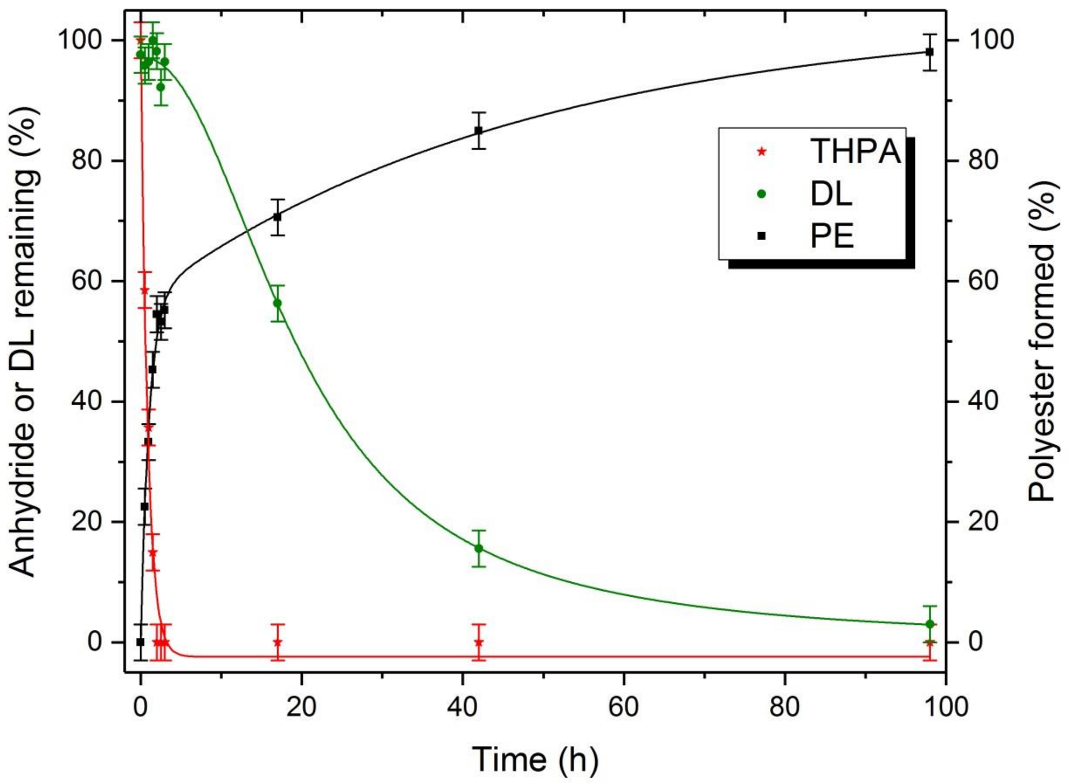

Figure S 41 - Conversion of THPA and DL and formation of polyester. Data is given in Table S 6.

Table S 6 - Integrals based on ^{1}H NMR spectra (after normalization with mesitylene, Figure S 40) and calculated conversions used for Figure S 41. Signals were based on the following shifts: 6.60 (THPA), 4.67-4.95 (Polyester), 4.23 (DL) and 3.10 (CHO) ppm. The conversion of CHO at the end of the reaction was determined as 79 % (data not shown).

Time (h)	Integral THPA	THPA (%)	Integral DL	DL (%)	Integral Polyester	Polyester (%)[a]
0	241	100	163	97.6	0	0
0.5	141	58.5	160	95.8	105	22.5
1	86	35.6	161	96.4	155	33.2
1.5	36	14.9	167	100	211	45.2
2	0	0	164	98.2	254	54.5
2.5	0	0	154	92.2	248	53.2
3	0	0	161	96.4	257	55.1
17	0	0	94	56.2	329	70.6
42	0	0	26	15.5	396	84.9
96	0	0	6	3	420	98

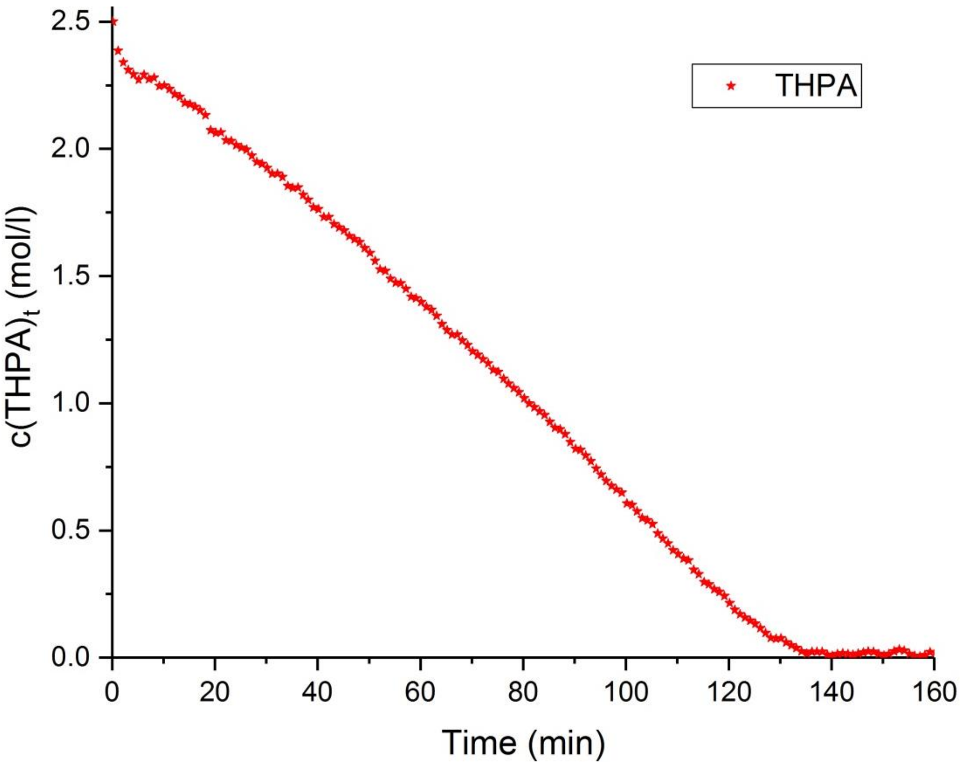

Figure S 42 - *In situ* monitoring of IR band of THPA at 1850 cm^{-1}.

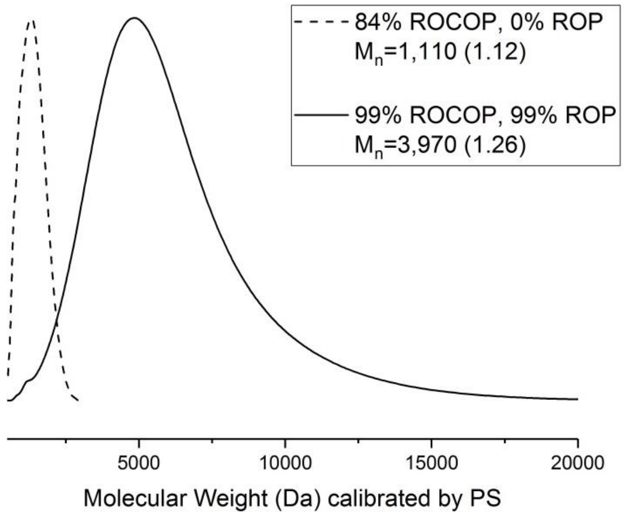

Figure S 43 –GPC chromatograms at 84% conversion of ROCOP and at the end of the reaction.

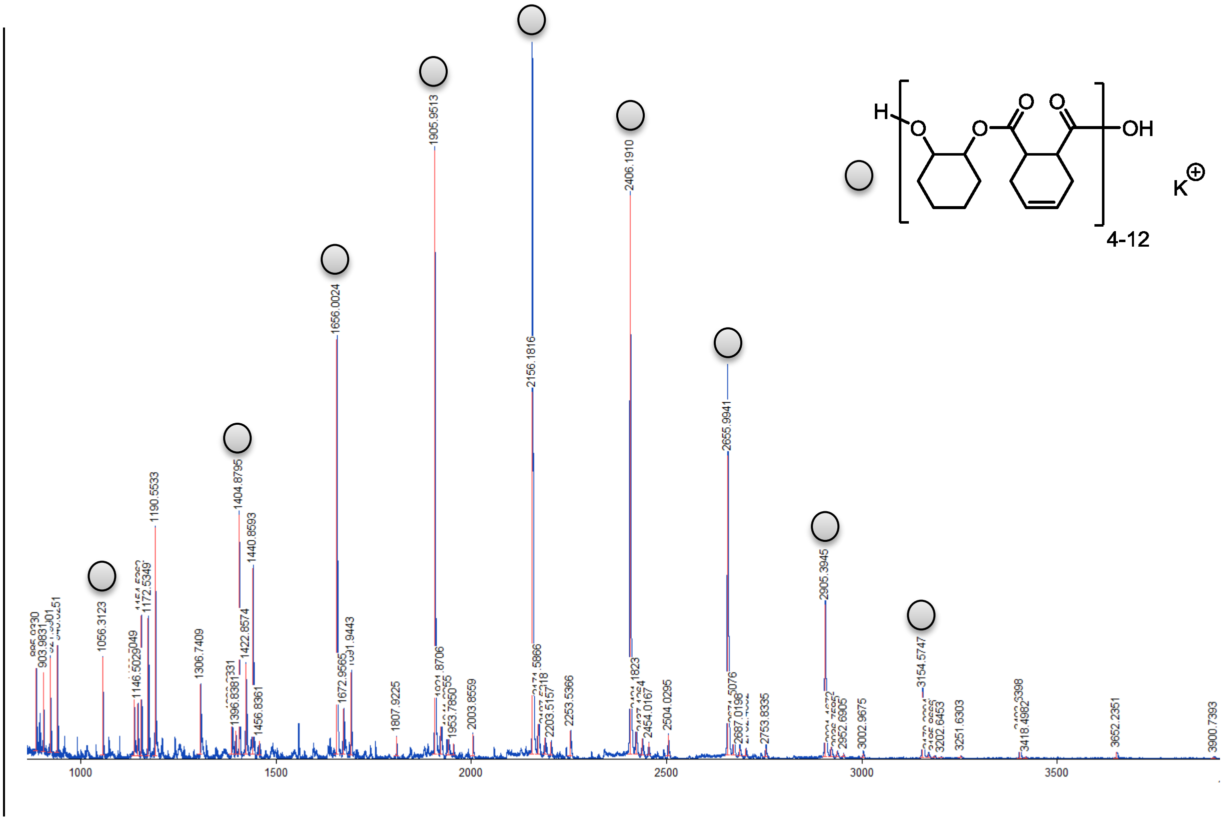

Figure S 44 – MALDI of polymer based on THPA/CHO/DL (after 3 h, 99% THPA and 4% DL conversion). The series shows: m/z = [116.08 (cyclohexandiol) + (244.09 x n) (PCHTHPE) + 38.96 (K^+)] (n = 5 ~ 18).

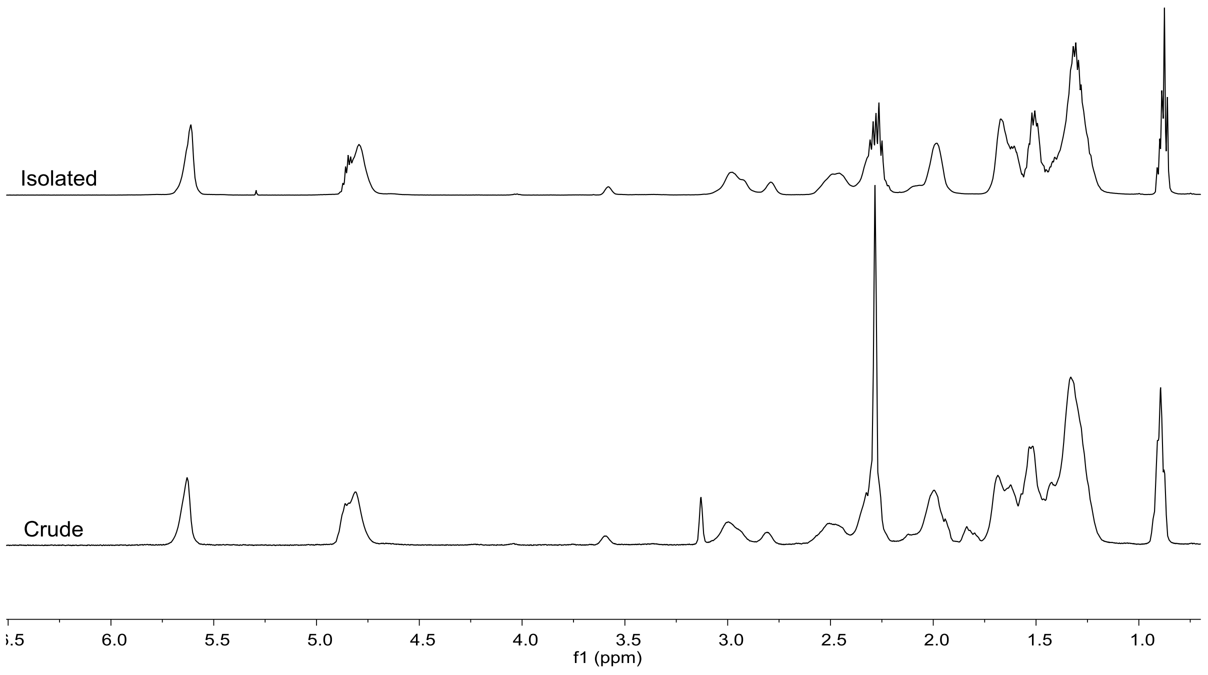

Figure S 45 – ^{1}H NMR spectra of crude (bottom) and isolated (top) polymer based on THPA/CHO/DL.

5.3. TCA1/CHO/DL

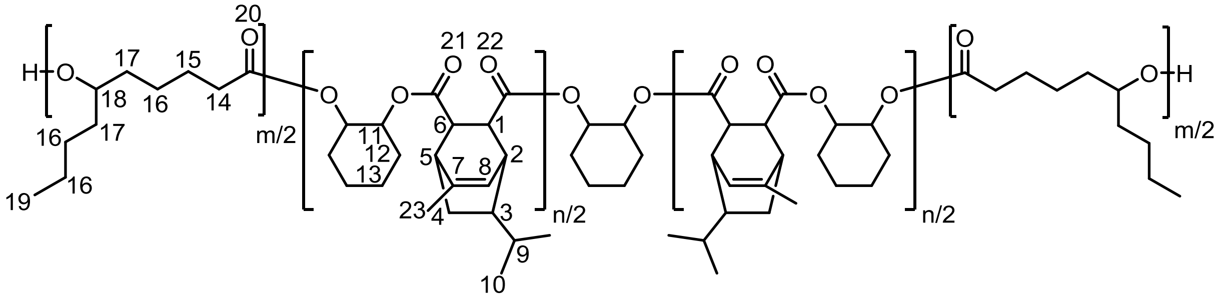

PDL **PCHTCE1** **PDL**

^{1}H NMR (500 MHz, $CDCl_3$) δ 5.80-5.57 (m, 1.00 H, H^8), 4.84-4.65 (m, 2.87 H, $H^{1,6,18}$), 3.58 (m, 0.16 H, $H^{18\text{-Endgrp}}$), 2.97-2.66 (m, 3.86 H, $H^{2,5,1,6}$), 2.26 (m, 1.92 H, H^{14}), 2.08-1.83 (m, 3.09 H, H^{12}), 1.83-1.70 (m, 2.14 H, $H^{9,23}$), 1.70-1.56 (m, 3.84 H, $H^{4,15}$), 1.56-1.45 (m, 3.84 H, $H^{17,3}$), 1.44-1.16 (m, 10.63 H, $H^{12',13,16}$), 1.16-1.00 (m, 1.15 H, H^9), 0.89-0.80 (m, 6.37 H, $H^{10,19}$), 0.80-0.66 (m, 2.99 H, $H^{10'}$).

^{13}C NMR (126 MHz, $CDCl_3$) δ 173.51-171.68 ($C^{20,21,22}$), 124.32-121.11 (C^8), 73.88 ($C^{11,18}$), 71.64 ($C^{18\text{-Endgrp}}$), 48.96-44.53 ($C^{2,5,1,6}$), 38.88-31.69 ($C^{2,5,1,6}$), 37.23 (C^{16}), 34.50 (C^{14}), 33.81(C^{17}), 33.20-32.12 (C^9), 31.69-32.12 ($C^{2,5,1,6}$), 29.80(C^{12}), 27.85 (C^{17}), 27.46 (C^{13}), 25.25-25.01 ($C^{15,16}$), 22.76 ($C^{17,\text{Endgrp}}$), 22.58 ($C^{4,16}$), 21.17 ($C^{10,23}$), 20.21 ($C^{10',23}$), 14.01 (C^{19}).

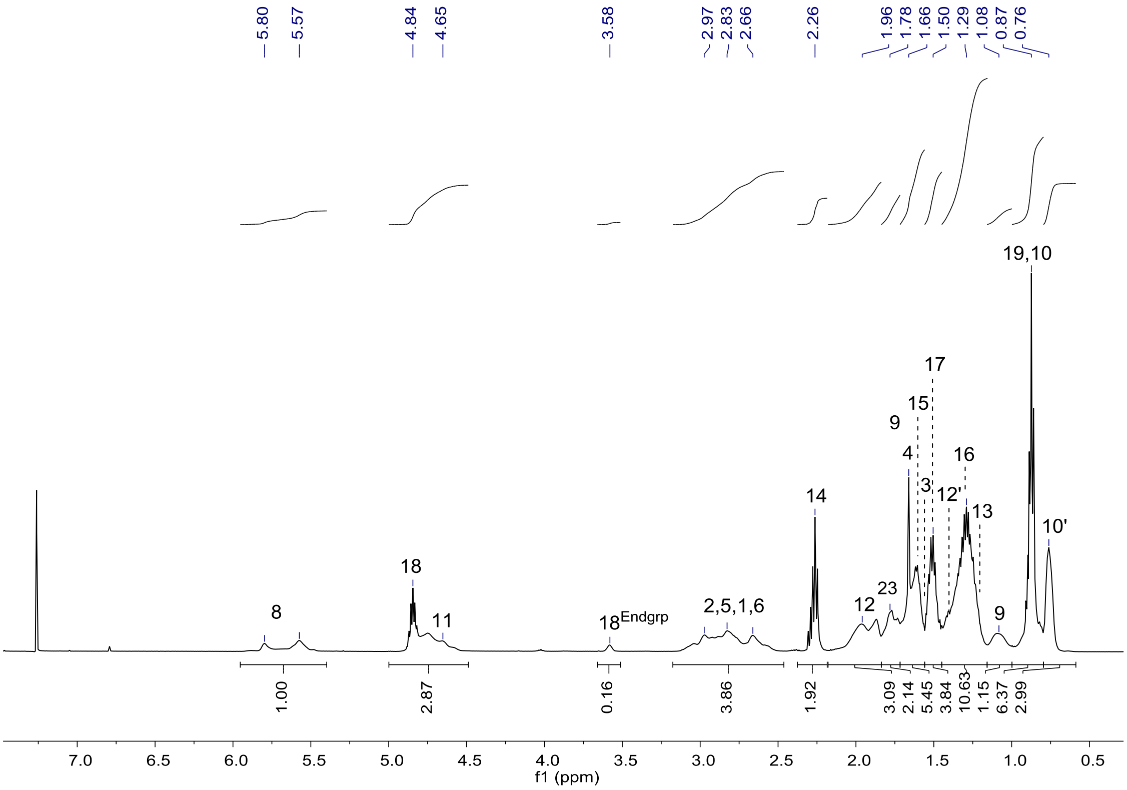

Figure S 46 - ^{1}H NMR spectrum of polymer based on TCA1, CHO, DL.

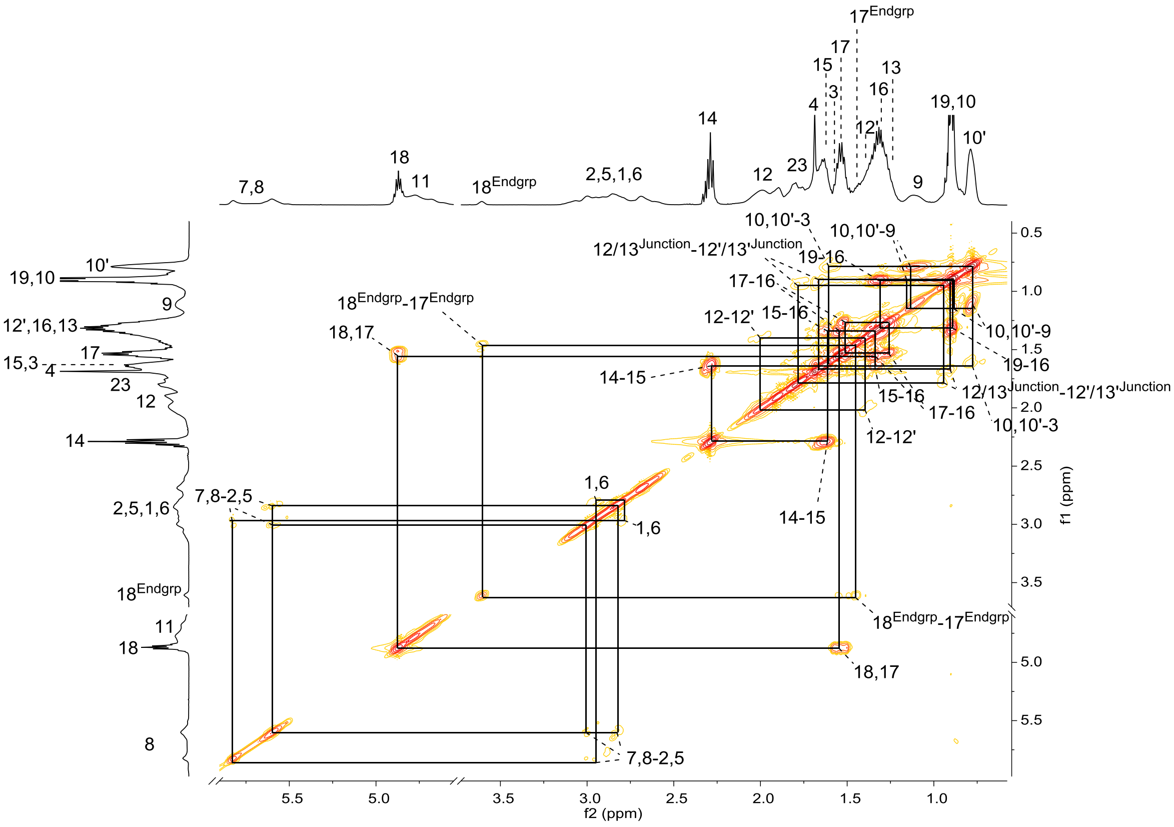

Figure S 47 - COSY NMR spectrum of polymer based on TCA1, CHO, DL.

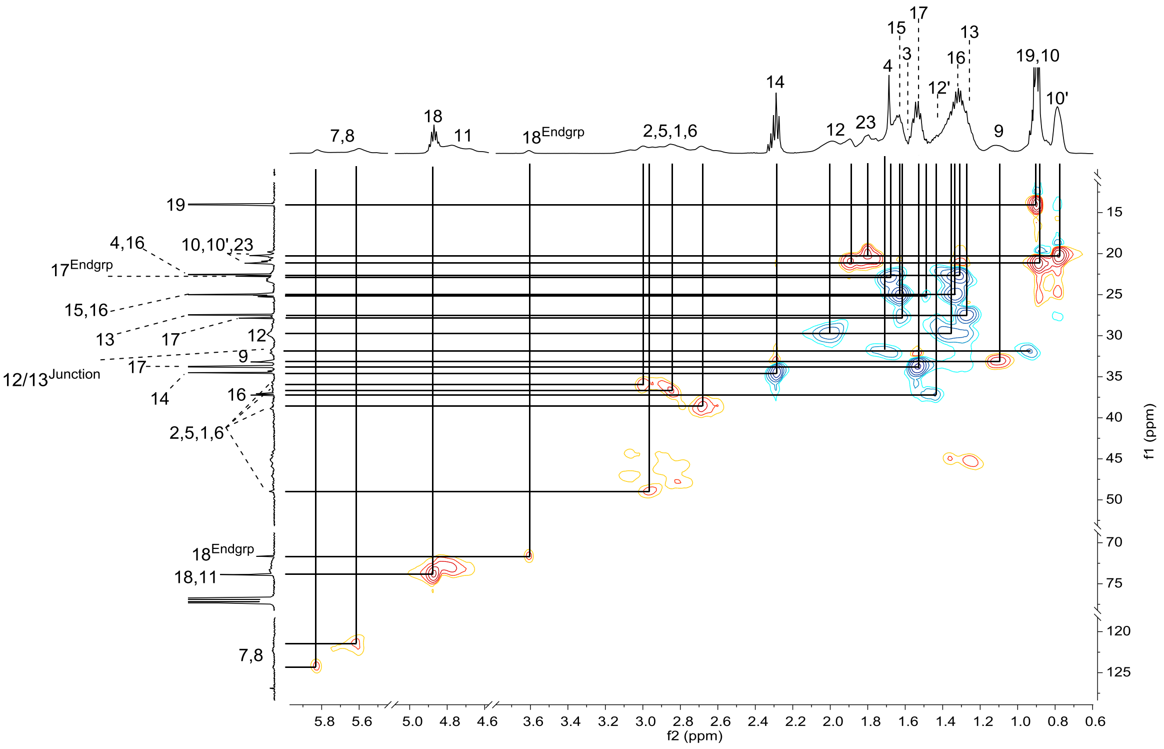

Figure S 48 - HSQC NMR spectrum of polymer based on TCA1, CHO, DL.

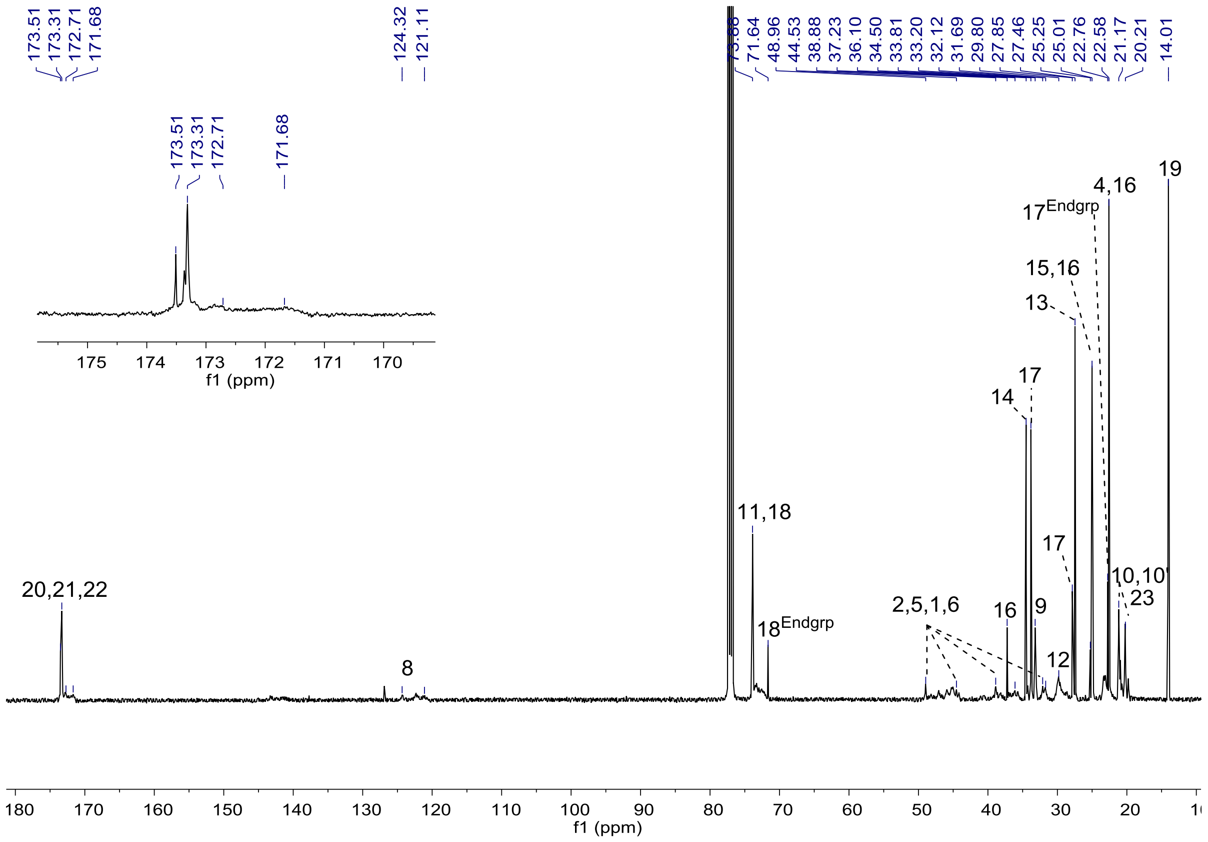

Figure S 49 - $^{13}C\{^{1}H\}$ NMR spectrum of polymer based on TCA1, CHO, DL. Traces of PPNCl were observed at 129.8, 132.2 and 134.1 ppm.

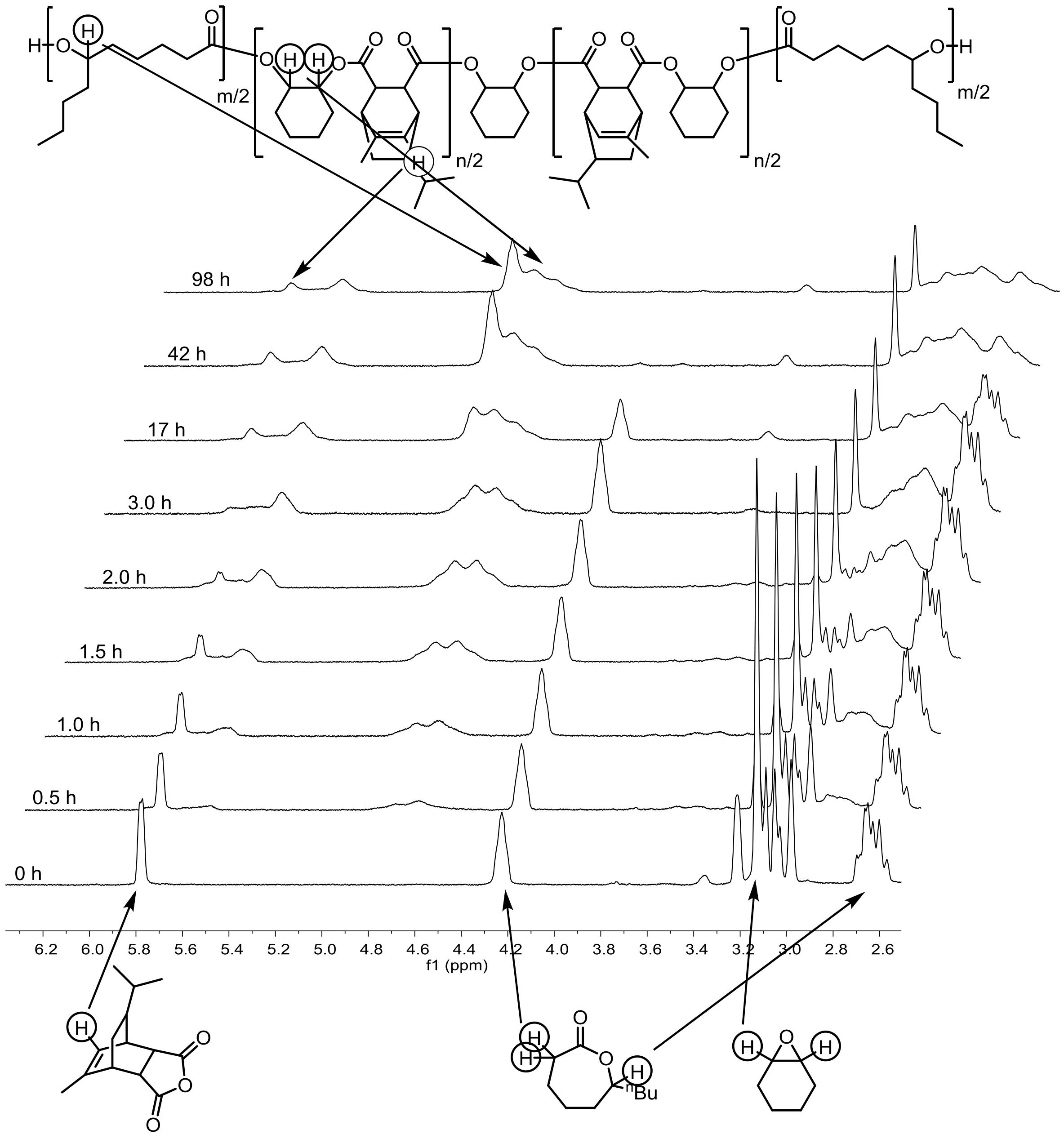

Figure S 50 - ^{1}H NMR spectra of one-pot polymerization of TCA1/CHO/DL.

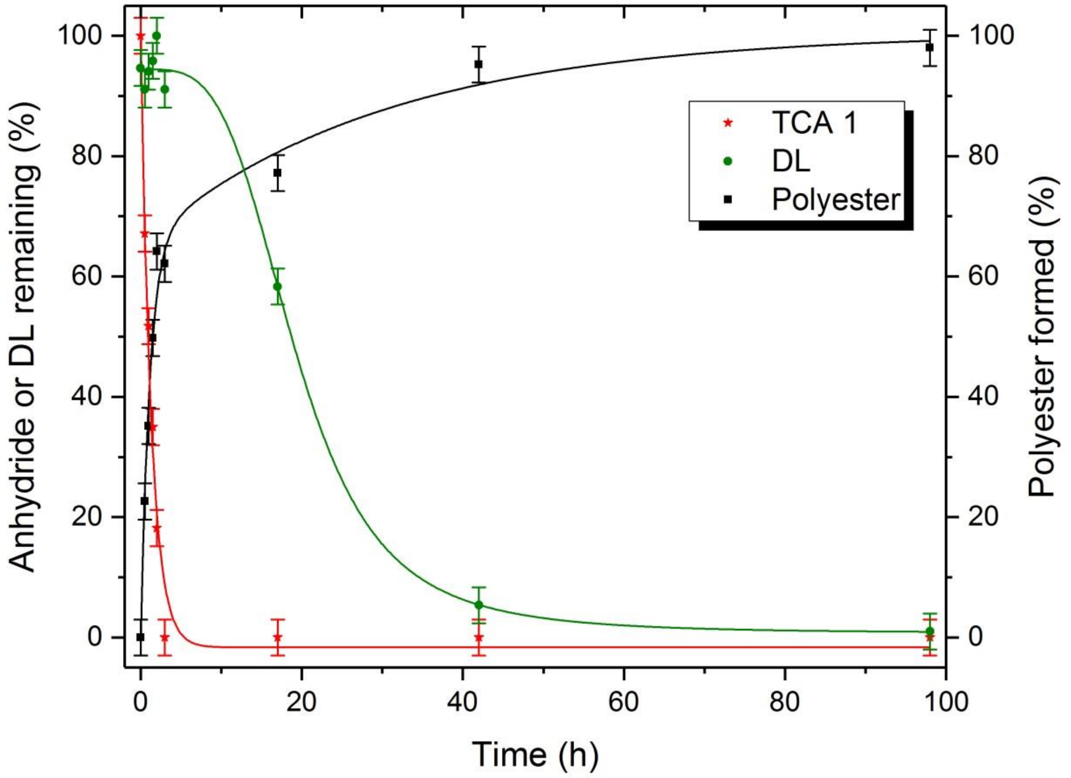

Figure S 51 - Conversion of TCA1 and DL and formation of polyester Data was obtained after normalization with mesitylene as internal standard.

Table S 7 - Integrals based on ^{1}H NMR spectra (after normalization with mesitylene, Figure S 40) and calculated conversions used for Figure S 41. Signals were based on the following shifts: 3.21 (TCA1), 4.45-4.92 (Polyester), 4.23 (DL) and 3.10 (CHO) ppm. The conversion of CHO at the end of the reaction was determined as 77 % (data not shown).

Time (h)	Integral TCA1	TCA1 (%)	Integral DL	DL (%)	Integral Polyester	Polyester (%)[a]
0	143	100	159	94.6	0	0
0.5	96	67.1	153	91.0	99	22.6
1	74	51.7	158	94.0	154	35.1
1.5	50	34.9	161	95.8	218	49.7
2	26	18.1	168	100	281	64.1
3	0	0	153	91.0	272	62.1
17	0	0	98	58.3	338	77.1
42	0	0	9	5.3	417	95.2
96	0	0	2	1	430	98.0

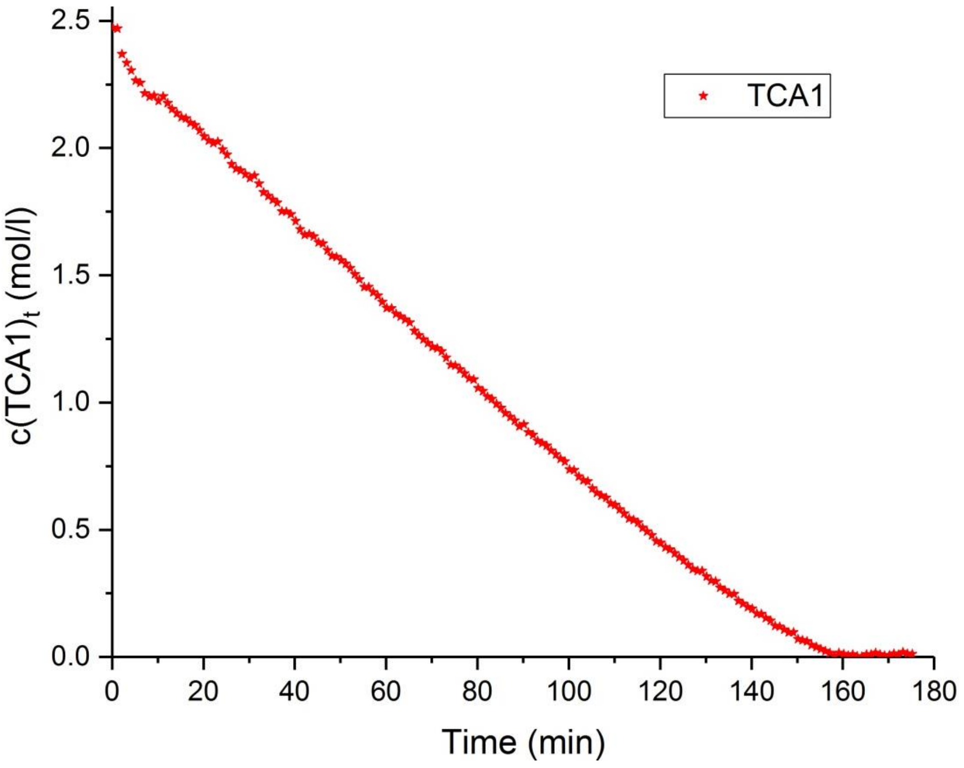

Figure S 52 - *In situ* monitoring of IR band of TAC1 at 1850 cm^{-1}.

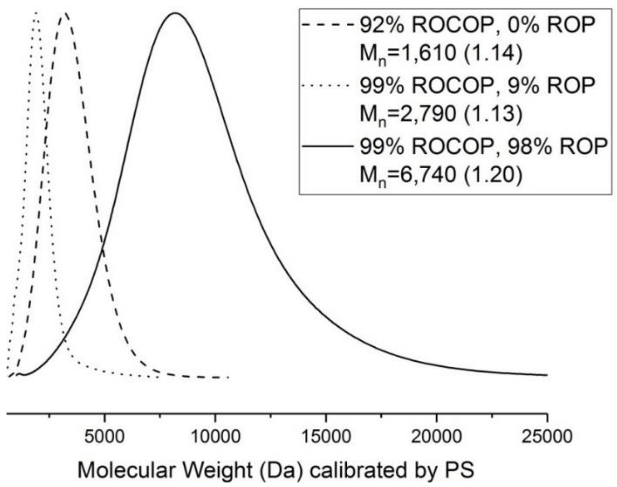

Figure S 53 –GPC chromatogram at different conversions for TCA1/CHO/DL.

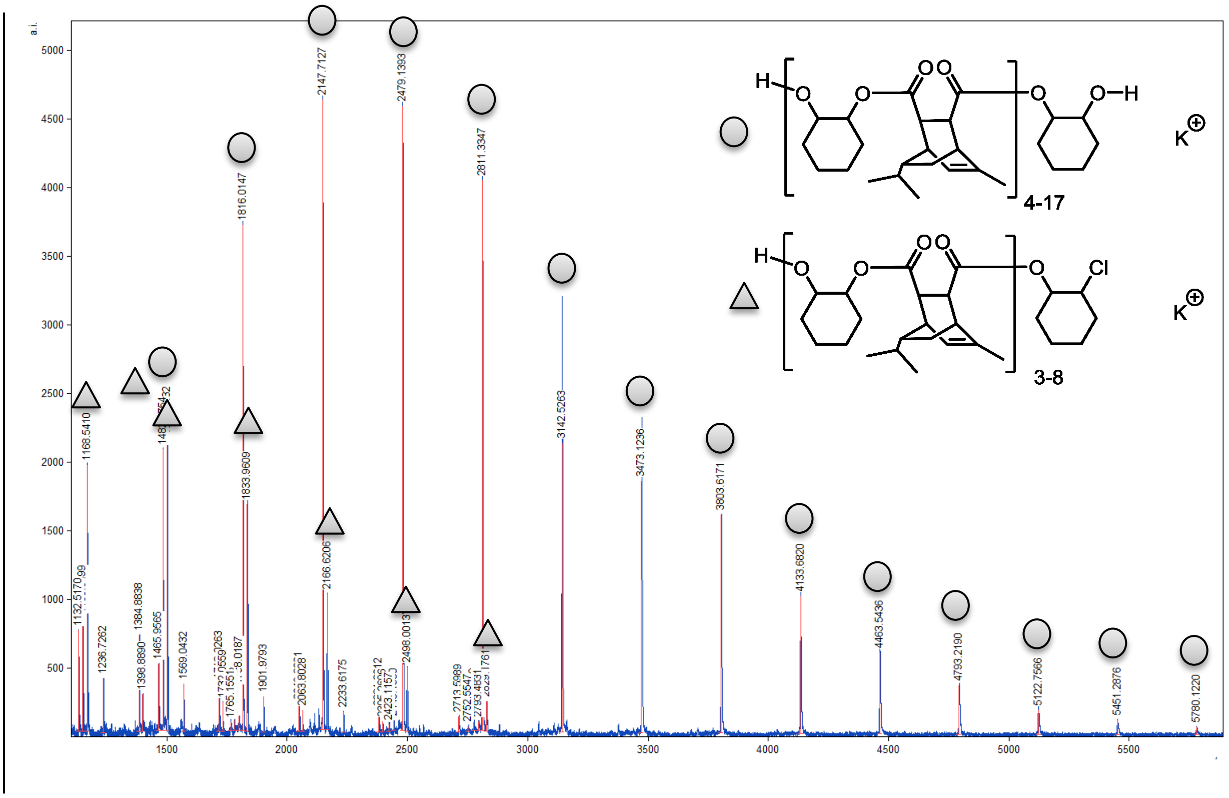

Figure S 54 – MALDI of one-pot polymerization of TCA1/CHO/DL (after 2 h, 82% TCA1 and 0% DL conversion). Two species were detected: circle: m/z = [116.08 (cyclohexandiol) + (332.2 x n) (PCHTCE1) + 38.96 (K^+)] (n = 5 ~ 18); triangle: m/z = [34.97 ($Cl^·$) + 98.07 (CHO) + 1.01 ($H^·$) + (332.2 x n) (PCHTCE1) + 38.96 (K^+)] (n = 3 ~ 8).

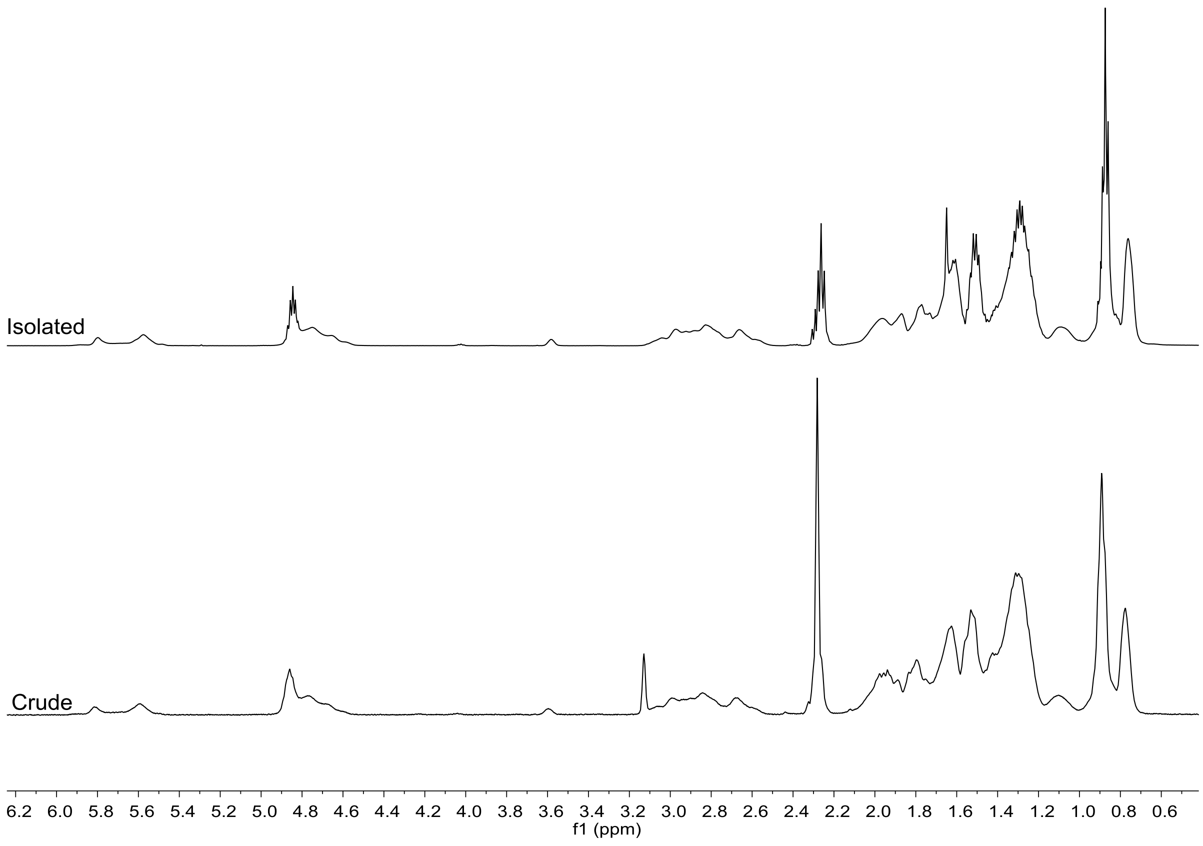

Figure S 55 – ^{1}H NMR spectra of crude (bottom) and isolated (top) polymer based on TCA1/CHO/DL.

5.4. TCA2/CHO/DL

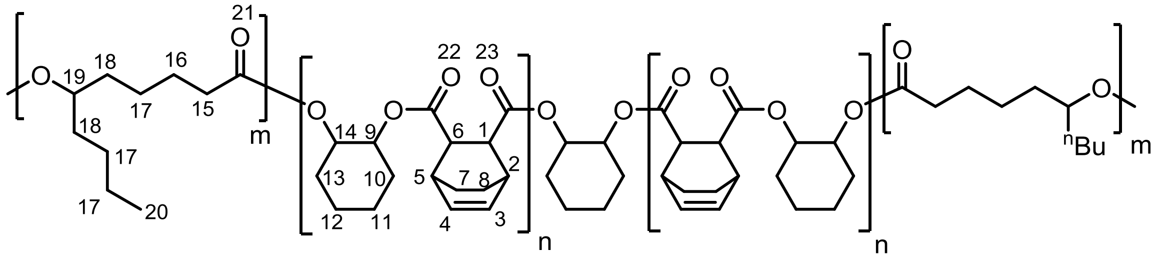

PDL **PCHTCE2** **PDL**

^{1}H NMR (500 MHz, CDCl$_3$) δ 6.32-6.13 (m, 1.00 H, $H^{3,4}$), 4.84-4.68 (m, 1.35 H, $H^{9,14,19}$), 3.58 (m, 0.09 H, $H^{19\text{-Endgroup}}$), 2.93-2.85 (m, 1.89 H, $H^{2,5}$), 2.26 (m, 0.77 H, H^{15}), 1.97 (m, 1.05 H, $H^{10,13}$), 1.64 (m, 2.24 H, $H^{15,11,12}$), 1.50 (m, 2.04 H, H^{18}), 1.28 (m, 5.27 H, $H^{7,8,10',13,16,17}$),1.10 (m, 0.24H, $H^{17\text{-Endgroup}}$), 0.87 (m, 1.15 H, H^{20}).

^{13}C NMR (126 MHz, CDCl$_3$) δ 173.63-171.92 ($C^{21,22,23}$), 74.01 (C^{19}), 73.01 ($C^{4,9}$), 71.75 ($C^{19\text{-Endgroup}}$), 47.96-45.42 ($C^{2,5}$), 37.34 ($C^{7,8,10,13,16,17}$), 34.70 (C^{15}), 34.61 (C^{18}), 33.93-22.88 ($C^{7,8,10,13,16,17}$), 22.69 ($C^{17\text{-Endgroup}}$), 14.13 (C^{20}).

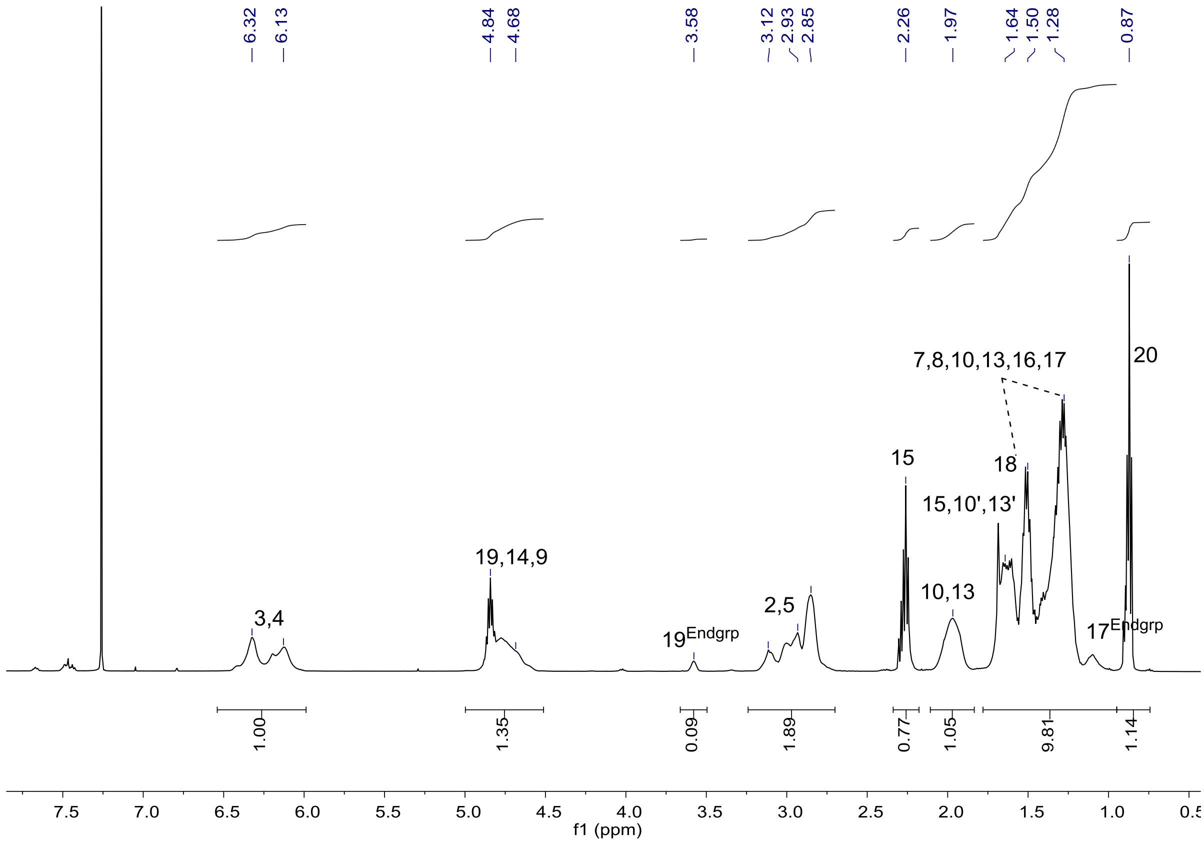

Figure S 56 – ^{1}H NMR spectrum of polymer based on TCA2, CHO, DL.

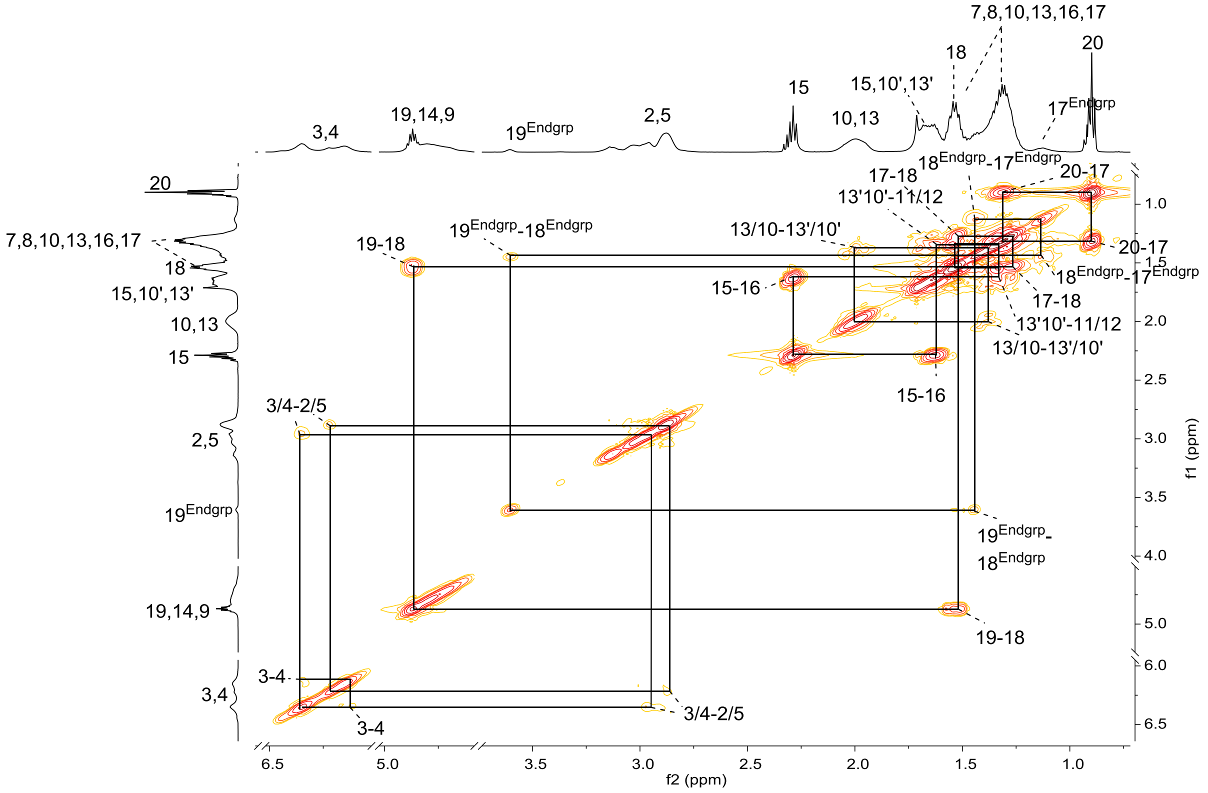

Figure S 57 - COSY NMR spectrum of polymer based on TCA2, CHO, DL.

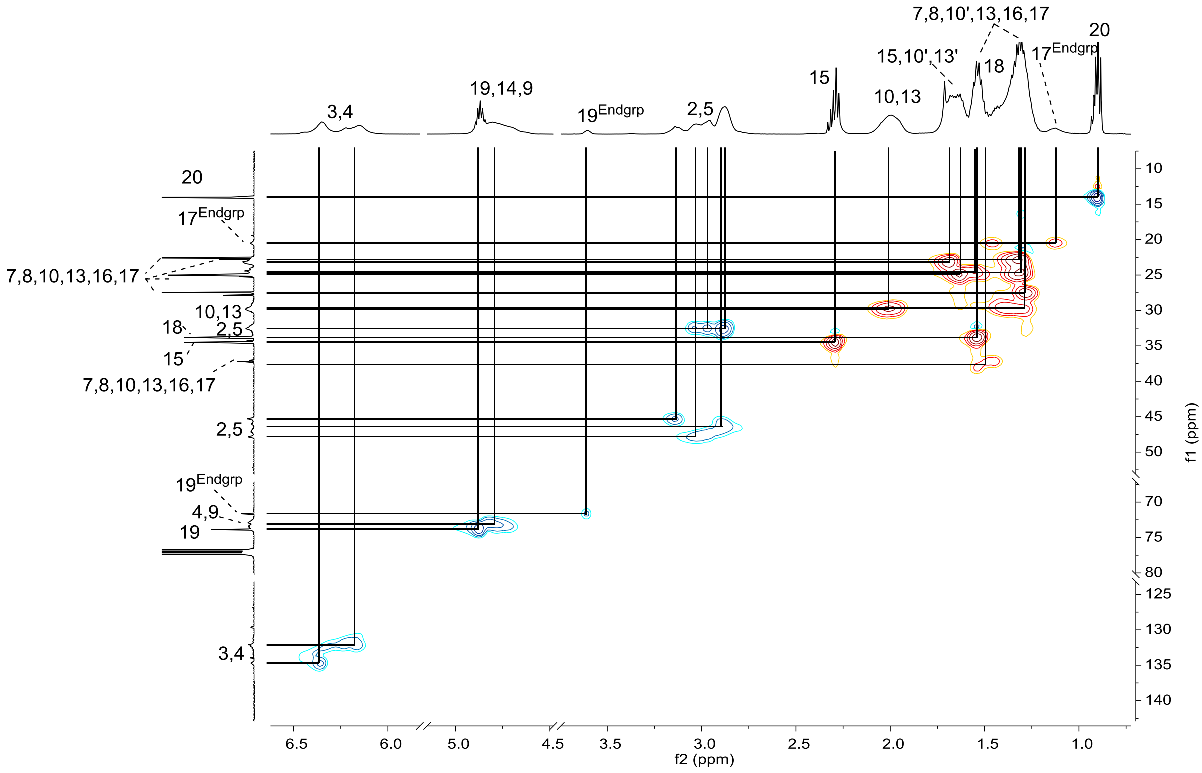

Figure S 58 - HSQC NMR spectrum of polymer based on TCA2, CHO, DL.

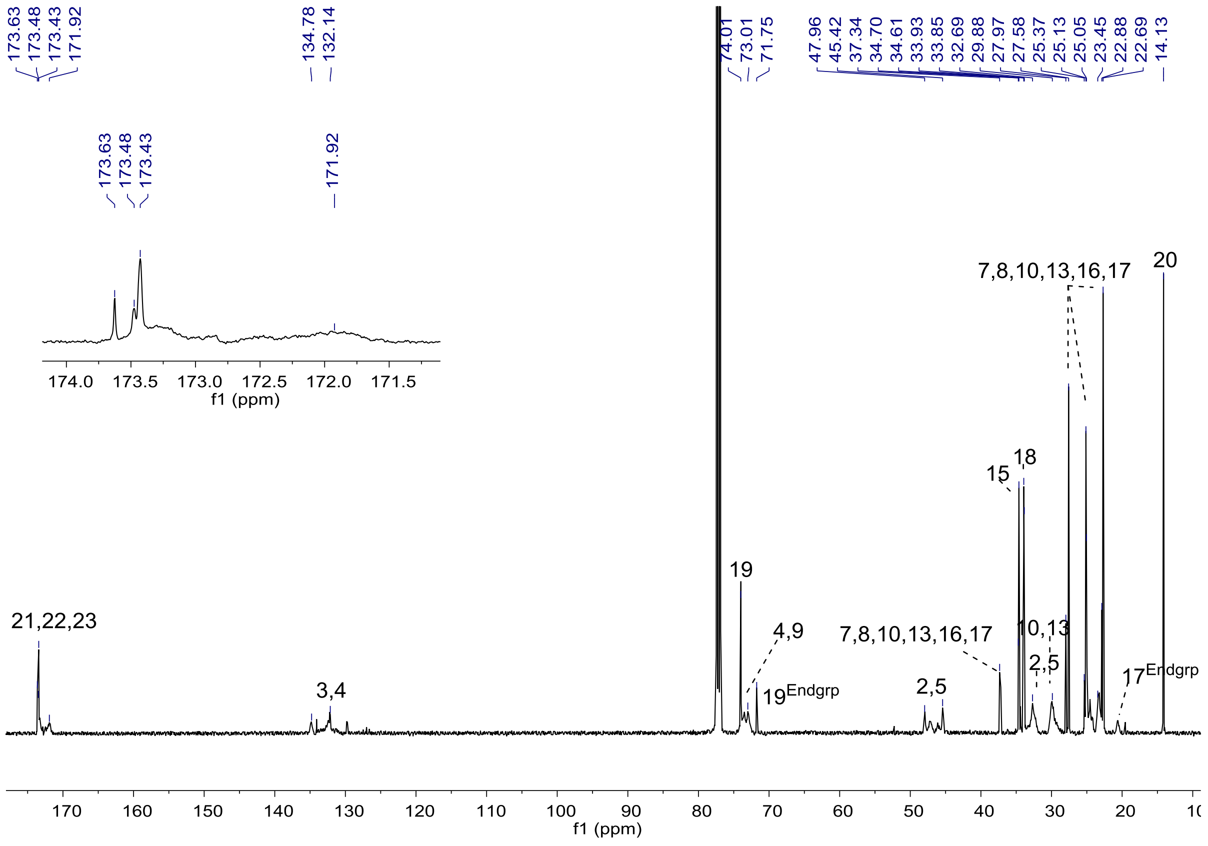

Figure S 59 – $^{13}C\{^{1}H\}$ NMR spectrum of polymer based on TCA2, CHO, DL. Traces of PPNCl were observed at 129.8, 132.2 and 134.1 ppm.

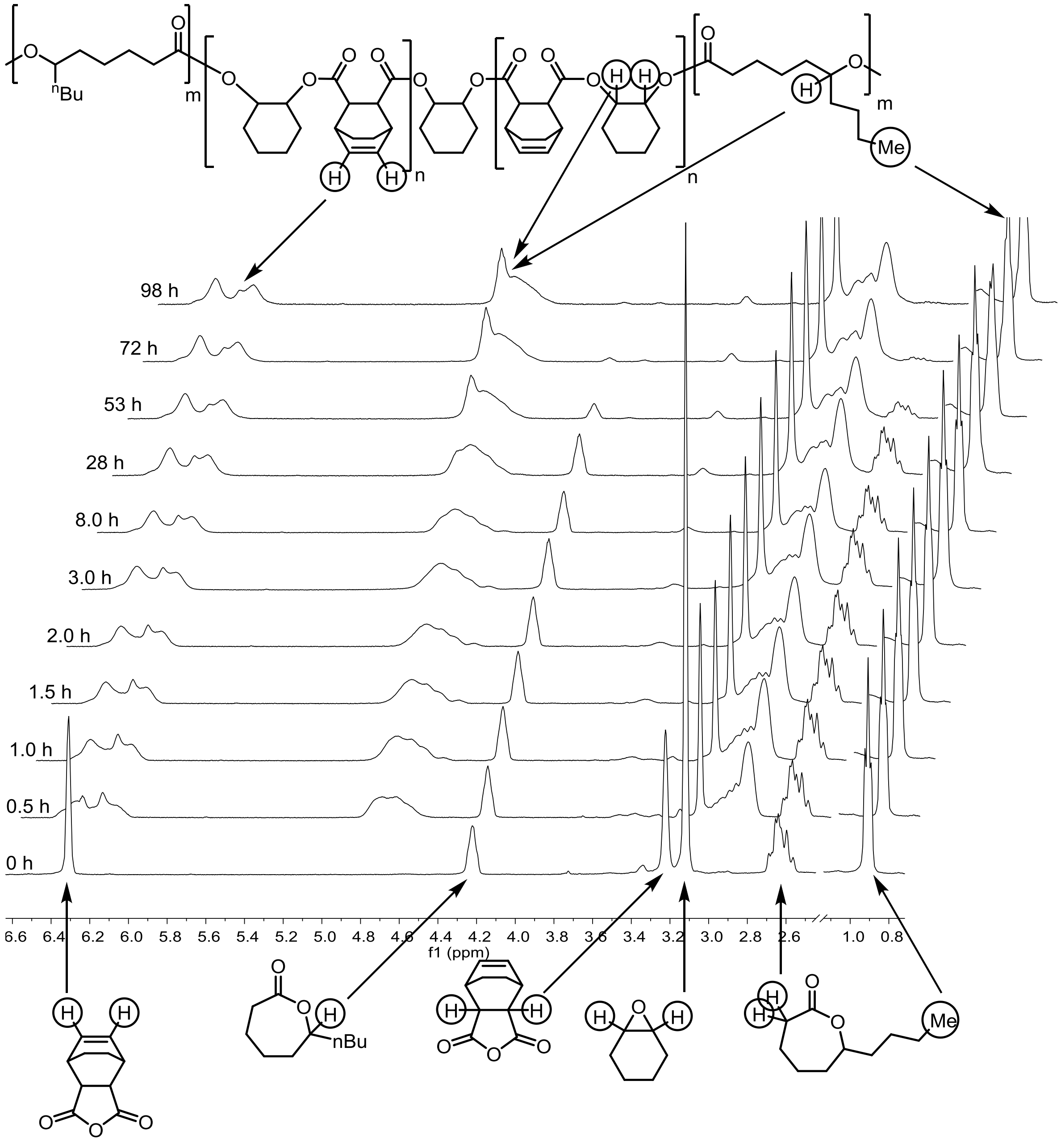

Figure S 60 - ^{1}H NMR spectra of the polymerization of TCA2/CHO/DL.

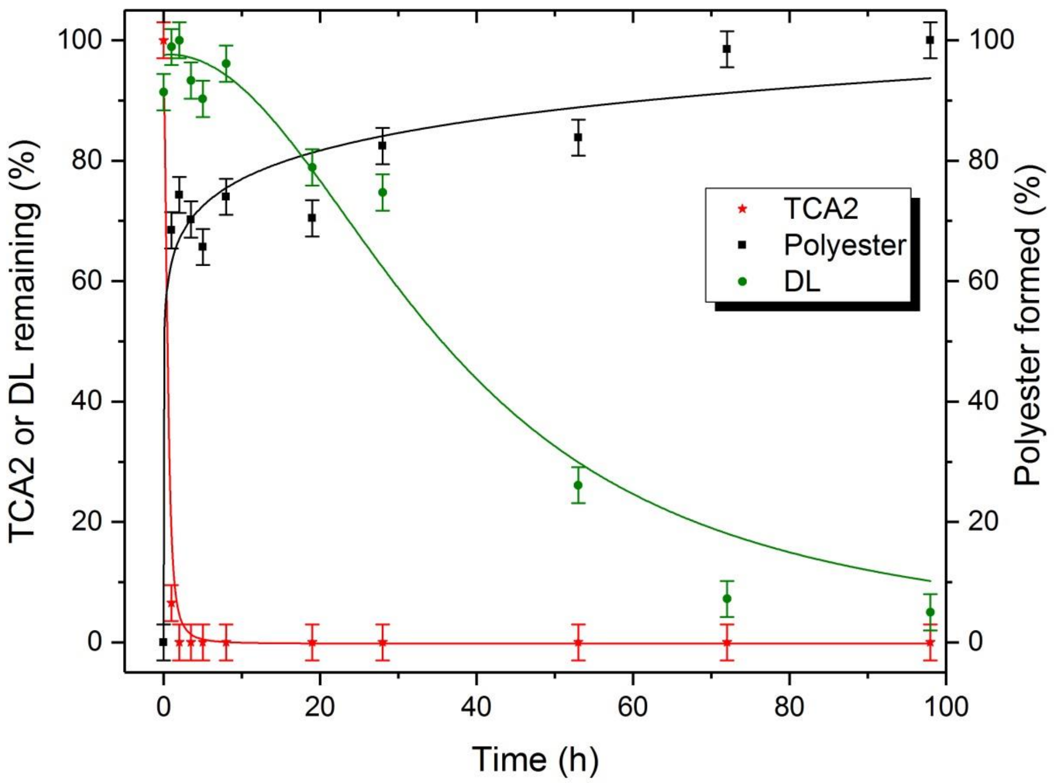

Figure S 61 - Conversion of TCA2 and DL and formation of polyester vs time. Data was obtained after normalization with mesitylene as internal standard.

Table S 8 - Integrals based on ^{1}H NMR spectra (after normalization with mesitylene as internal standard, Figure) and calculated conversions used for Fig. S 68. Signals were based on the following shifts: 6.80 (TCA2), 4.22 (DL), 4.95 – 4.45 (polyester) and 3.10 (CHO) ppm. The conversion of CHO at the end of the reaction was determined as 67 % (data not shown).

Time (h)	Integral TCA2	TCA2 (%)	Integral DL	DL (%)	Integral Polyester	Polyester (%)
0	628	100	329	91.3	0	0
1	41	6.5	356	98.8	690	68.4
2	0	0	360	100	749	74.3
3.5	0	0	336	93.3	708	70.2
5	0	0	325	90.2	662	65.6
8	0	0	346	96.1	746	74.0
19	0	0	284	78.8	710	70.4
28	0	0	269	74.7	831	82.4
53	0	0	94	26.1	845	83.8
72	0	0	26	7.2	993	98.5
98	0	0	18	5	1008	100

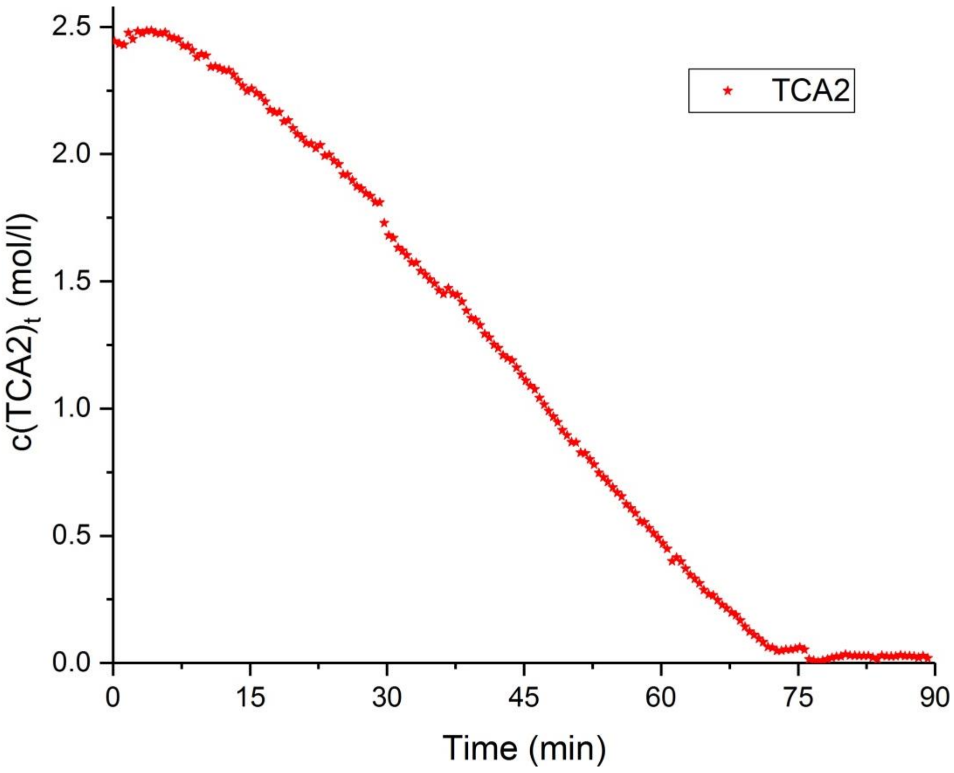

Figure S 62 - *In situ* monitoring of IR band of TCA2 at 1850 cm^{-1}.

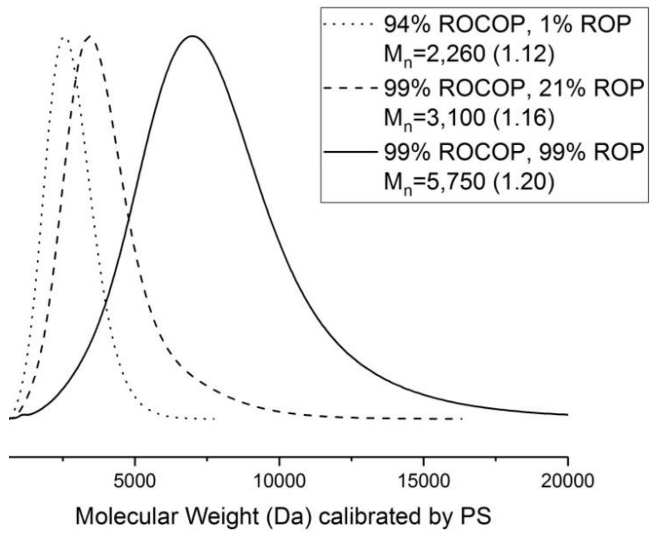

Figure S 63 - GPC chromatograms at different conversions.

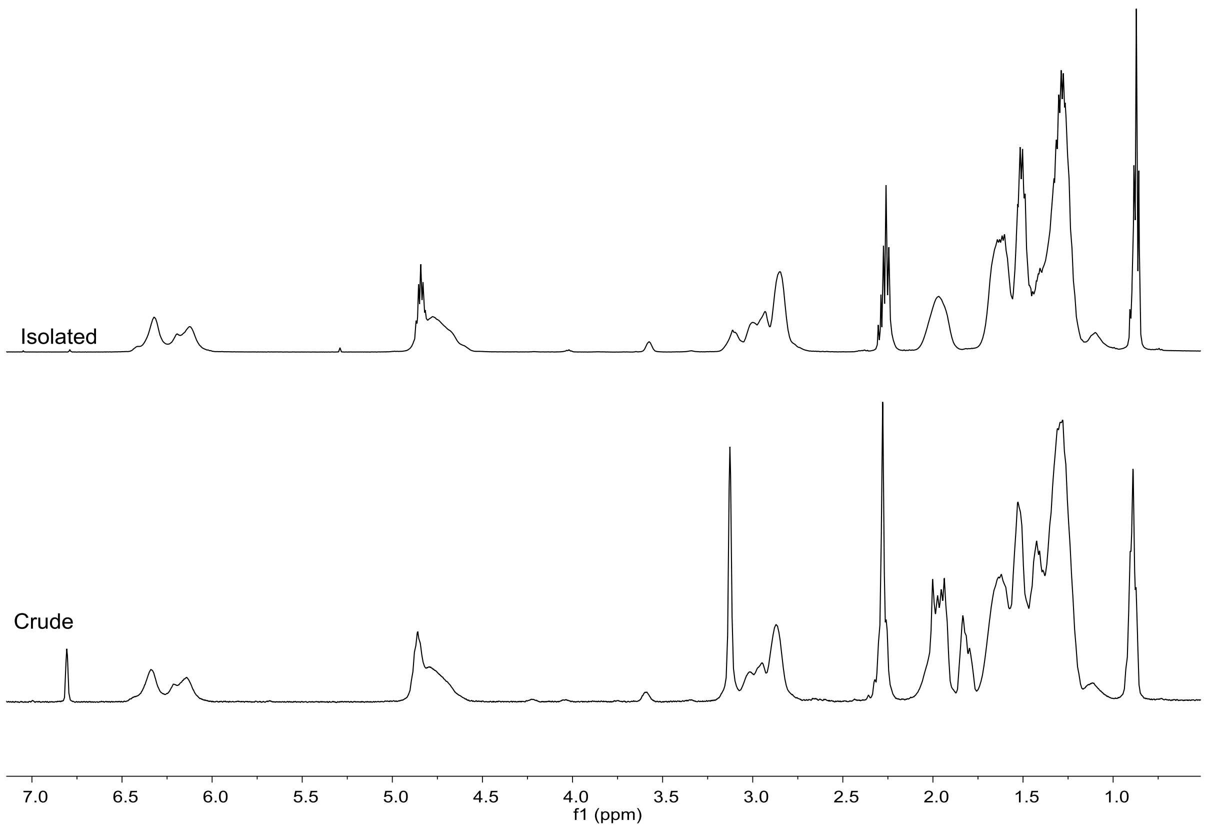

Figure S 64 – ^{1}H NMR spectra of crude (bottom) and isolated (top) polymer based on TCA2/CHO/DL.

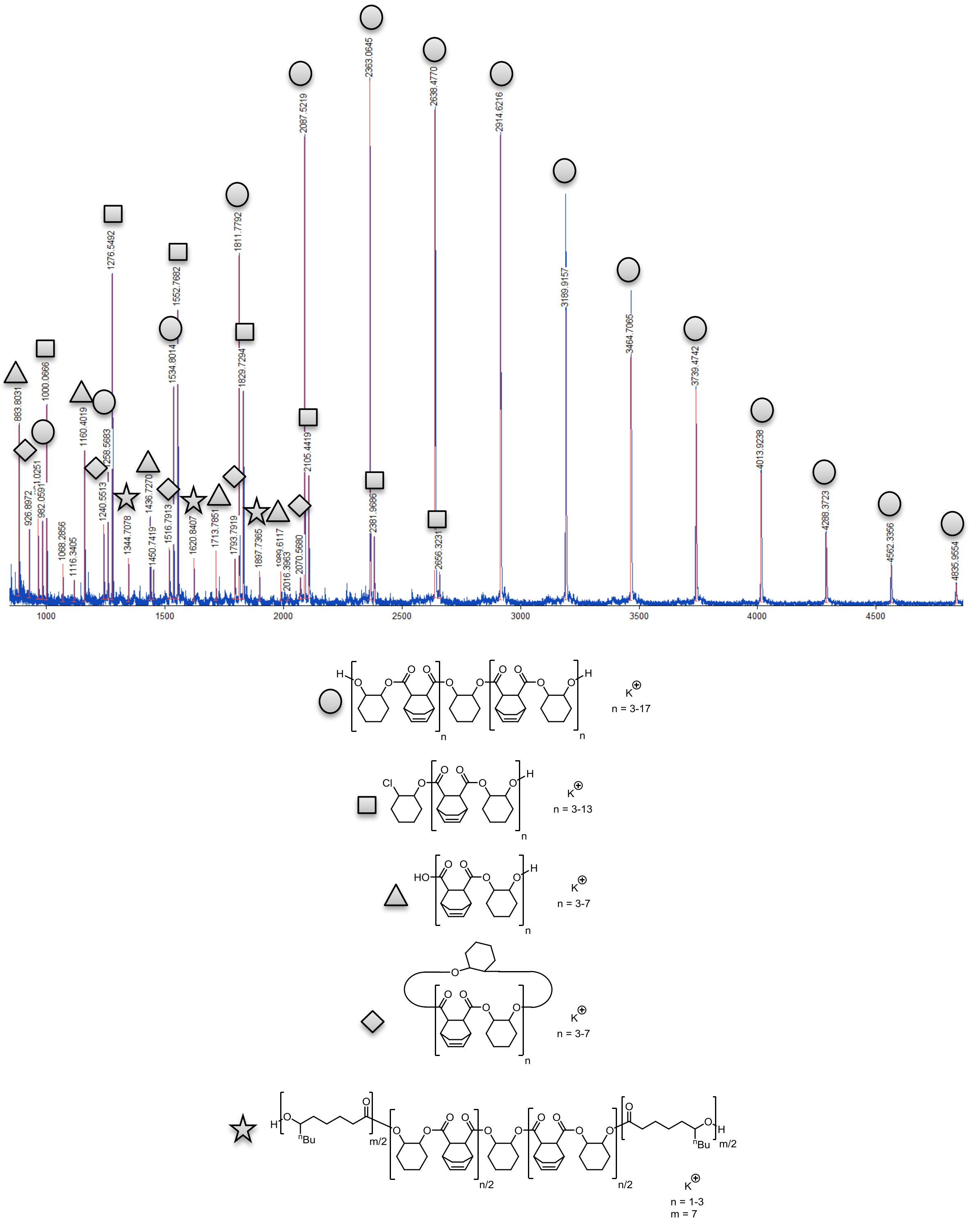

Figure S 65 – MALDI of polymer based on TCA2/CHO/DL (after 1 h, 94% TCA2 and 1% DL conversion). Five species were detected: circle: m/z = [116.08 (cyclohexandiol) + (276.14 x n) (PCHTCE2) + 38.96 (K^+)] (n = 3 ~ 17); square: m/z = [34.97 ($Cl^{\cdot}$) + 98.07 (CHO) + 1.01 ($H^{\cdot}$) + (276.14 x n) (PCHTCE2) + 38.96 (K^+)] (n = 3 ~ 13); triangle: m/z = [18.01 (H_2O) + (276.14 x n) (PCHTCE2) + 38.96 (K^+)] (n = 3 ~ 7); diamond: m/z = [98.07 (CHO) + (276.14 x n) (PCHTCE2) + 38.96 (K^+)] (n = 3 ~ 7); star: m/z = [116.08 (cyclohexandiol) + (214.08 x n) (PCHTCE2) + (170.13 x m) (PDL) + 38.96 (K^+)] (n = 4 ~ 12).

5.5. CA/CHO/DL

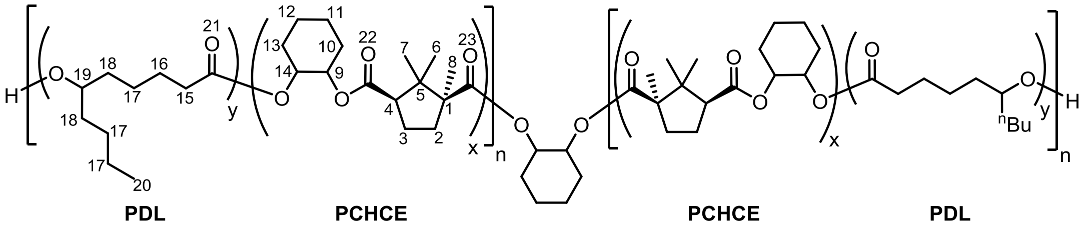

^{1}H NMR (500 MHz, $CDCl_3$) δ 4.83 (m, 1.00 H, $H^{9,14,19}$), 3.56 (m, 0.05 H, $H^{19\text{-Endgroup}}$), 2.69 (m, 0.34 H, H^{4}), 2.50 (m, 0.34 H, H^{2}), 2.25 (m, 0.64 H, H^{15}), 2.10 (m, 0.37 H, H^{3}), 1.99 (m, 0.74 H, $H^{10,13}$), 1.79-1.73 (m, 0.29 H, $H^{3'}$), 1.68 (m, 0.97 H, H^{16}), 1.62-1.55 (m, 0.65 H, H^{16}), 1.50 (m, 1.20 H, H^{18}), 1.46-1.33 (m, 2.24 H, $H^{2',10',13',11,12}$), 1.26 (m, 1.54 H, H^{17}), 1.19-1.13 (m,2.18 H, $H^{6,7,8}$), 0.86 (m, 0.97 H, H^{20}), 0.74 (m, 1.04 H, $H^{6,7,8}$).

^{13}C NMR (126 MHz, $CDCl_3$) δ 175.12 ($C^{22,23}$), 174.92 ($C^{22,23}$), 173.85 (C^{21}), 173.65 (C^{21}), 173.53 ($C^{22,23}$), 173.10 ($C^{22,23}$), 74.21 ($C^{9,14,19}$), 71.95 ($C^{19\text{-Endgroup}}$), 56.64-56.32 (C^{5}), 53.48-53.15 (C^{4}), 46.85 (C^{1}), 37.57-37.40 ($C^{2,10,13}$), 34.93-34.84 (C^{20}), 34.15-34.08 (C^{18}), 32.82 (C^{2}), 30.28 ($C^{10,13}$), 27.80 (C^{17}), 25.60-22.70 ($C^{3,6,7,8,16,18}$), 25.27 ($C^{16,17}$), 22.92 ($C^{11,12}$), 21.91 ($C^{6,7,8}$), 21.43 ($C^{6,7,8}$), 14.36 (C^{20}).

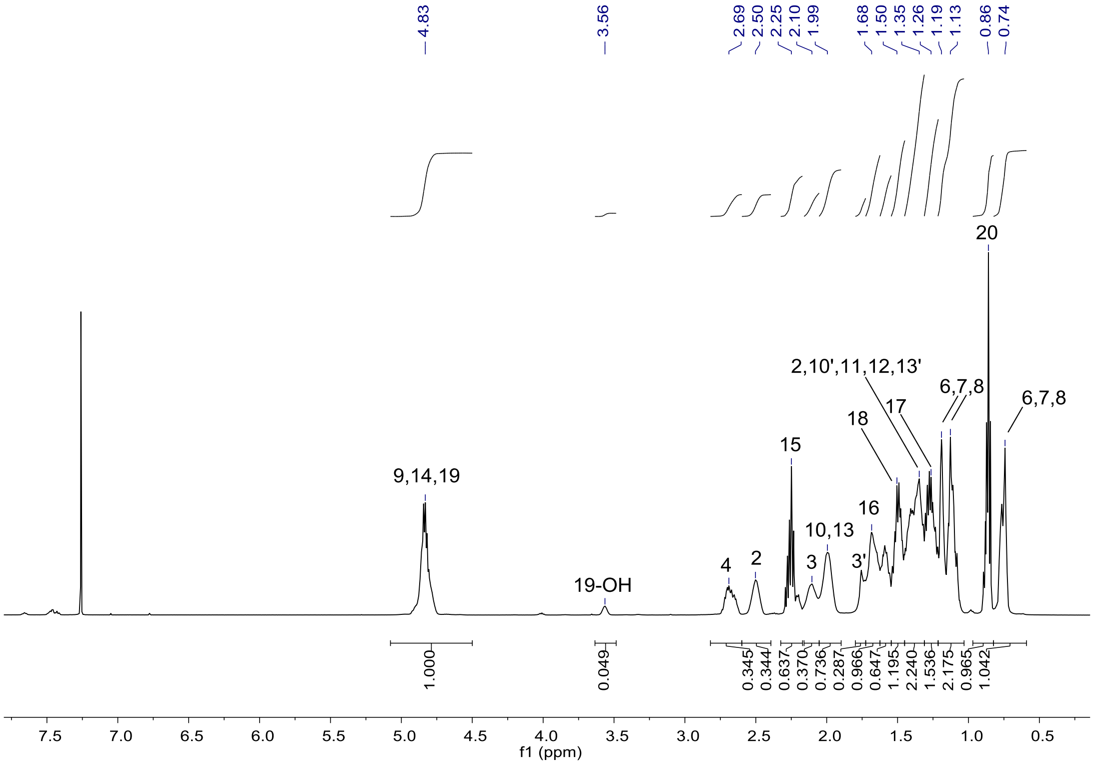

Figure S 66 –^{1}H NMR spectrum of polymer based on CA/CHO/DL.

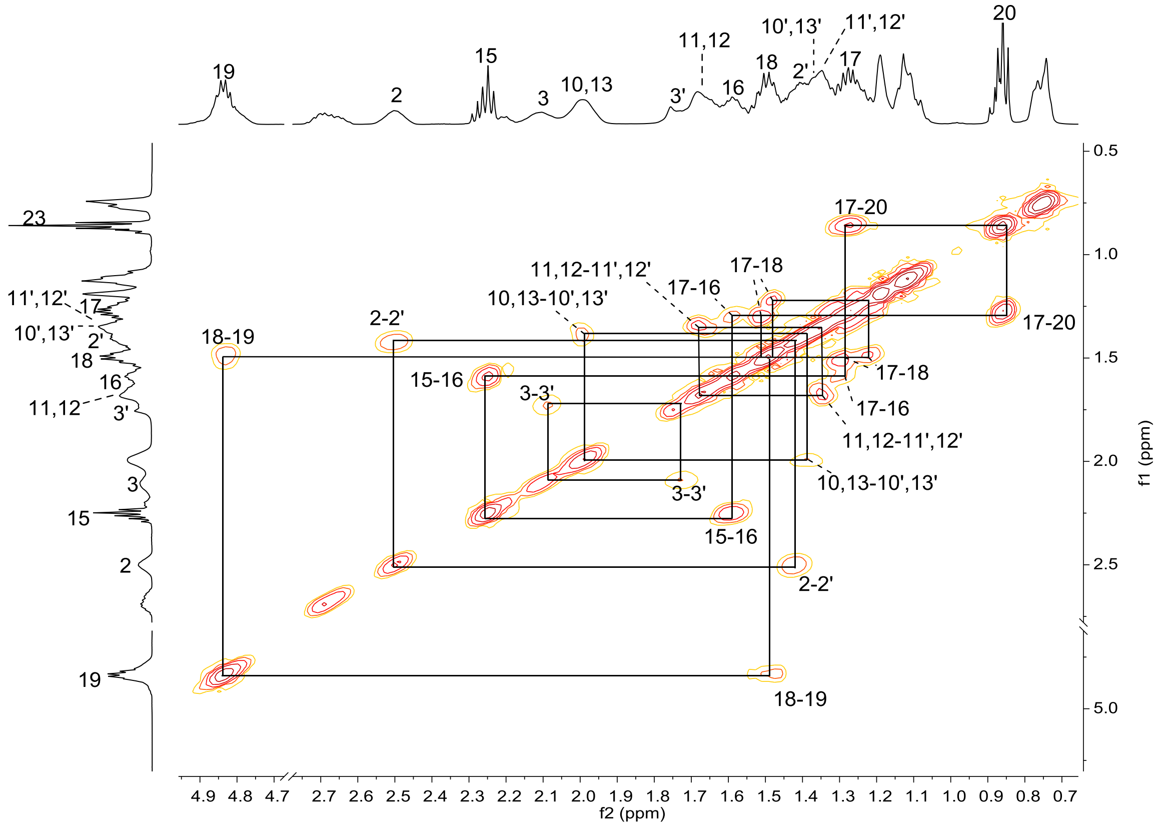

Figure S 67 - COSY NMR spectrum for polymer based on CA/CHO/DL.

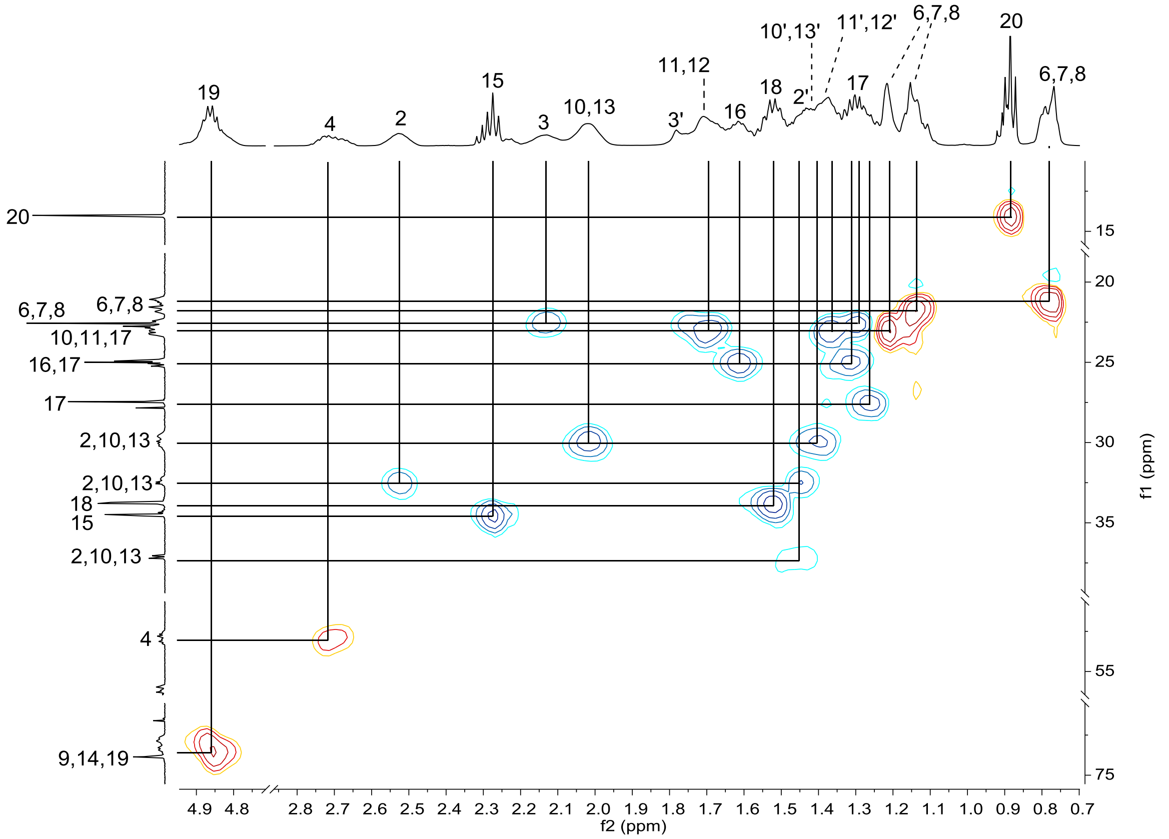

Figure S 68 - HSQC NMR spectrum for polymer based on CA/CHO/DL.

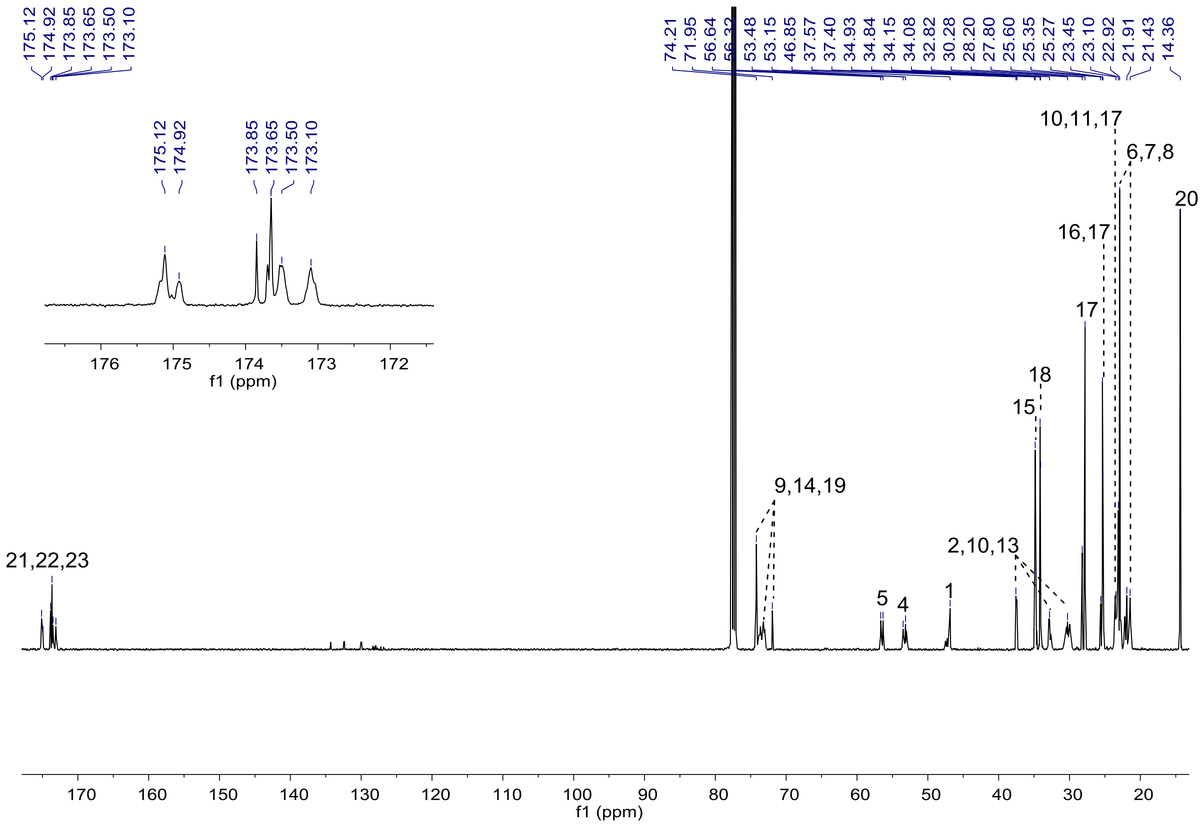

Figure S 69 - $^{13}C\{^{1}H\}$ NMR spectrum based on CA/CHO/DL. Traces of PPNCl were observed at 129.8, 132.2 and 134.1 ppm.

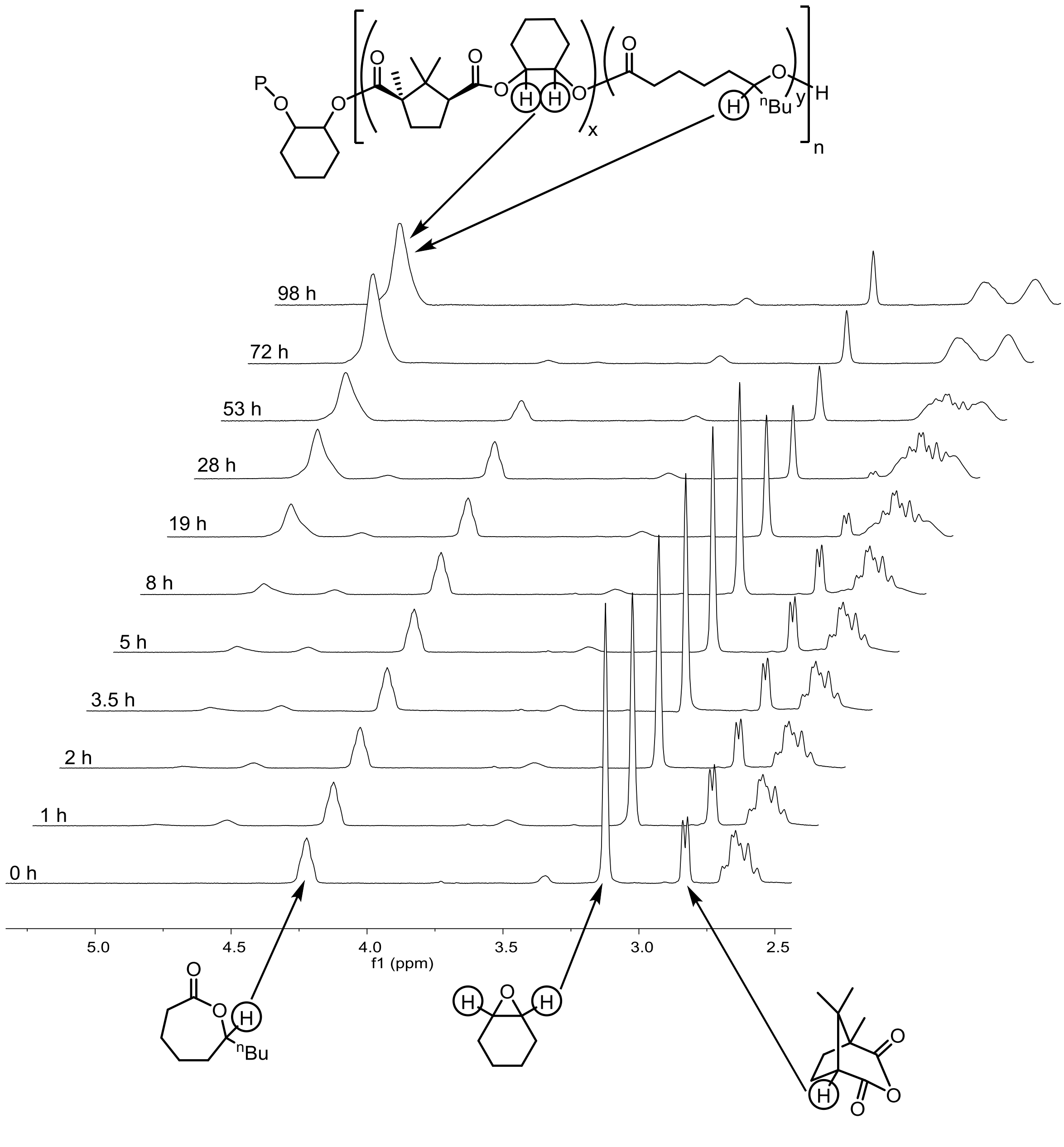

Figure S 70 – ^{1}H NMR spectra for the polymerization of CA, CHO and DL.

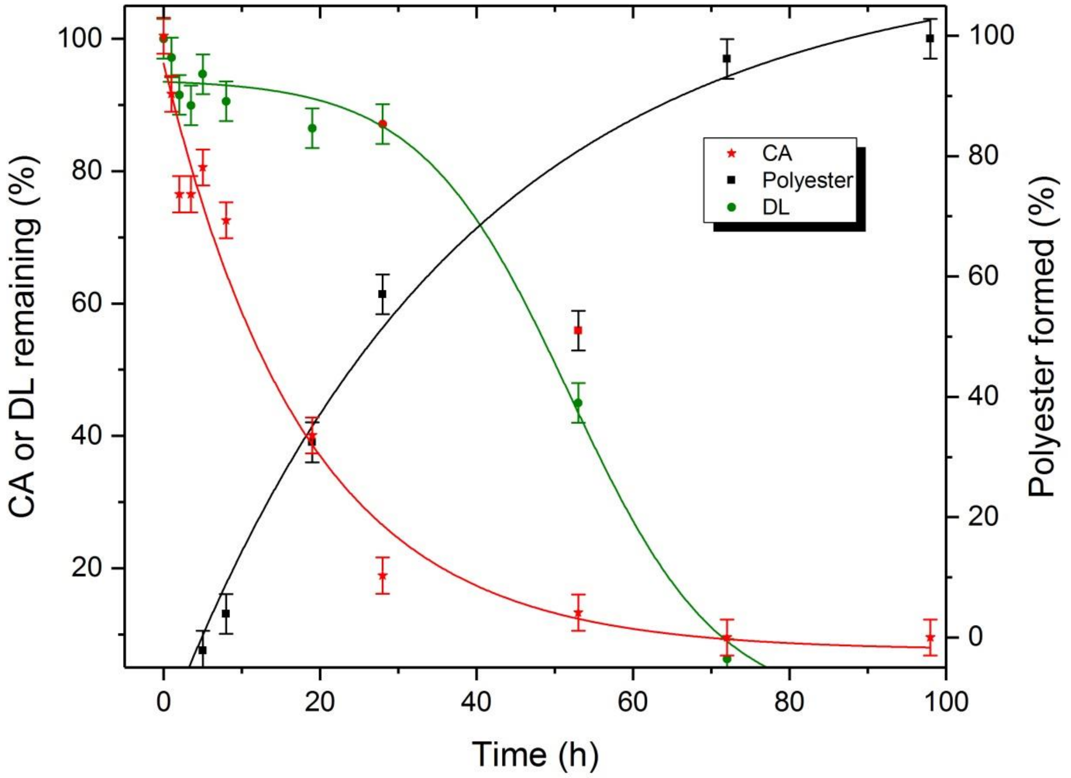

Figure S 71 - Conversion of CA, CHO and DL and formation of polyester.

Table S 9 - Integrals based on ^{1}H NMR spectra (after normalization with mesitylene as internal standard, Figure S 70) and calculated conversions used for Figure S 71. Signals were based on the following shifts: 1.00 (CA), 3.13 (CHO), 4.22 (DL), 4.74 – 5.00 (polyester) ppm. The conversion of CHO at the end of the reaction was determined as 80 % (data not shown).

Time (h)	Integral CA	CA (%)	Integral DL	DL (%)	Integral Polyester	Polyester (%)
0	923	100	318	100	0	0
1	834	90.3	309	97.1	13	1.4
2	680	73.6	291	91.5	16	1.7
3.5	680	73.6	286	89.9	24	2.5
5	721	78.1	301	94.6	70	7.5
8.5	640	69.3	288	90.5	121	13.0
19	310	33.5	275	86.4	361	39.0
28	95	10.2	277	87.1	568	61.41
53	38	4.1	143	44.9	517	55.8
72	0	0	20	6.2	897	96.9
95	0	0	4	1.2	925	100

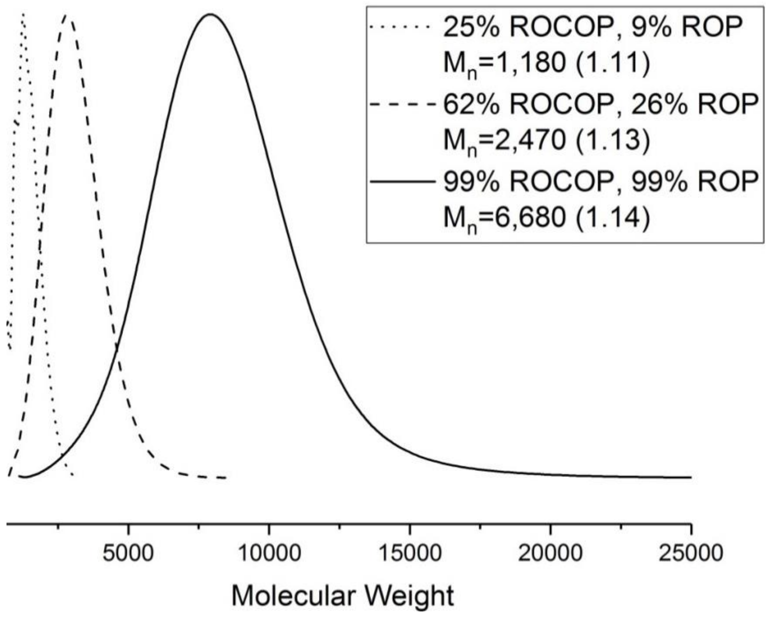

Figure S 72 - GPC chromatograms at different conversions for the polymerization of CA/CHO/DL.

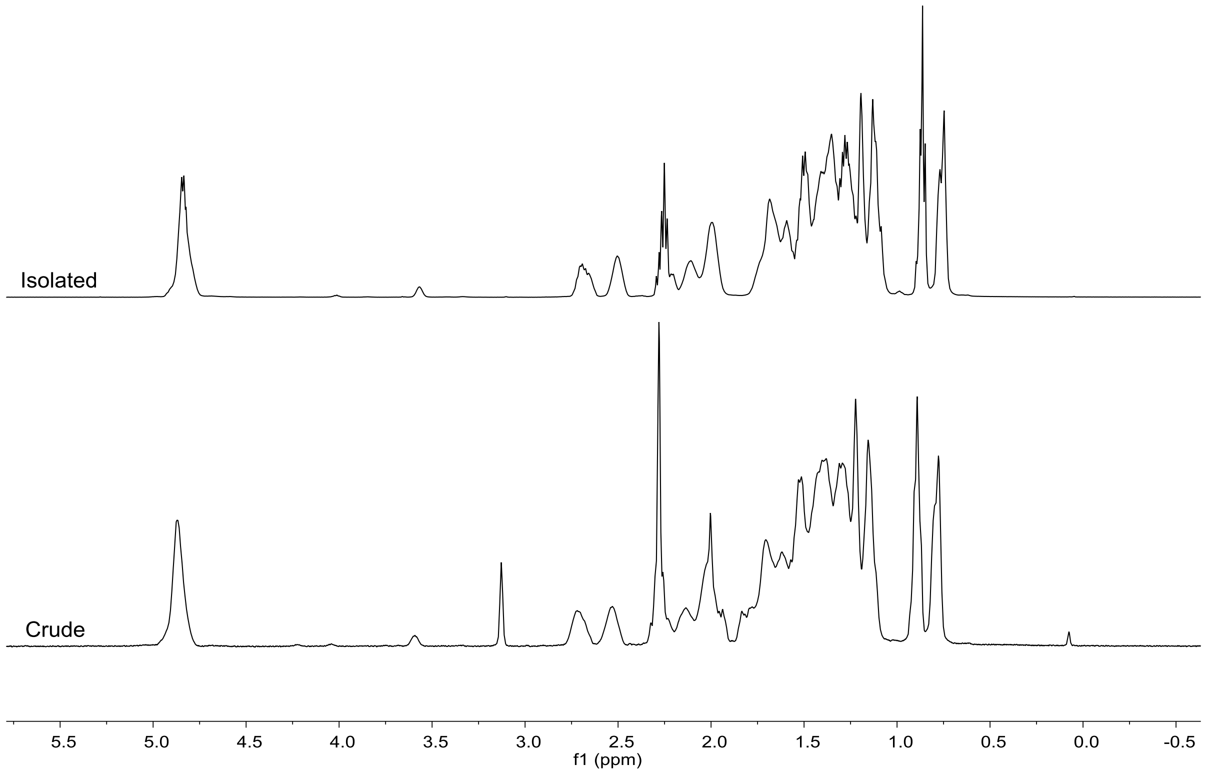

Figure S 73 - ^{1}H NMR spectra of crude (bottom) and isolated (top) polymer based on CA/CHO/DL.

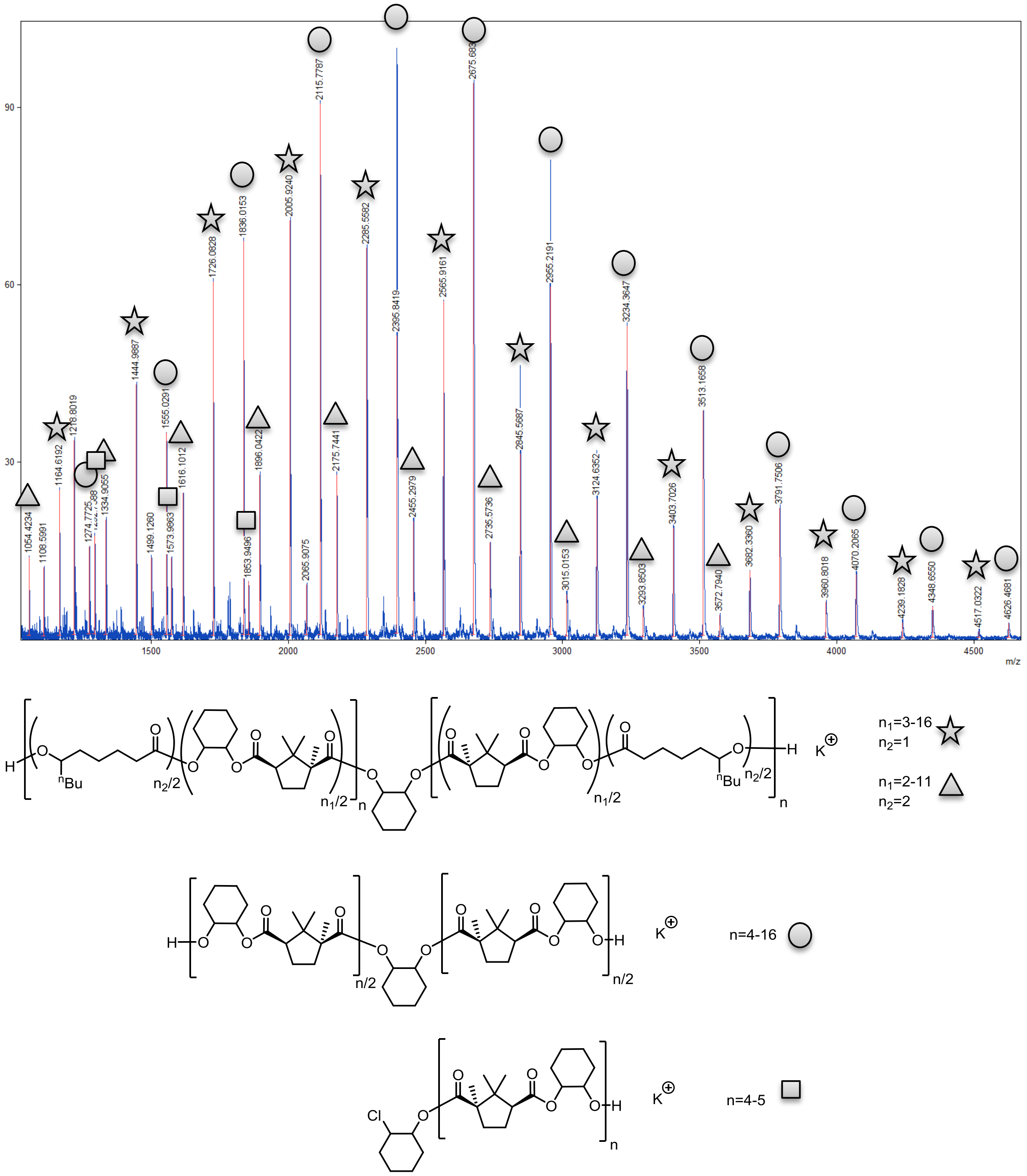

Figure S 74– MALDI of one-pot polymerization of CA/CHO/DL (after 19 h, 67% CA and 14% DL conversion). The series shows four species: star and triangle: m/z = [116.08 (cyclohexandiol) + (280.17 x n) (PCHCE) + (170.13 x m) (PDL) + 38.96 (K^+)] (n = 3 ~ 16 and m = 1 for star and n = 2 ~ 11 and m = 2 for triangle); circle: m/z = [116.08 + (cyclohexandiol) + (280.17 x n) (PCHCE) + 38.96 (K^+)] (n = 4 ~ 16); square: m/z = [34.97 ($Cl^{\cdot}$) + 98.07 (CHO) + 1.01 ($H^{\cdot}$) + (280.17 x n) (PCHCE) + 38.96 (K^+)] (n = 4 ~ 5).

5.6. End-group analysis with $^{31}P\{^1H\}$ NMR spectroscopy

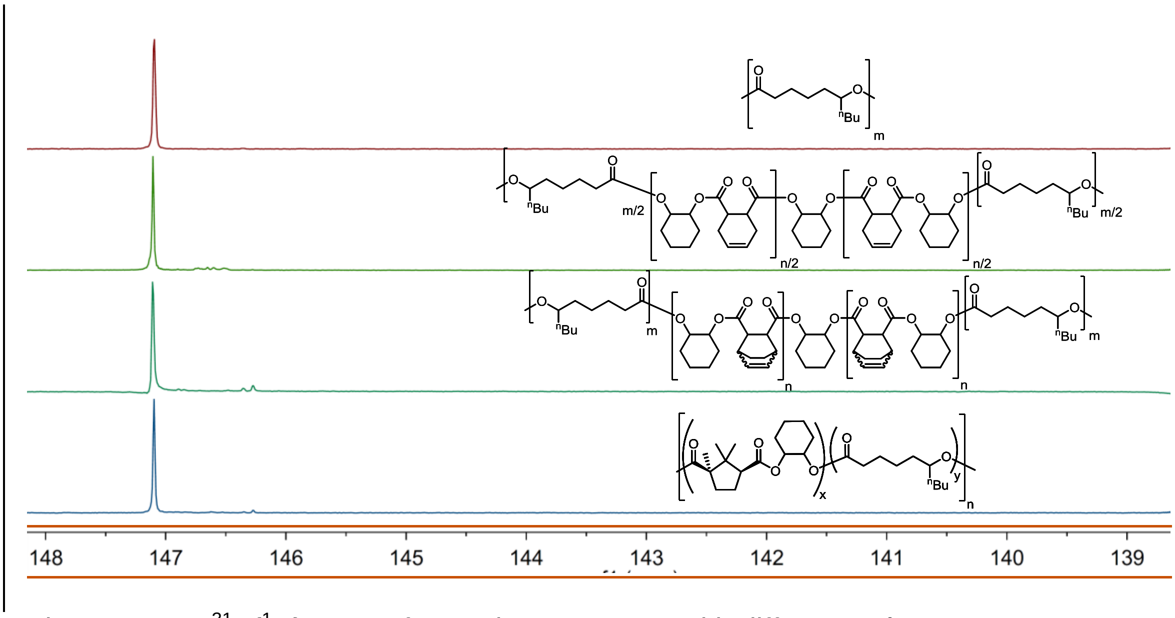

Figure S 75 – $^{31}P\{^1H\}$ NMR after end-group assay with different polymers.

Table S 10 – $^{31}P\{^1H\}$ NMR chemical shift of different polymers after end-group assay.

Anhydride in Backbone	$^{31}P\{^1H\}$ Shift (ppm)	Integral-ratio PDL:ROCOP
THPA	146.6	86:14
TCA2	146.2, 146.3	92:8
CA	146.3	98:2

6. Post-Modification Reactions

6.1. Thiol-Ene Reactions

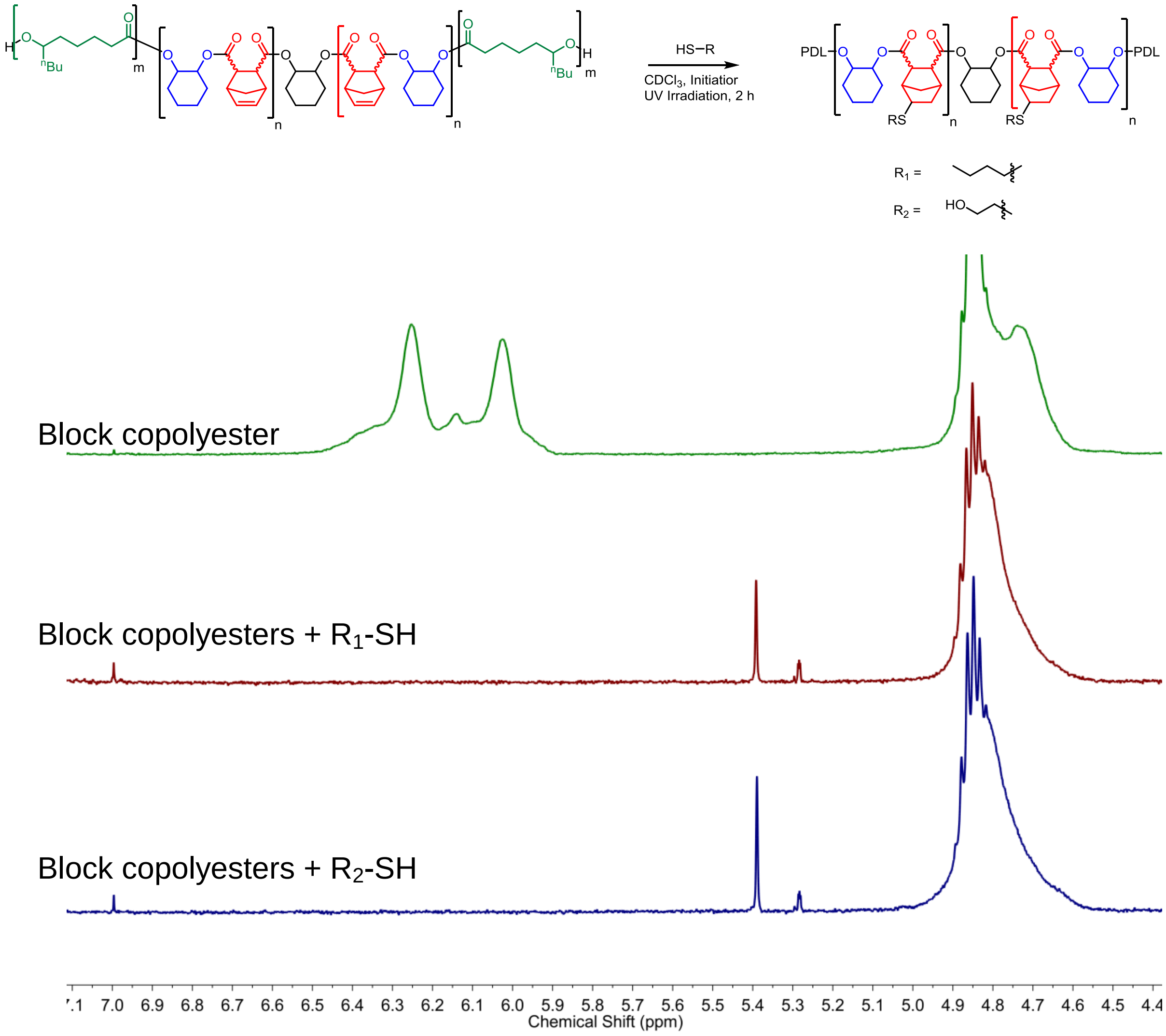

Figure S 76 – ^{1}H NMR spectra of curde samples before and after thiol-ene reactions.

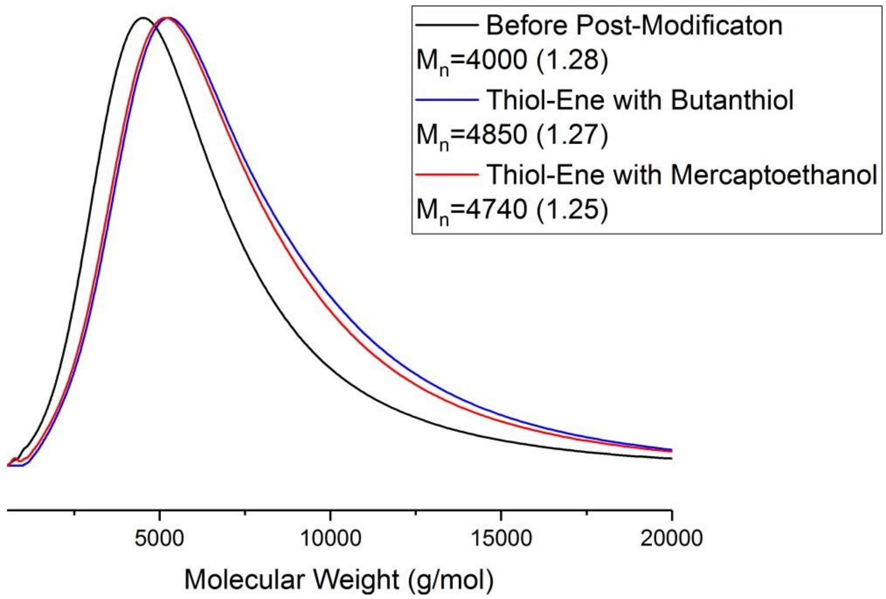

Figure S 77 – GPC Chromatograms before and after thiol-ene reactions (molecular weights are based on PS standards).

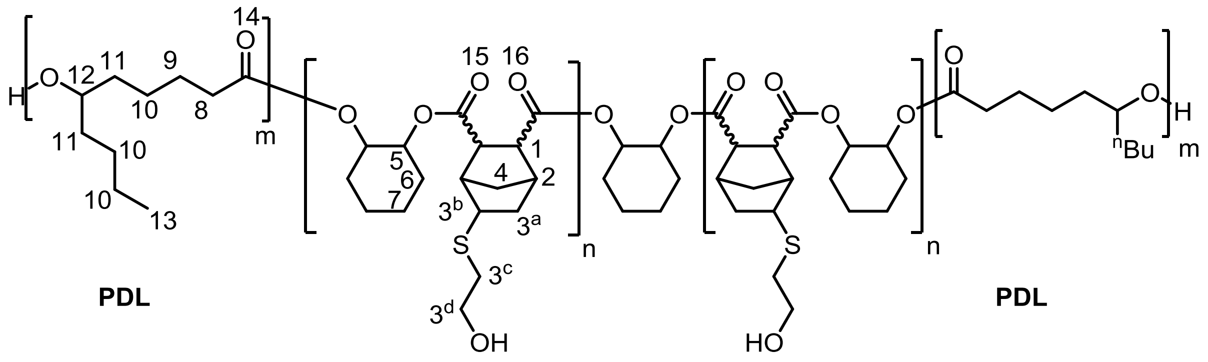

^1H NMR (400 MHz, $CDCl_3$) δ 5.00-4.53 (m, 1.00 H, $H^{5,12}$), 3.93-3.62 (m, 0.84 H, H^{3d}), 3.62-3.48 (m, 0.15 H, $H^{12\text{-Endgrp}}$), 3.47-3.02 (m, 0.61 H, $H^{1,2}$), 2.97-2.34 (m, 2.47 H, $H^{3a,b,c,b\text{-OH}}$), 2.34 – 2.13 (m, 0.61 H, H^{8}), 2.12-1.84 (m, 1.03 H, H^{6}), 1.84-1.55 (m, 1.98 H, $H^{7,9}$), 1.55-1.44 (m, 1.14 H, H^{11}), 1.44-0.96 (m, 4.30 H, $H^{4,4',6',7',10,11\text{-Endgrp}}$), 0.93-0.73 (m, 0.90 H, H^{13}).

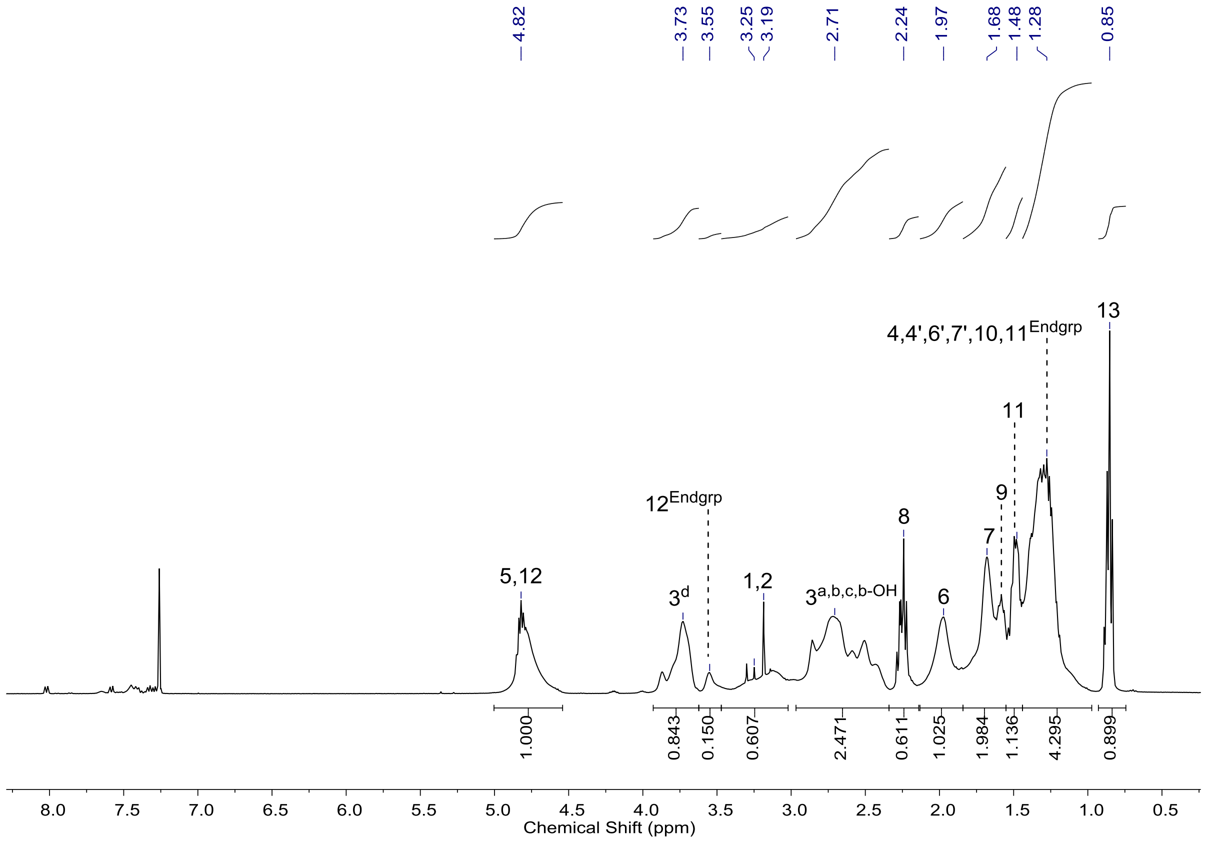

Figure S 78 – ^1H NMR spectrum of isolated polyester after post-functionalization with mercapto ethanol.

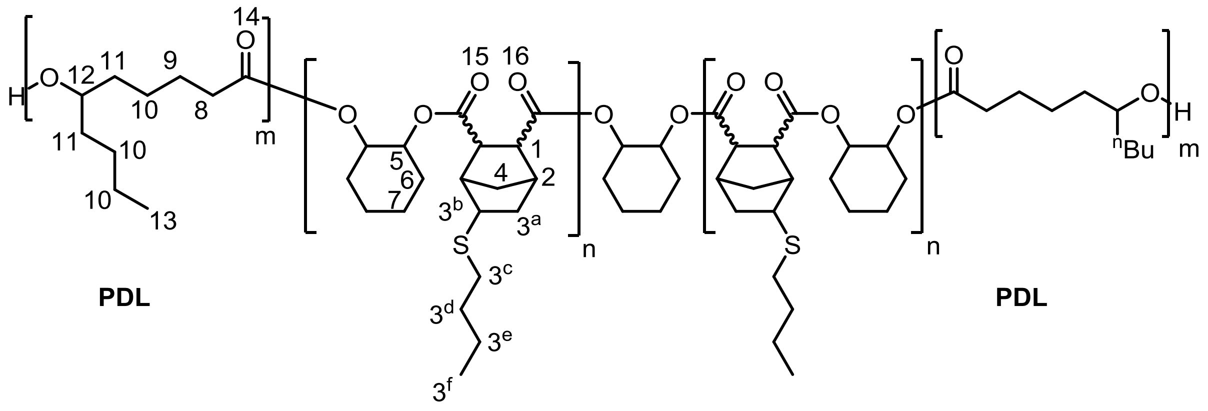

^{1}H NMR (400 MHz, $CDCl_3$) δ 4.92-4.47 (m, 1.00 H, $H^{5,12}$), 3.60-3.31 (m, 0.20 H, $H^{12\text{-Endgrp}}$), 3.30-2.77 (m, 0.71 H, $H^{1,2}$), 2.76-2.29 (m, 2.04 H, $H^{3a,b,c}$), 2.31 – 2.10 (m, 0.45 H, H^{8}), 2.10-1.81 (m, 0.98 H, H^{6}), 1.82-1.41 (m, 3.49 H, $H^{3d,e,7,9,11}$), 1.41-0.96 (m, 4.48 H, $H^{3f,4,4',6',7',10,11\text{-Endgrp}}$), 0.94-0.70 (m, 1.73 H, $H^{3f,13}$).

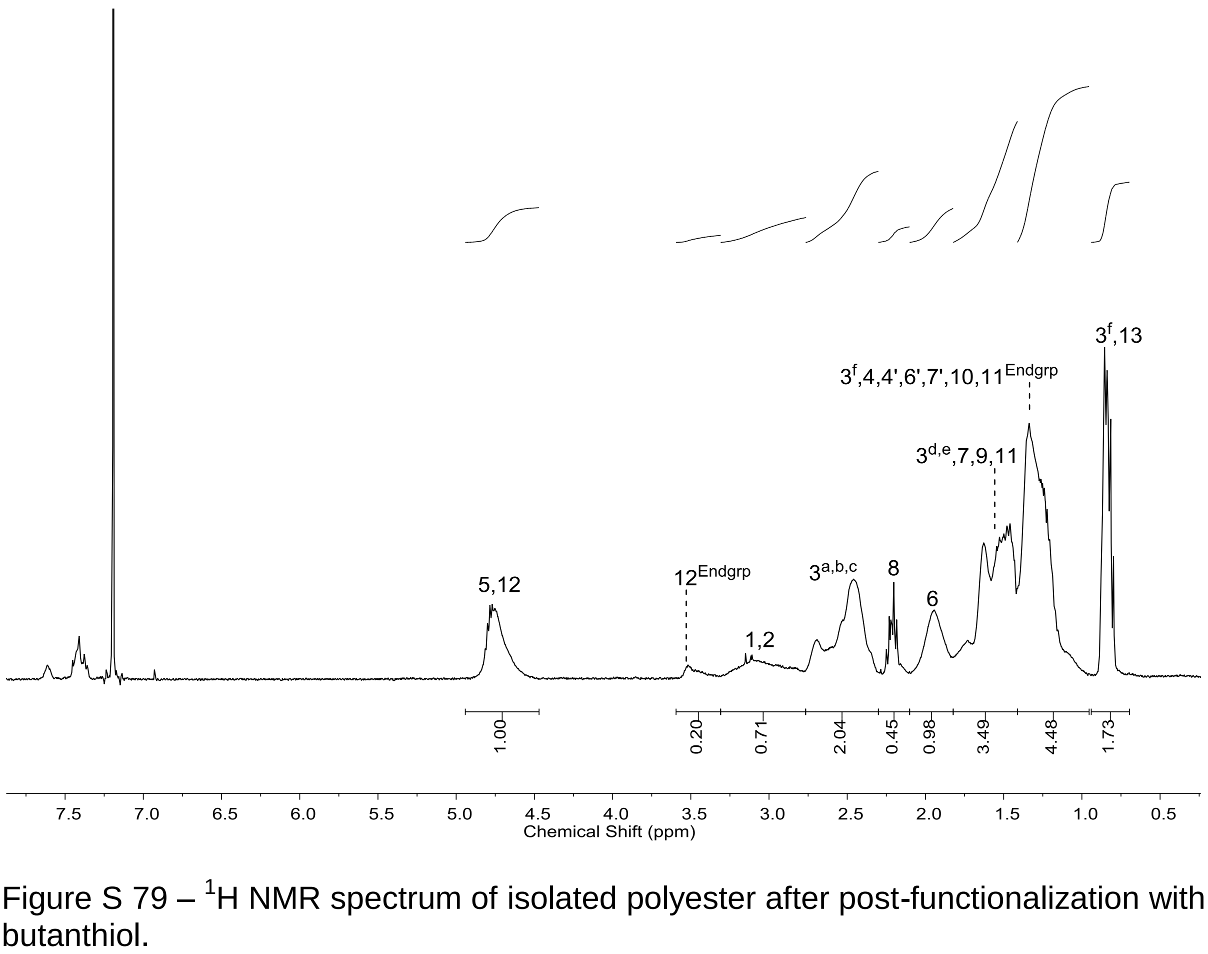

Figure S 79 – ^{1}H NMR spectrum of isolated polyester after post-functionalization with butanthiol.

6.2. Reaction with Methylene diphenyl diisocyanate

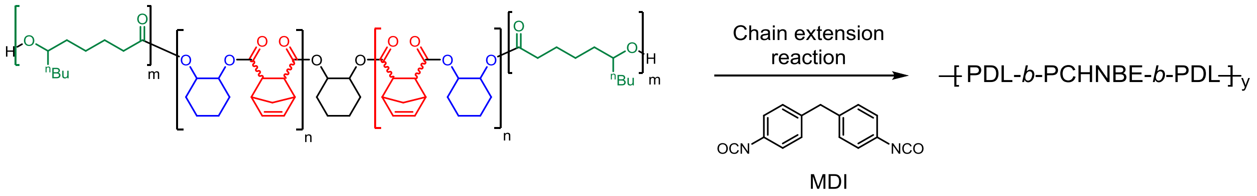

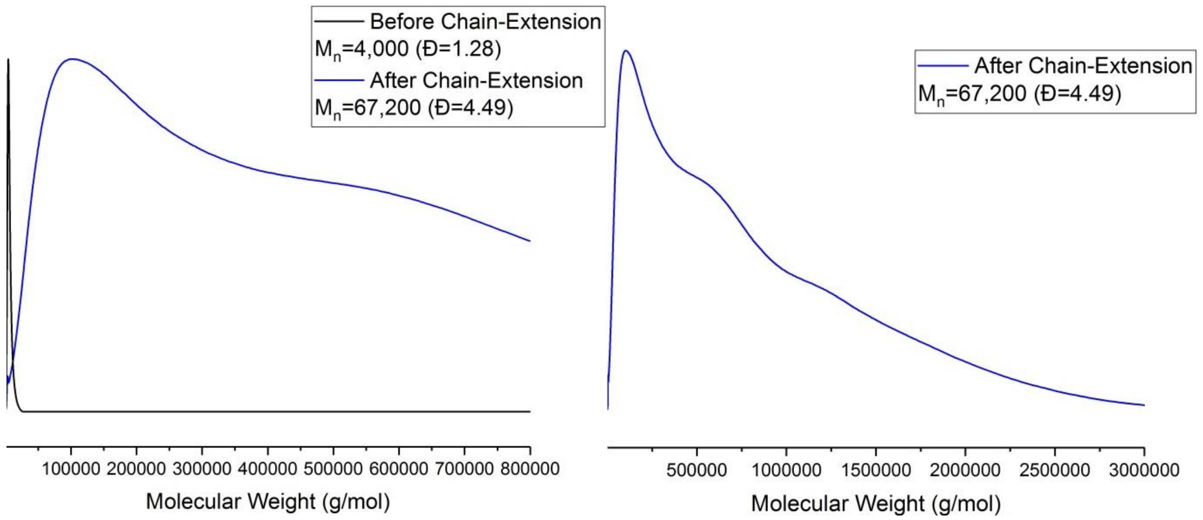

Figure S 80 – Representative GPC chromatograms before and after chain-extension with MDI (left). Molecular weights are given relative to polystyrene.

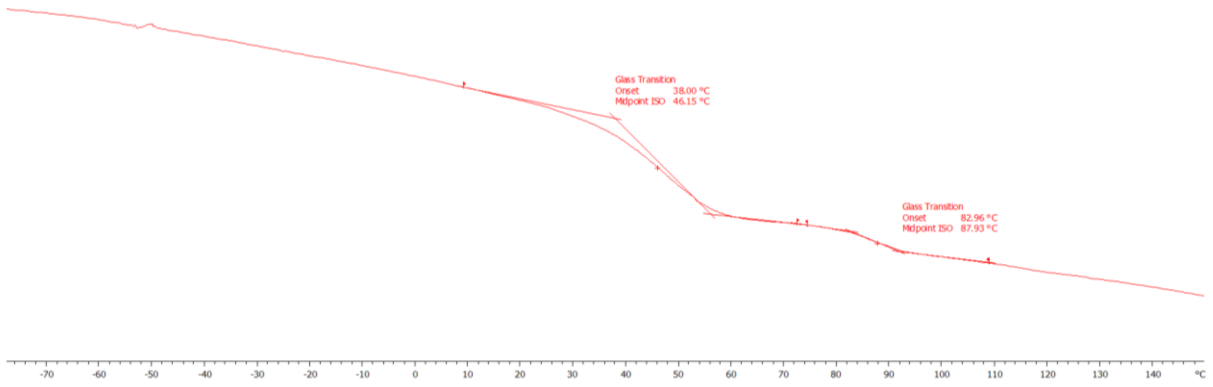

Figure S 81 – DSC Thermogram of chain-extended block copolyester (3rd heating cycle reported).

7. References

1. Hauenstein, O.; Reiter, M.; Agarwal, S.; Rieger, B.; Greiner, A., *Green Chem.* **2016,** *18* (3), 760-770.
2. Fulmer, G. R.; Miller, A. J. M.; Sherden, N. H.; Gottlieb, H. E.; Nudelman, A.; Stoltz, B. M.; Bercaw, J. E.; Goldberg, K. I., *Organometallics* **2010,** *29* (9), 2176-2179.
3. Spyros, A.; Argyropoulos, D. S.; Marchessault, R. H., *Macromolecules* **1997,** *30* (2), 327-329.
4. (a) Saini, P. K.; Fiorani, G.; Mathers, R. T.; Williams, C. K., *Chem. - Eur. J.* **2017,** *23* (18), 4260-4265; (b) Zhu, Y.; Romain, C.; Poirier, V.; Williams, C. K., *Macromolecules* **2015,** *48* (8), 2407-2416.
5. Wang, L.; Poirier, V.; Ghiotto, F.; Bochmann, M.; Cannon, R. D.; Carpentier, J.-F.; Sarazin, Y., *Macromolecules* **2014,** *47* (8), 2574-2584.
6. Van Zee, N. J.; Coates, G. W., *Angew. Chem., Int. Ed.* **2015,** *54* (9), 2665-2668.
7. Boratyński, F.; Pannek, J.; Walczak, P.; Janik-Polanowicz, A.; Huszcza, E.; Szczepańska, E.; Martinez-Rojas, E.; Olejniczak, T., *Process Biochem.* **2014,** *49* (10), 1637-1646.
8. Van Zee, N. J.; Sanford, M. J.; Coates, G. W., *J. Am. Chem. Soc.* **2016,** *138* (8), 2755-2761.
